# The association between single nucleotide polymorphisms and ovarian cancer risk: A systematic review and network meta‐analysis

**DOI:** 10.1002/cam4.4891

**Published:** 2022-05-30

**Authors:** Jia Hu, Zhe Xu, Zhuomiao Ye, Jin Li, Zhinan Hao, Yongjun Wang

**Affiliations:** ^1^ Department of Gastroenterology The Second Xiangya Hospital, Central South University Changsha China; ^2^ Research Center of Digestive Disease The Second Xiangya Hospital, Central South University Changsha China; ^3^ Department of Pharmacy, Xiangya Hospital Central South University Changsha China; ^4^ Department of Oncology, Xiangya Hospital Central South University Changsha China; ^5^ Xiangya School of Medicine Central South University Changsha China; ^6^ Department of Gastrointestinal Surgery Hebei General Hospital Shijiazhuang China

**Keywords:** network meta‐analysis, ovarian cancer, single nucleotide polymorphisms, systematic review

## Abstract

**Background:**

The relationship between single nucleotide polymorphisms (SNPs) and ovarian cancer (OC) risk remains controversial. This systematic review and network meta‐analysis was aimed to determine the association between SNPs and OC risk.

**Methods:**

Several databases (PubMed, EMBASE, China National Knowledge Infrastructure, Wanfang databases, China Science and Technology Journal Database, and China Biology Medicine disc) were searched to summarize the association between SNPs and OC published throughout April 2021. Direct meta‐analysis was used to identify SNPs that could predict the incidence of OC. Ranking probability resulting from network meta‐analysis and the Thakkinstian’s algorithm was used to select the most appropriate gene model. The false positive report probability (FPRP) and Venice criteria were further tested for credible relationships. Subgroup analysis was also carried out to explore whether there are racial differences.

**Results:**

A total of 63 genes and 92 SNPs were included in our study after careful consideration. Fok1 rs2228570 is likely a dominant risk factor for the development of OC compared to other selected genes. The dominant gene model of Fok1 rs2228570 (pooled OR = 1.158, 95% CI: 1.068–1.256) was determined to be the most suitable model with a FPRP <0.2 and moderate credibility.

**Conclusions:**

Fok1 rs2228570 is closely linked to OC risk, and the dominant gene model is likely the most appropriate model for estimating OC susceptibility.

## Graphical abstract

In this review, we combined network meta‐analysis, ranking probability analysis and the Thakkinstian’s algorithm to explore the ovarian cancer (OC) risk from the genetic perspective. We found that Fok1 rs2228570 is closely linked to OC risk, and the dominant gene model is likely the most appropriate model for estimating OC susceptibility.

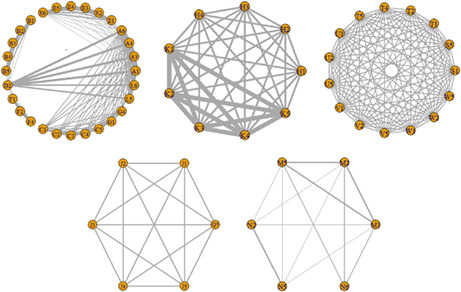

## 1 INTRODUCTION

Ovarian cancer (OC) has the third highest incidence rate among gynecological cancer rankings after cervical and endometrial carcinomas.^1^ OC has the highest mortality rate among all gynecological cancers.^2^ In women, 2.5% of malignancies were OC but cancer‐related deaths were twice as likely caused by OC.^3^ Early detection is of great importance because OC diagnosed at an early stage has a 93% 5‐year survival rate.^3^ Over the past several decades, the number of lifetime ovulations, family history, smoking, benign gynecological conditions, parity, and oral contraceptive use are risk factors for OC.^4^, ^5^ Combining the above‐mentioned factors and genetic mutations contributes to the early detection of OC.

Single nucleotide polymorphisms (SNPs) are the most common type of genetic mutations.^6^ These changes can lead to the occurrence of disease. Variant SNPs, such as BRCA1, BRCA2, IL1A, TNFSF10, CDKN2A, CDKN1B, XRCC2, XRCC3, and DNMT1 have been reported to be associated with OC risk.^5^, ^7^, ^8^, ^9^, ^10^ However, studies on the association between SNPs and OC lack representativeness due to the limited study population and inconsistent results.^9^, ^11^ Generally, OC prognosis is poor. Therefore, it is necessary to identify SNPs that are associated with OC susceptibility. Several reviews have explored the relationship between specific SNPs and OC risk.^12^, ^13^, ^14^, ^15^, ^16^ These changed SNPs alter the function of proteins in different pathways, including cell proliferation and tumorigenic processes.^17^, ^18^, ^19^ However, to our knowledge, no review has comprehensively summarized and evaluated all OC risk‐related SNPs. Therefore, to comprehensively assess the relationships between SNPs and OC risk, this review and network meta‐analysis was conducted based on reported studies, which can provide guidelines for clinical practice.

In this review, we aim to comprehensively evaluate significant OC risk‐related SNPs and to select the most appropriate gene model of SNPs to predict OC susceptibility. Six gene models were tested by comparing the results of the network meta‐analysis and Thakkinstian’s algorithm. In addition, the false positive report probability (FPRP) and Venice criteria were calculated to verify the reliability. Subgroup analysis was further performed on the most appreciate gene model due to the genetic background variation of different races. Diagnostic meta‐analysis was carried out to verify the accuracy of selected SNPs for predicting OC susceptibility.

## 2 MATERIALS AND METHODS

### 2.1 Search strategy and inclusion criteria

Jia Hu, Zhe Xu, and Zhuomiao Ye searched the published study. Several databases were searched including MEDLINE (via PubMed), China National Knowledge Infrastructure (via CNKI), Wanfang databases (via med.wanfangdata), China Science and Technology Journal Database (via cqvip), EMBASE (via Elsevier), and China Biology Medicine disc (via sinomed). All selected studies were published throughout April 10, 2021. We searched both controlled vocabulary and keywords in each database. No language restrictions were set. Detailed information about the research strategies is shown in Supplement Information S1.

Case‐control studies aimed at studying the association between SNPs and the risk of OC was involved. Patients with OC were confirmed by histopathological examination and all histopathological types were included. Populations who did not have OC were assigned to the control group. Therefore, ovarian cysts or other gynecological tumors were included in the control group. In addition, studies with an imbalance of Hardy–Weinberg equilibrium (HWE) in the control group, cases where the data were less comprehensive, and the insufficient gene phenotypes data were excluded. In addition, animal studies, case reports, reviews, academic exchanges, experience presentations, lectures, newsletters, etc. were excluded during the screening process. SNPs were included if they had been mentioned in at least two studies (two different targeted populations in one article were also included).

### 2.2 Screening method and data collection

**FIGURE 1:**
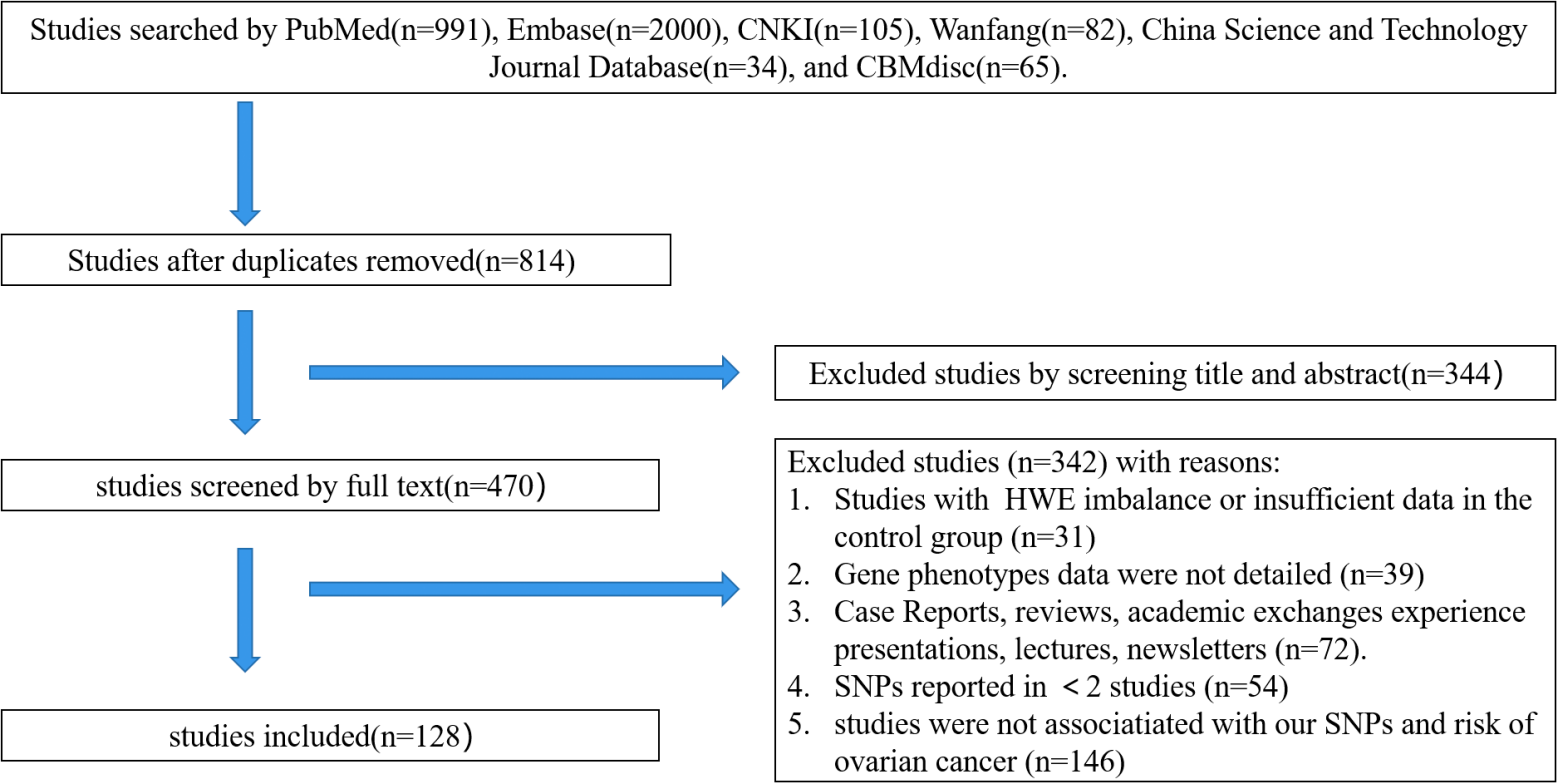
A PRISMA flow diagram

Two researchers (Jia Hu and Zhe Xu) independently decided whether the studies met the inclusion criteria. Both two phases of screening (title/abstract and full‐text) were conducted by them. If the results of the two researchers were inconsistent, a third researcher (Zhuomiao Ye) read title, abstract and full‐text carefully to decide whether studies should be included. A PRISMA flow diagram is shown in Figure 1. A total of 814 studies were included after the duplicated studies were removed. Only 128 studies were included after careful screening based on the inclusion and exclusion criteria. The following data were extracted from the studies: first author, year of publication, country, number of individuals in the case and control group, genotyping method, and HWE. In order to carry out subgroup analysis, we initially collected data on the histopathological type of OC and ethnicity of the studied population, but they were not mentioned in many studies. Therefore, we could not obtain complete data to conduct subgroup analysis.

### 2.3 Quality assessment

STREGA was applied to assess methodological quality of data. Two researchers screened independently, setting a score of 1 to meet the condition and 0 for failing to meet the condition. Assessment items included if genotyping methods were mentioned, whether to stratify the targeted population, whether to provide methods to infer genotypes, if the control group satisfied HWE, if the study could be repeated, if targeted population, inclusion, and exclusion criteria were described, whether to describe statistical methods and software, whether to provide methodologies of multiple comparisons, handling false positive findings or correcting relatedness, and whether the provided information was sufficient.

In addition, we used the ROBINS‐I tool to evaluate the risk of bias for all the included article from seven aspects.^20^ Specific items including (1) confounding, (2) selection of participants into the study, (3) classification of interventions, (4) intended intervention, (5) missing data, (6) measurement of outcomes, and (7) selection of the reported results. Two authors (Jin Li and Zhinan Hao) independently assessed the risk of bias. Any disagreement in the risk of bias score was resolved by Jia Hu. We assessed bias due to a confounding domain according to whether the control and case groups were matched by age, ethnicity and HWE. Because all studies were based on SNPs. Blood samples were obtained from participants in both case group and control group. The biases in selection of participants into the study, classification of intervention, deviations from intended interventions, and measurement of outcomes were "Low". We rated bias due to missing data by whether data were reported completely. Bias in selection of the reported result was evaluated from three domains including whether selectively report outcome, analytical method, and subgroups. Also, based on the results of the above evaluation, we could calculate the overall risk of bias and the results were reported in a rating of low, moderate, and serious.

### 2.4 Data analysis

Qualitative data were collected and pairwise meta‐analysis was used to compare the differences between the case and control groups. Pairwise meta‐analysis was undertaken using Stata software (version: 16, https://www.stata.com). Either the fixed effect model or random effect model was applied to meta‐analysis depending on heterogeneity between related studies. If *I* ^2^ > 0.1, the fixed effect model was used. Otherwise, a random effect model was applied. Six candidate gene models were set, including allele, homogeneous, heterozygous, dominant, recessive, and additive gene models. ORs with corresponding 95% confidence intervals (CIs) and *p*‐values were calculated. A *p* < 0.05 which generally represents the difference between two groups was considered statistically significant. Due to the limited targeted population and incomplete information, publication bias, sensitivity, and subgroup analyses were not evaluated.

To select the most appropriate gene model for OC, a network meta‐analysis was conducted by using R software (version 4.0.5; http://www.r‐project.org). The network diagram, ranking probabilities diagram, and inverted triangle diagram were drawn using R software. The most predictable gene model was selected by ranking probabilities and the algorithm established by Thakkinstian et al.^21^ Our implementation of Thakkinstian’s algorithm assumed that a recessive allele (a) mutant to a dominant allele (A). OR1, OR2, and OR3 represented AA versus aa, Aa versus aa, and AA versus Aa were calculated by paired meta‐analysis. Then the most appropriate gene model was selected by comparing the value of OR: If OR1 = OR3 ≠ 1 and OR2 = 1, we prioritized the recessive gene model. If OR1 = OR2 ≠ 1 and OR3 = 1, the dominant gene model was considered. If OR2 = 1/OR3 ≠1 and OR1 = 1, a complete over‐dominant model was chosen. If OR1 > OR2 > 1 along with OR1 > OR3 > 1 or OR1 < OR2 < 1 along with OR1 < OR3 < 1, the codominant gene model was selected. FPRP was also calculated by setting prior probability of 0.01 and an OR value of 1.5. Models with FPRP value <0.2 were considered credible. Besides, Venice criteria were used to assess cumulative epidemiologic evidence in genetic associations.^22^ Venice criteria were used to evaluate the credibility of evidence from three aspects, including the amount of evidence, extent of replication, and protection from bias. We finally generated a comprehensive evaluation of “strong”, “moderate”, or “weak” epidemiological credibility. Subgroup analysis was also conducted aimed at evaluating the effect of the most suitable gene model in modulating OC risk among ethnic subgroup.

Finally, a diagnostic meta‐analysis was conducted using Meta‐DiSc software (version: 1.4, http://www.hrc.es/investigacion/metadisc_en.htm).^23^ The pooled sensitivity, specificity, positive likelihood ratio (+LR), and negative likelihood ratio (−LR) were calculated. A summary receiver‐operating characteristic (SROC) curve and area under the curve (AUC) were used to comprehensively evaluate diagnostic accuracy. Spearman’s correlation coefficient was calculated to evaluate the correlation. In addition, the diagnostic odds ratio (DOR) was used to determine the direction of the relationship by analyzing all related studies. Due to the limited studies of the same SNP, bias of publication was not evaluated in our study.

## 3 RESULTS

### 3.1 Included studies

A total of 128 studies with 155,014 OC patients and 230,366 non‐cancer controls were included (Table 1). Initially, 74 genes and 108 SNPs were involved in the included studies. After careful screening based on the inclusion and exclusion criteria, only 63 genes and 92 SNPs met the selection criteria. Supplement Information S2 shows the data characteristics derived from the selected studies.

**TABLE 1:** Selected SNPs with corresponding articles

Gene	SNPs
APAI (C>A)	rs7975232^47^, ^48^
APAI (T>G)	rs7975232^49^
APE1	rs1130409,^50^, ^51^ rs1760944^50^, ^51^
BRCA1	rs799917,^9^, ^52^ rs1799950^9^
BRCA2	rs144848^53^
Bsm1 (C>A)	rs1544410^47^, ^54^
Bsm1 (C>T)	rs1544410^48^
Bsm1 (G>A)	rs1544410^49^, ^55^, ^56^
CDKN1B	rs2066827^8^, ^57^, ^58^
CDKN2A	rs3731249^8^, ^59^
Cdx‐2	rs11568820^47^, ^54^
COMT	rs4680^60^, ^61^, ^62^, ^63^, ^64^, ^65^, ^66^
COX‐2/PTGS2	rs20417,^67^ rs5275^67^, ^68^
CYP19A1	rs10046^53^
CYP1A2	rs762551^69^, ^70^
CYP1B1	rs1056827,^62^, ^64^ rs1056836,^61^, ^62^, ^63^, ^64^, ^71^ rs1800440^61^, ^62^
EPHX1	rs1051740^72^, ^73^, ^74^, ^75^, ^76^
ERCC1 (C>A)	rs3212986,^77^, ^78^, ^79^ rs2298881^77^, ^78^, ^79^
ERCC1 (C>T)	rs11615^77^, ^80^, ^81^
ERCC1 (G>A)	rs11615^78^, ^79^
ERCC2	rs238406,^82^, ^83^ rs13181,^58^, ^65^, ^84^, ^85^ rs1799793^65^, ^83^, ^84^, ^85^, ^86^
ESR1/SYNE1	rs2295190^87^
ESR2	rs3020450^88^, ^89^, ^90^
Fas	rs2234767^91^, ^92^
FasL	rs763110^63^, ^92^
Fok1	rs2228570^47^, ^49^, ^54^, ^55^, ^56^, ^93^, ^94^
FUT3	rs2306969^95^
GALNT1	rs17647532^95^, ^96^
GALNT2	rs2271077^97^
GALNT6	rs907352^95^
GALNT7	rs934358^95^
GSTP1	rs1695^85^, ^98^
H19	rs2107425^99^, ^100^
HER2	rs1136201^101^, ^102^, ^103^
HOTAIR	rs920778^104^
HSD17B1	rs605059^53^
HSD17B4	rs17145454^53^
IL‐10	rs1800871,^105^, ^106^ rs1800896^105^, ^107^
IL‐18	rs187238,^106^, ^108^ rs1834481^109^
LINC02354	rs7968585^48^
LncRNA‐HOTAIR	rs4759314^110^, ^111^
MCP‐1	rs1024611^112^, ^113^, ^114^, ^115^
MDM2	rs2279744,^116^, ^117^, ^118^ rs117039649^116^, ^119^
MGAT5	rs1257187^95^
miR‐146a	rs2910164^41^, ^120^, ^121^
miR‐196a2	rs11614913^19^, ^41^, ^120^, ^121^, ^122^
MLH1	rs1800734^123^, ^124^, ^125^, ^126^
MTHFR	rs1801133,^97^, ^127^, ^128^, ^129^, ^130^, ^131^, ^132^, ^133^, ^134^ rs1801131^97^, ^129^, ^132^, ^135^
MTR	rs1805087^129^, ^133^
MTRR	rs1801394^129^, ^133^
MUC16	rs2547065^125^, ^136^
NBS1	rs1063045,^9^ rs1805794,^9^ rs709816,^9^ rs1061302^9^
p16/CDKN2	rs11515,^137^, ^138^, ^139^
PGR	rs1042839,^140^, ^141^, ^142^, ^143^ rs1042838,^98^, ^140^, ^142^, ^143^, ^144^, ^145^, ^146^, ^147^ rs10895068^61^, ^65^, ^140^, ^141^, ^148^, ^149^, ^150^
PPAR‐γ/PPARG	rs1801282^67^, ^151^
RAD51	rs1801320,^9^, ^16^, ^132^, ^152^, ^153^, ^154^, ^155^ rs1801321^9^, ^16^
RAD52	rs11226^9^
RB1	rs4151551,^18^ rs3092904,^18^ rs4151636^18^
SRD5A2	rs523349^53^
ST3GAL3	rs3828139,^95^ rs37460^95^
STK15	rs2273535,^156^ rs1047972,^156^ rs732417,^156^ rs8173^156^
TaqI	rs731236^47^, ^48^, ^49^
TP53	rs1042522^98^, ^157^
VDR	rs2239179,^48^ rs3782905^48^
VEGF	rs3025039,^158^, ^159^, ^160^ rs833061^159^, ^161^
XRCC1	rs25487^85^, ^153^
XRCC2	rs3218536,^9^, ^53^, ^58^, ^155^, ^162^ rs718282^154^, ^163^, ^164^
XRCC3	rs861539,^9^, ^53^, ^100^, ^155^, ^162^, ^165^, ^166^ rs1799796,^9^ rs1799794^9^, ^165^

### 3.2 Quality assessment

The methodological quality evaluation results are shown in Supplement Information S3. The methodological quality score of all studies were equal to or greater than four, which means the methodology part of the included studies were of high quality. The evaluation results of risk of bias are plotted in Supplement Information S4. Through our screening, 120 studies were found to be of low risk, five studies of moderate risk, and no study of serious risk.

### 3.3 Pairwise meta‐analysis

A direct meta‐analysis was carried out to determine the association between 92 SNPs and OC risk (Supplement Information S5). *APE1 rs1130409* was significantly associated with a decreased risk of HCC under five types of gene models, except for the additive gene model. The GG and TG genotypes of *APE1 rs1130409* showed a negative correlation with OC risk compared to the TT genotype (GG + TG vs. TT: pooled OR = 0.525, 95% CI: 0.375–0.735). The TT genotype of XRCC2 rs718282 showed the remarkable correlation with increased OC risk compared to the CC and CT genotypes (TT vs. CC + CT: pooled OR = 3.211, 95% CI: 1.707–6.037). According to the fixed effects model, the AA genotype of ERCC1 rs3212986 was associated with increased OC risk compared to the CA and CC genotypes (AA vs. CA + CC: pooled OR = 1.636, 95% CI: 1.220–2.194). Based on the random effects model, the CC genotype of *RAD51 rs1801320* was associated with increased OC risk compared to the GG genotype (CC vs. GG: pooled OR = 2.545 95% CI: 1.265–5.118). In addition, the direct meta‐analysis results of other meaningful models are shown in Supplement Information S5.

### 3.4 Network meta‐analysis

**FIGURE 2:**
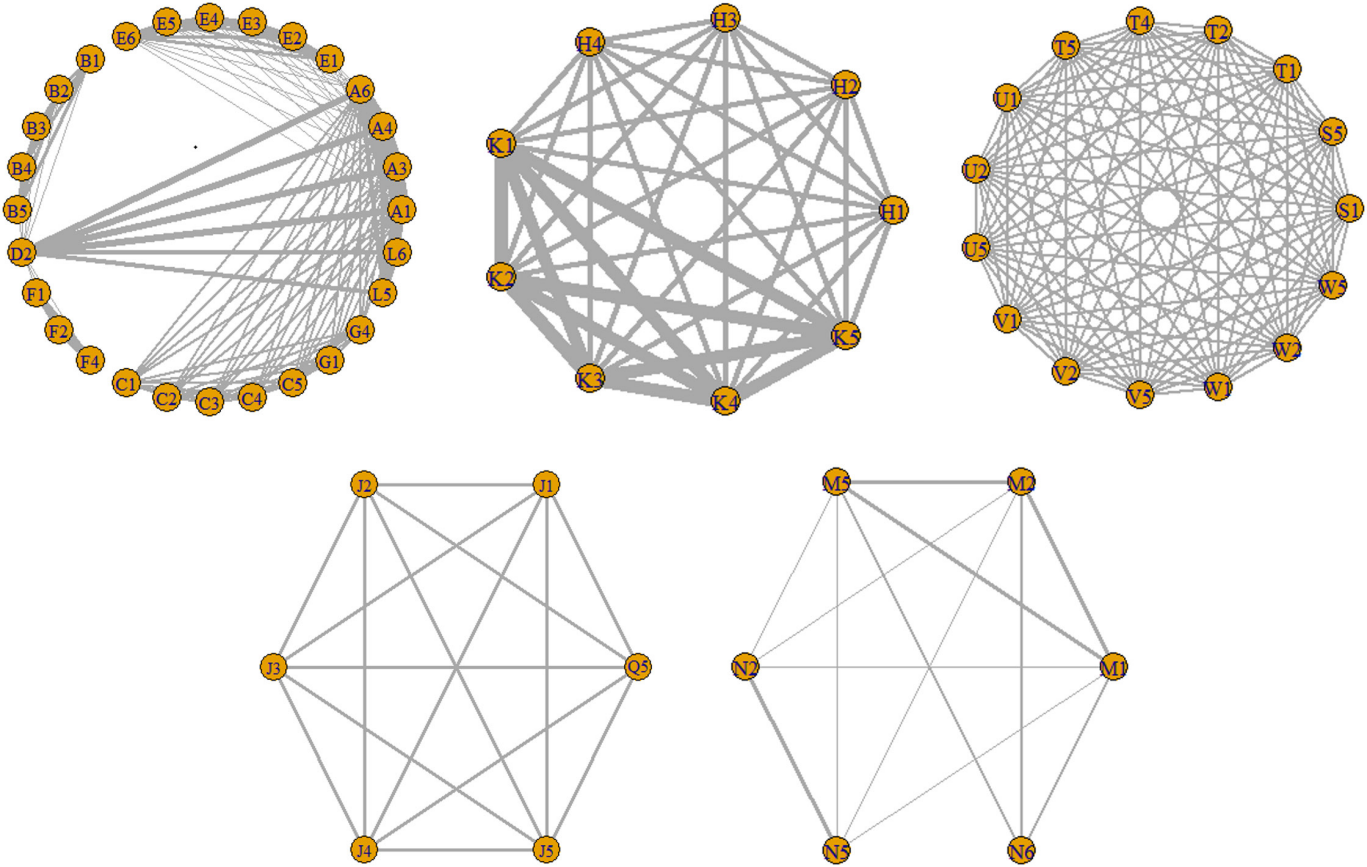
Network meta‐analysis results for the gene models of the OC risk‐related SNPs. The figure shows the network meta‐analysis results for the (1) Allele (2) Homozygous (3) Heterozygous (4) Dominant (5) Recessive and (6) Additive genetic models of the following SNPs: (A) XRCC3 rs861539; (B) XRCC2 rs718282; (C) SRD5A2 rs523349; (D) RAD51 rs1801320; (E) p16/CDKN2 rs11515; (F) MTHFR rs1801131; (G) BRCA2 rs144848; (H) Bsm1 rs1544410; (J) miR‐146a rs2910164; (K) Fok1 rs2228570; (L) XRCC3 rs1799796; (M) ERCC1 rs3212986; (N) ERCC1 rs11615; (Q) miR‐196a2 rs11614913; (S) GALNT6 rs907352; (T) GALNT7 rs934358; (U) MGAT5 rs1257187; (V) ST3GAL3rs3828139; (W) ST3GAL3 rs37460; (K) miR‐196a2 rs11614913. OC, ovarian cancer; SNPs, single nucleotide polymorphisms.

**FIGURE 3:**
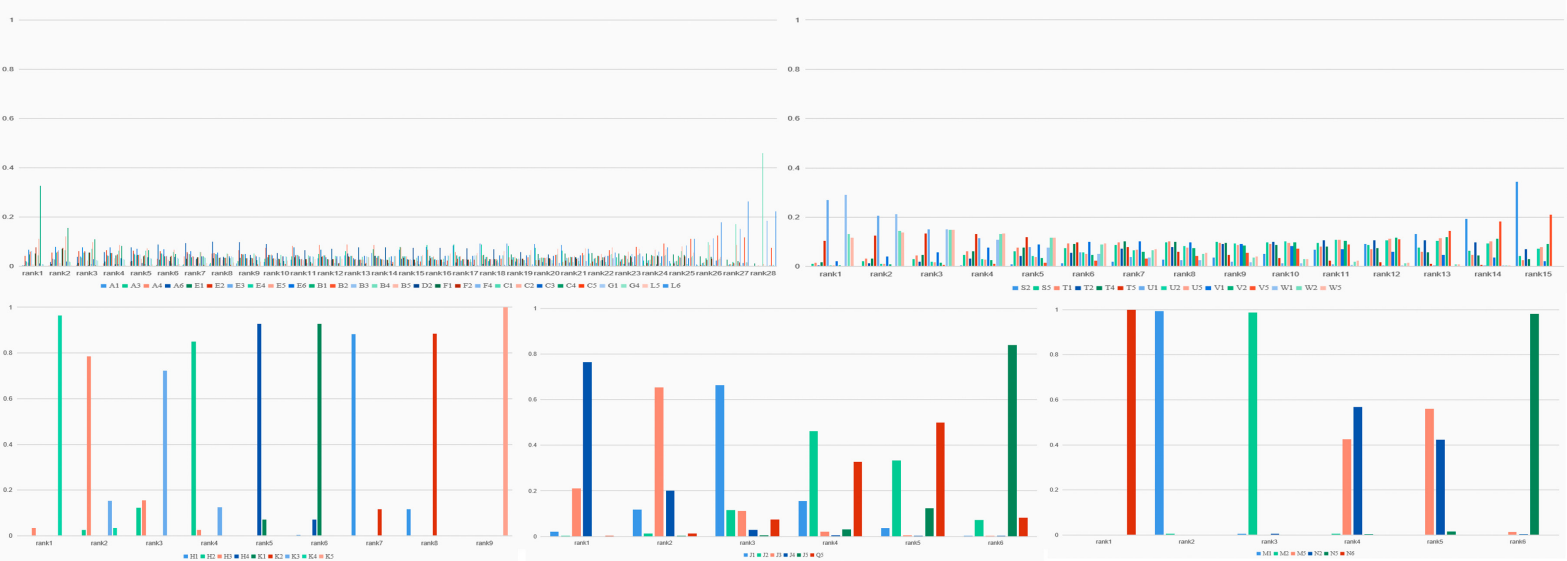
Rank probabilities for the five genetic models of the SNPs related to OC risk. The figure shows the network meta‐analysis results for the (1) Allele (2) Homozygous (3) Heterozygous (4) Dominant (5) Recessive and (6) Additive genetic models of the following SNPs: (A) XRCC3 rs861539; (B) XRCC2 rs718282; (C) SRD5A2 rs523349; (D) RAD51 rs1801320; (E) p16/CDKN2 rs11515; (F) MTHFR rs1801131; (G) BRCA2 rs144848; (H) Bsm1 rs1544410; (J) miR‐146a rs2910164; (K) Fok1 rs2228570; (L) XRCC3 rs1799796; (M) ERCC1 rs3212986; (N) ERCC1 rs11615; (Q) miR‐196a2 rs11614913; (S) GALNT6 rs907352; (T) GALNT7 rs934358; (U) MGAT5 rs1257187; (V) ST3GAL3 rs3828139; (W) ST3GAL3 rs37460; (K) miR‐196a2 rs11614913; *Note*: Genetic model of an SNP with best mean probability is considered the optimal gene model. OC, ovarian cancer; SNPs, single nucleotide polymorphisms.

The network meta‐analysis was further carried out by using a consistency model to compare the gene models of different SNPs which demonstrated a significant correlating with OC risk in the direct meta‐analysis. Figure 2 suggests that some SNPs are connected to a network. There are altogether five networks, and they consist of different SNPs. After comparing the gene models by paired meta‐analysis (Supplement Information S6) and network meta‐analysis. Five SNPs (SRD5A2 rs523349, Fok1 rs2228570, ST3GAL3 rs37460, miR‐146a rs2910164, and ERCC1 rs11615) with corresponding gene models were selected according to the probability of ranking (Figure 3).

### 3.5 Thakkinstian’s analysis and credibility evaluation

We found that the codominant model was the most suitable for these five SNPs based on Thakkinstian’s analysis (Table 2). The FPRP values of these five SNPs with the aforementioned gene models were calculated (Table 2). The dominant gene model and homogeneous gene model of SRD5A2 rs523349, the dominant gene model and homogeneous gene model of Fok1 rs2228570 and homogeneous gene model of miR‐146a rs2910164 showed a lower FPRP <0.2. The codominant gene model was replaced by the results of homozygous gene model and heterozygous gene model. Though the FPRP of SRD5A2 rs523349 and Fok1 rs2228570 homozygous gene model were <0.2. The FPRP of heterozygous gene model of these two SNPs were >0.2. Thus, the codominant gene models of these two SNPs were not the most appropriate model to predict OC risk. Besides, we reviewed articles containing these SNPs and found that SRD5A2 rs523349 and miR‐146a rs2910164 only were mentioned in two and three OC‐related studies, respectively. While Fok1 rs2228570 was explored in 16 studies. Therefore, Fok1 rs2228570 was found to be the most appropriate gene for predicting OC based on existing studies. The FPRP value of the dominant gene model of Fok1 rs2228570 was 0.038. Therefore, the dominant gene model of Fok1 rs2228570 was the most suitable gene model.

**TABLE 2:** Gene model selected by Thakkinstian’s algorithm

Gene	Gene model	OR (95% CI)	Thakkinstian’s algorithm results	FPRP of 0.01 prior probability^a^
SRD5A2 rs523349				
CC + GC versus GG^b^ ^,^ ^c^		1.305 (1.132–1.504)		0.024
CC versus GC^c^	D3	1.479 (1.170–1.868)		0.156
GC versus GG	D2	1.196 (1.028–1.391)	Codominant model	0.667
CC versus GG^c^	D1	1.756 (1.393–2.213)		0.002
Fok1 rs2228570				
TT + CT versus CC^b^ ^,^ ^c^		1.158 (1.068–1.256)		0.038
TT versus CT	D3	1.080 (0.964–1.210)		0.948
CT versus CC	D2	1.138 (1.044–1.240)	Codominant model	0.238
TT versus CC^c^	D1	1.224 (1.087–1.378)		0.076
ST3GAL3 rs37460				
C versus G^b^		0.853 (0.747–0.974)		0.651
CC versus GC	D3	0.766 (0.603–0.973)		0.767
GC versus GG	D2	0.938 (0.751–1.172)	Codominant model	0.983
CC versus GG	D1	0.719 (0.550–0.940)		0.688
miR‐146a rs2910164				
CC + GC versus GG^b^		0.293 (0.121–0.709)		0.949
CC versus GC	D3	0.701 (0.470–1.045)		0.931
GC versus GG	D2	0.329 (0.126–0.860)	Codominant model	0.969
CC versus GG	D1	0.193 (0.114–0.325)		0.037
ERCC1 rs11615				
GG + AA versus GA^b^		1.696 (1.024–2.810)		0.926
AA versus GA	D3	1.433 (0.766–2.679)		0.979
GA versus GG	D2	0.571 (0.315–1.035)	Codominant model	0.955
AA versus GG	D1	0.819 (0.454–1.476)		0.985

*Note*: D1, D2, D3 were defined by Thakkinstian’s algorithm. D1: homozygous gene model; D2: heterozygous gene model; D3: mutant homozygote versus heterozygote.

Abbreviations: CI, confidence interval; FPRP, false positive report probability.

^a^ FPRP was used to detect the gene models obtained through the network meta‐analysis and Thakkinstian’s algorithm, respectively. The codominant gene model was replaced by the results of homozygous gene model and heterozygous gene model.

^b^ This gene model is the most suitable gene model selected by the network meta‐analysis.

^c^ The gene models with FPRP <0.2 when the prior probability was 0.01.

Venice criteria was further used to assess cumulative epidemiologic evidence (Supplement Information S7). The relationship between ERCC1 rs11615, miR‐146a rs2910164 and OC risk results in weak evidence due to the small amount of the smallest genetic group. The credibility of the relationship between SRD5A2 rs523349, Fok1 rs2228570 and ST3GAL3 rs37460 were moderate due to the combination of BAB (amount of evidence as B, replication as A and protection from bias as B). Though SRD5A2 rs523349 and ST3GAL3 rs37460 showed a moderate credibility, they were only mentioned in two OC‐related studies, which were not enough to prove having a genetic connection with OC compared with Fok1 rs2228570. In short, combining network meta‐analysis, Thakkinstian’s analysis, FPRP and Venice criteria, the dominant gene model of Fok1 rs2228570 was the most suitable gene model for predicting OC risk with a moderate credibility.

**FIGURE 4:**
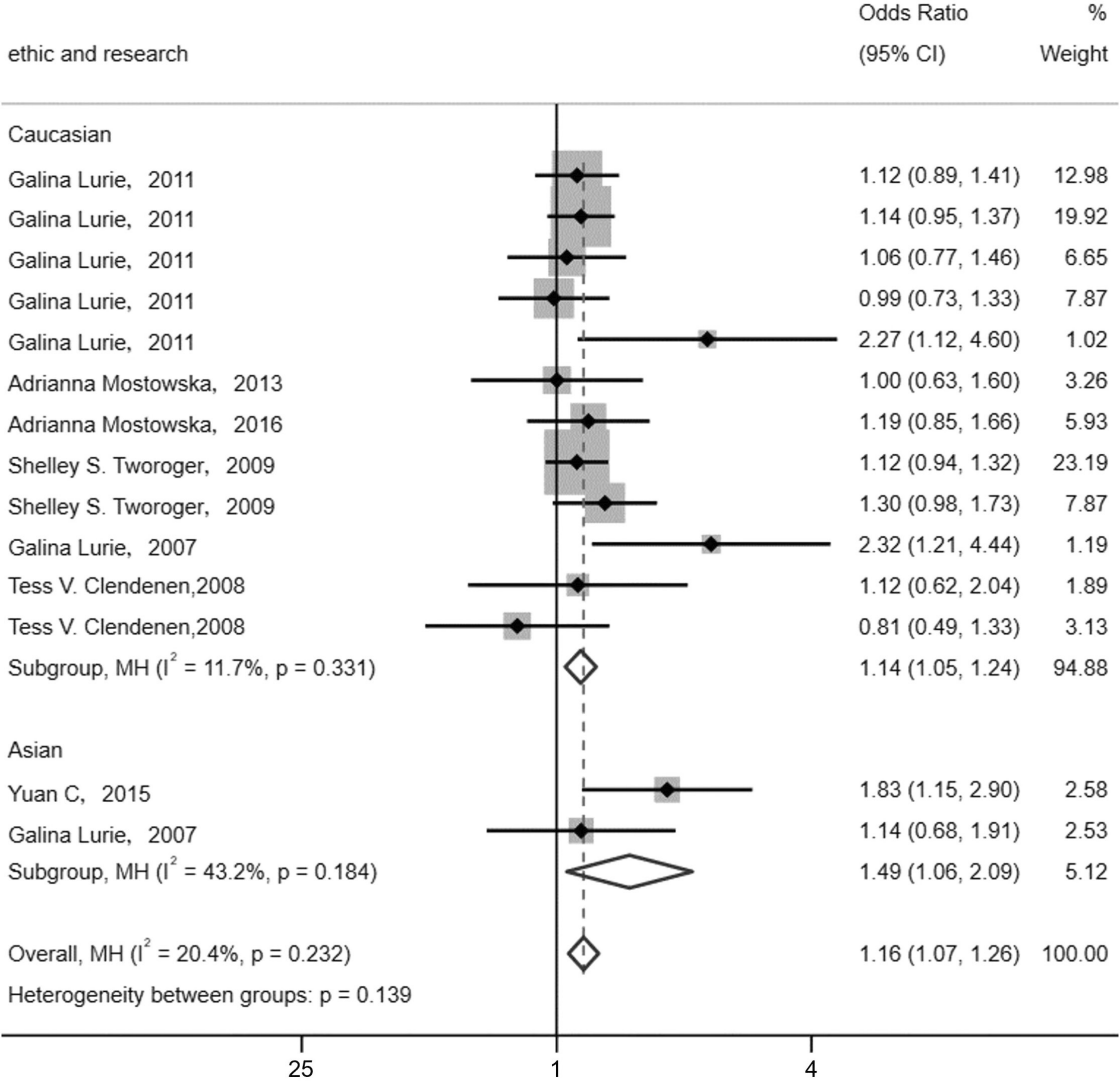
Subgroup analysis of the dominant gene model of Fok1 rs2228570 in Caucasian and Asian groups.

The researches about Fok1 rs2228570 carried on Caucasian and Asian groups. Other gene models were based on Caucasian. So, subgroup analysis was further performed in dominant gene model of Fok1 rs2228570. It showed that the odd ratio of Caucasian and Asian were 1.14 (95% CI, 1.05–1.24) and 1.49 (95% CI, 1.06–2.09), respectively (Figure 4). In other words, the dominant gene model of Fok1 rs2228570 was associated with OC in Caucasian and Asian population.

**FIGURE 5:**
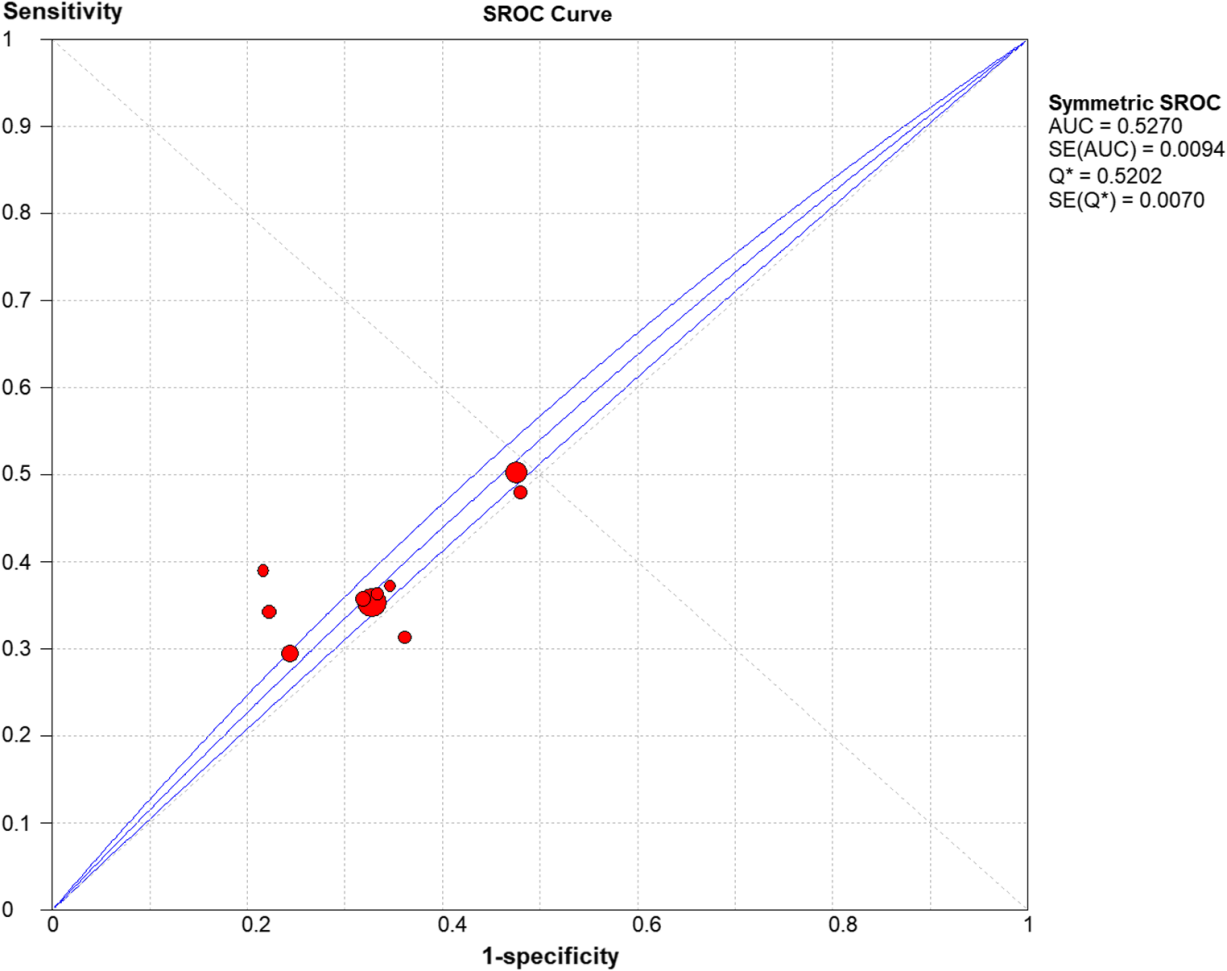
SROC curve of Fok1 rs2228570 identifying OC under the dominant gene model. OC, ovarian cancer; SROC, summary receiver‐operating characteristic.

Finally, a diagnostic meta‐analysis was performed to estimate the diagnostic power of the dominant gene model of Fok1 rs2228570 for OC. The results showed that the pooled sensitivity, specificity, LR+, and LR− of the dominant gene model of Fok1 rs2228570 were 0.383 (95% CI, 0.371–0.395), 0.651 (95% CI, 0.636–0.666), 1.100 (95% CI, 1.024–1.182), and 0.940 (95% CI, 0.906–0.975), respectively. The SROC curve was obtained by combining the specificity and sensitivity (Figure 5). For Fok1 rs2228570 with the dominant gene model, the AUC and Q‐value of the SROC curve were 0.5270 and 0.5202, respectively. The DOR and Spearman correlation coefficients were 1.176 (95% CI, 1.053–1.313) and 0.43 (*p* = 0.214), respectively.

## 4 DISCUSSION

OC accounts for only 1.6% of all cancers with a 2.1% cause of cancer‐related mortality (207,252 deaths) in 2020 worldwide.^1^ An increasing number of scientific studies have indicated that SNPs might be used as a possible marker for predicting OC risk. However, there is no study aimed at studying all reported SNPs associated with OC risk. To the best of our knowledge, this is one of the first comprehensive systematic review and network meta‐analysis to explore the most appropriate SNP with its corresponding gene model for predicting OC risk.

Our study not only aimed to study the association between SNPs and OC, but also aimed to select the most appropriate gene model for predicting OC risk. We did not hypothesize generic models, because inappropriate assumptions of underlying genetic model may lead to a misleading result. Given that, in this study, no assumption was made. Six general gene models (allele, homogeneous, heterozygous, dominant, recessive, and additive gene models) were evaluated in our study. To identify the most appropriate model for OC risk association, both network meta‐analysis and Thakkinstian’s algorithm were performed. Network meta‐analysis is based on direct comparison of pairwise meta‐analysis and adds indirect comparison to the study. The ranking probability was used to select the most appropriate model with a Bayesian approach based on the network meta‐analysis. Combining ranking probability and Thakkinstian analysis improved the reliability of the results. In our study, SRD5A2 rs523349, Fok1 rs2228570, ST3GAL3 rs37460, miR‐146a rs2910164, and ERCC1 rs11615 with corresponding gene models were selected according to the probability of ranking.

Then, Thakkinstian’s analysis was performed in these five selected SNPs. Thakkinstian’s algorithm has the advantage of not assuming a priori gene model. This approach compares OR of homozygous gene model, heterozygous gene model, and mutant homozygote versus heterozygote model to generate the most appreciate gene model. After comparing the OR values of different gene model, the codominance model was the most suitable gene model for all five SNPs to predict OC risk. However, the codominance model was not considered in the network meta‐analysis. Therefore, the homozygous and heterozygous gene models were used to represent the codominant gene model. The FPRP analysis was used to assess significant associations. We set a prior probability of 0.01, an OR of 1.50, and a FPRP cutoff value of 0.20 to select the most suitable gene model for predicting OC risk.^24^, ^25^, ^26^ Only a significant result with a FPRP <0.20 was considered credible. Our results demonstrated that Fok1 rs2228570 was more strongly associated with OC risk than SRD5A2 rs523349, ST3GAL3 rs37460, miR‐146a rs2910164, and ERCC1 rs11615. Although Fok1 rs2228570 and SRD5A2 rs523349 were significantly associated with OC risk with FPRP values <0.2, only two studies mentioned by one article aimed at studying the association between SRD5A2 rs523349 and OC. Therefore, Fok1 rs2228570 may be the most appropriate SNP for predicting OC risk. Subgroup analysis illustrated that the dominant gene model of Fok1 rs2228570 had a significant association with OC risk in Caucasian and Asian population. But we noted that there were only two studies aimed at Asian population. Therefore, the relevance between Fok1 rs2228570 and OC risk in the Asian population needs to be further explored. More studies should be carried out to explore the association between SNPs with the risk of OC.

Finally, a diagnostic meta‐analysis was performed to evaluate the diagnostic power. We found that the specificity of Fok1 rs2228570 for predicting OC risk was 0.651 (95% CI, 0.636–0.666), while the sensitivity was only 0.383 (95% CI, 0.371–0.395). The results demonstrated that predicting OC risk merely depending on Fok1 rs2228570 would have a higher incidence of missed diagnosis. In addition, the AUC of Fok1 rs2228570 for predicting OC risk was only 0.5270, which was far from the optimal value of the AUC. Therefore, Fok1 rs2228570 alone was not sufficient for screening OC, but might be used as an auxiliary screening tool to improve the diagnostic power of OC. In addition, combining two or more SNPs to predict OC may have a synergistic outcome and could merit further study.

Notably, we found Fok1 rs2228570 under dominant gene model was significantly associated with OC risk and the genetic association was credible according to FPRP analysis and Venice criteria. Fok1 is one of the vitamin D receptor (VDR), which is a typical nuclear receptor.^27^ Vitamin D plays critical roles in several biological processes including immunity, cell proliferation, differentiation, migration, death, and apoptosis.^28^, ^29^ After being metabolized by the liver and kidneys, vitamin D transforms into 1, 25‐dihydroxyvitamin D3, which is the active form of vitamin D, and exerts its effects by combining with the VDR. VDR and cancer risk have recently been the focus of a number of studies. SNPs of VDR are not only associated with incidence risk or prognosis of cancer, but are also associated with other diseases, such as nonalcoholic fatty liver disease.^30^ and coronary artery disease.^31^ The association between vitamin D and OC risk remains controversial. Two recent studies showed a protective role of vitamin D in OC by Mendelian randomization analyses.^32^, ^33^ Our results are consistent with those of recent studies. Fok1 rs2228570 under the dominant gene model was significantly associated with OC risk with a FPRP of 0.038. In other words, a significantly increased ovarian risk was found among both TT and CT mutation genotypes compared to the wild‐type CC genotype of Fok1 rs2228570. Part of the explanation is that the mutation of Fok1 changes the structure and function of Fok1. Thus, the vitamin D signaling pathway was prohibited and could not play a protective role in OC incidence. Raza et al. showed that SNPs of Fok1 are associated with breast cancer susceptibility by regulating genes such as hp21 and hFOXO1, which are involved in proliferation, differentiation, migration, death, and apoptosis.^34^ Therefore, Fok1 may play a role in modulating OC risk by regulating genes that are involved in the occurrence and progression of cancers. Moreover, we also speculate that mutation affect the function of VDR. The mutant T allele of the Fok1 rs2228570 located in the coding region of the VDR gene, participating in protein transcription and translation. The mutation from C to T results in the production of a longer VDR protein, which means less responsive to 1, 25‐dihydroxyvitamin D3.^35^ This may contribute to reduce immunity response, influencing cell proliferation, differentiation, migration and apoptosis and potentially lead to tumorigenesis.

Four other selected SNPs determined from network meta‐analysis are also worthy of note. Steroid 5‐alpha‐reductase type 2 (SRD5A2) is a steroid hormone‐metabolizing enzyme, which involves in the conversion process from testosterone to dihydrotestosterone. Dihydrotestosterone is a kind of androgen, so SRD5A2 is responsible for sexual development in males.^36^, ^37^ Our results showed that SRD5A2 rs523349 variant may increase the risk of OC. The mechanism of these association remains unclear. We assumed SNPs variation of SRD5A2 may affect enzyme activity. It had been reported that “C” variant of the SRD5A2 rs523349 may lead to a decreased enzyme activity in OC.^38^ SRD5A2 protein serves as a catalyst for generating dihydrotestosterone. Thus, lower level of dihydrotestosterone may serve as a risk factor of OC. Till date, there is no study exploring the impact of dihydrotestosterone activity on OC risk. SRD5A2 rs523349 variation and dihydrotestosterone activity are new ways to study OC. This analysis was based on two Australian studies that were not representative enough. Therefore, our results require confirmation by further studies and the mechanisms of the association need to be investigated.

Sialyltransferases, play a catalytic role in adding sialic acid to glycolipids or glycoproteins, and are involved in neoplastic transformation and progression.^39^ 2,3‐sialyltransferase (ST3GAL3), as a kind of Sialyltransferases, has been found to be deregulated in OC.^40^ A previous study showed that knockdown of ST3GAL3 led to a significant increase in cell migration and invasion in pancreatic ductal adenocarcinoma. It is interesting to note that ST3GAL3 rs37460 variant led to a decreased risk of OC. However, to the best of our knowledge, the specific mechanism of the ST3GAL3 rs37460 variant and the risk of OC has received little attention. It follows from the above that ST3GAL3 rs37460 variant results in decreased expression of ST3GAL3 and influences the formation and progression of OC.

MicroRNAs (miRNAs) as gene expression regulators are involved in biological processes by inhibiting and destabilizing the target RNAs. The targets RNAs of MicroRNA‐164a (miR‐146a) include tumor necrosis factor, interleukin 1‐beta, tumornecrosis factor receptor‐associated factor 6, and so on.^41^ Our results showed that the miR‐146a rs2910164 polymorphism led to a decreased risk of OC. One possible reason is that miR‐146a regulates the expression of inflammation cytokines and mediates immune system disorders. It is known that immune microenvironment plays a critical role in tumorigenesis. It is reported that miRNA polymorphisms could affect the expression levels of tumor suppressor genes or oncogenes which are related to cancer risk. We speculated that miR‐146a rs2910164 exerts its role by regulating Toll‐like receptors and cytokine signaling, which is crucial process for oncogenesis.^42^

ERCC1, one of the genes involved in the nucleotide excision repair pathway, plays a key role in maintaining genomic stability and preventing tumor development. The association between ERCC1 rs11615 and cancer risk differs according to the cancer type. Previous studies have shown that the ERCC1 rs11615 variant is associated with breast cancer and pancreatic cancer risk.^43^, ^44^ However, the ERCC1 rs11615 variant was not associated with oral squamous cell carcinoma in Chinese patients.^45^ The study by Yong‐Jun Ma et al. demonstrated significant association between the homozygote and recessive models of ERCC1 rs11615 C>T and OC risk.^46^ Our results showed that there was significant association between the dominant model of ERCC1 rs11615 G>A polymorphism and OC risk. These different results illustrated that different base mutation of the same gene may lead to different biological process. We assumed that different base mutation led to increasing or decreasing expression of ERCC1, thus led to a reduced or increased OC risk. Therefore, more comprehensive studies with larger targeted population should be performed to verify the relationship between significant genetic variations in the ERCC1 and OC risk due to the limited studies our research based on.

Our meta‐analysis is one of the first to comprehensively cover all OC risk‐related SNPs mentioned in previously published studies in this field. Previous meta‐analyses on this topic only included several genes with similar functions or only studied genes without SNPs and different gene models. By contrast, we analyzed 64 genes and 94 SNPs with six gene models, which may be able to provide a more precise guide for clinical practice. Moreover, for each result with statistical significance, meta‐analysis and Thakkinstian’s algorithm analysis were undertaken, and FPRP was calculated to further verify the reliability of the results.

Our meta‐analysis had several limitations. First, we selected SNPs with two or more studies to perform direct and network meta‐analysis, but some studies have relatively small‐targeted populations and the representativeness of these studies are thereby poor. Therefore, caution should be taken when interpreting the results. Second, due to insufficient information, some factors that may influence the results were neglected. Third, the data quality of some studies was poor based on the STREGA score classification and the credibility of results would decrease. Fourth, because this meta‐analysis was based on mass of literatures and screening was conducted by researchers instead of screening tool or software. There may be some omissions in the selected SNPs for meta‐analysis. Hence, further analysis with large samples and high quality research could help confirm our findings.

Our meta‐analysis verified that the dominant gene model of Fok1 rs2228570 might be used as an indicator of OC based on previous studies. Hence, further studies with larger sample sizes, higher quality, and more detailed information are necessary to substantiate the implications of our results. Studies exploring the undesirable synergistic effect of genetic and other risk factors to predict OC risk present another avenue of research warranting further attention in the future.

## AUTHOR CONTRIBUTIONS

Jia Hu and Yongjun Wang participated in the design of this study. Jia Hu, Zhe Xu, and Zhuomiao Ye searched and screened literatures. Jin Li and Zhinan Hao assessed methodological quality of data. Pairwise meta‐analysis was conducted by Jia Hu and Zhe Xu. Network meta‐analysis were conducted Jia Hu, Zhuomiao Ye, and Jin Li. Venice criteria and diagnostic meta‐analysis were performed by Zhinan Hao. Finally, Jia Hu, Zhe Xu, Zhuomiao Ye, Jin Li, and Zhinan Hao wrote article together and Yongjun Wang revised the manuscript.

## CONFLICT OF INTEREST

The authors declare that the research was conducted in the absence of any commercial or financial relationships that could be construed as a potential conflict of interest.

## ETHICAL APPROVAL STATEMENT

Animals were not used in our research.

## Supporting information


Supplement Information S1

Supplement Information S2

Supplement Information S3

Supplement Information S4

Supplement Information S5

Supplement Information S6

Supplement Information S7

## Data Availability

The data that support the findings of this study are available in the previously published studies, which have been cited in the reference part.
