# Supplementary material for: The association between single nucleotide polymorphisms and ovarian cancer risk: A systematic review and network meta‐analysis
Source: Cancer Med. 2022 May 30;12(1):541–56. doi: 10.1002/cam4.4891 (PMC9844622; doi:10.1002/cam4.4891)
Supplement: Supplementary file 1 — Supplement Information S1 [file CAM4-12-541-s007.pdf]

Supplement information 1. Search strategy.

Pubmed (via Pubmed)

((((((((((((((((((((((Ovarian Neoplasms[MeSH Terms]) OR (Neoplasm, Ovarian[Title/Abstract])) OR (Ovarian Neoplasm[Title/Abstract])) OR (Ovary Neoplasms[Title/Abstract])) OR (Neoplasm, Ovary[Title/Abstract])) OR (Neoplasms, Ovary[Title/Abstract])) OR (Ovary Neoplasm[Title/Abstract])) OR (Neoplasms, Ovarian[Title/Abstract])) OR (Ovary Cancer[Title/Abstract])) OR (Cancer, Ovary[Title/Abstract])) OR (Cancers, Ovary[Title/Abstract])) OR (Ovary Cancers[Title/Abstract])) OR (Ovarian Cancer[Title/Abstract])) OR (Cancer, Ovarian[Title/Abstract])) OR (Cancers, Ovarian[Title/Abstract])) OR (Ovarian Cancers[Title/Abstract])) OR (Cancer of Ovary[Title/Abstract])) OR (Cancer of the Ovary[Title/Abstract])) OR ((((((((((((((((((((((((Carcinoma, Ovarian Epithelial[MeSH Terms]) OR (Carcinomas, Ovarian Epithelial[Title/Abstract])) OR (Epithelial Carcinoma, Ovarian[Title/Abstract])) OR (Epithelial Carcinomas, Ovarian[Title/Abstract])) OR (Ovarian Epithelial Carcinomas[Title/Abstract])) OR (Epithelial Ovarian Cancer[Title/Abstract])) OR (Ovarian Epithelial Cancer[Title/Abstract])) OR (Cancer, Ovarian Epithelial[Title/Abstract])) OR (Cancers, Ovarian Epithelial[Title/Abstract])) OR (Epithelial Cancer, Ovarian[Title/Abstract])) OR (Epithelial Cancers, Ovarian[Title/Abstract])) OR (Ovarian Epithelial Cancers[Title/Abstract])) OR (Ovarian Cancer, Epithelial[Title/Abstract])) OR (Cancer, Epithelial Ovarian[Title/Abstract])) OR (Cancer, Epithelial Ovarian[Title/Abstract])) OR (Epithelial Ovarian

Cancers[Title/Abstract])) OR (Ovarian Cancers, Epithelial[Title/Abstract])) OR (Ovarian Epithelial Carcinoma[Title/Abstract])) OR (Epithelial Ovarian Carcinoma[Title/Abstract])) OR (Carcinoma, Epithelial Ovarian[Title/Abstract])) OR (Carcinomas, Epithelial Ovarian[Title/Abstract])) OR (Epithelial Ovarian Carcinomas[Title/Abstract])) OR (Ovarian Carcinoma, Epithelial[Title/Abstract])) OR (Ovarian Carcinomas, Epithelial[Title/Abstract]))) OR ((((serous ovarian cancer[Title/Abstract]) OR (Mucinous ovarian cancer[Title/Abstract])) OR (Teratoma, Ovarian[MeSH Terms])) OR (Dermoid Cyst, Ovarian[MeSH Terms]))) OR ((((((((((((((((((((((ovarian germinoma[Title/Abstract]) AND (Teratoma[MeSH Terms])) OR (Teratomas[Title/Abstract])) OR (Dysembryoma[Title/Abstract])) OR (Dysembryomas[Title/Abstract])) OR (Teratoid Tumor[Title/Abstract])) OR (Teratoid Tumors[Title/Abstract])) OR (Tumor, Teratoid[Title/Abstract])) OR (Tumors, Teratoid[Title/Abstract])) OR (Teratoma, Benign[Title/Abstract])) OR (Benign Teratoma[Title/Abstract])) OR (Benign Teratomas[Title/Abstract])) OR (Teratomas, Benign[Title/Abstract])) OR (Teratoma, Mature[Title/Abstract])) OR (Teratoma, Cystic[Title/Abstract])) OR (Teratoma, Malignant[Title/Abstract])) OR (Malignant Teratoma[Title/Abstract])) OR (Malignant Teratomas[Title/Abstract])) OR (Teratomas, Malignant[Title/Abstract])) OR (Teratoma, Immature[Title/Abstract])) OR (Immature Teratoma[Title/Abstract])) OR (Immature Teratomas[Title/Abstract])) OR (Teratomas, Immature[Title/Abstract]))) OR (((((Granulosa cell tumor of the ovary[MeSH Terms]) OR (Granulosa theca cell tumor of the ovary[Title/Abstract])) OR (Adult granulosa cell tumor of the ovary[Title/Abstract])) OR (GCT of the ovary[Title/Abstract])) OR

(Granulosa theca cell tumor[Title/Abstract]))) AND ((((((((Polymorphism, Single Nucleotide[MeSH Terms]) OR (Nucleotide Polymorphism, Single[Title/Abstract])) OR (Nucleotide Polymorphisms, Single[Title/Abstract])) OR (Polymorphisms, Single Nucleotide[Title/Abstract])) OR (Single Nucleotide Polymorphisms[Title/Abstract])) OR (SNPs[Title/Abstract])) OR (Single Nucleotide Polymorphism[Title/Abstract])))

Embase (via Elsevier)

No.	Query Results	Results
#11.	#9 AND #10	1,217
#10.	#7 OR #8	141,518
#9.	#1 OR #2 OR #3 OR #4 OR #5 OR #6	194,226
#8.	'nucleotide polymorphisms, single':ab,ti OR 'polymorphisms, single nucleotide':ab,ti OR 'nucleotide polymorphism, single':ab,ti OR 'polymorphism, single nucleotide':ab,ti OR snps:ab,ti OR 'single nucleotide polymorphism':ab,ti OR 'single nucleotide polymorphisms':ab,ti	141,516
#7.	'single nucleotide polymorphisms'/exp	15
#6.	'ovarian stromal cell tumor':ab,ti OR 'granulosa theca cell tumor':ab,ti OR 'gct of the ovary,':ab,ti OR 'granulosa theca cell tumor of	418

	the ovary':ab,ti OR 'adult granulosa cell tumor of the ovary':ab,ti OR 'granulosa cell tumor of the ovary':ab,ti	
#5.	'epithelial ovarian cancers':ab,ti OR 'epithelial ovarian carcinoma':ab,ti OR 'carcinomas, ovarian epithelial':ab,ti OR 'epithelial carcinomas, ovarian':ab,ti OR 'epithelial ovarian carcinomas':ab,ti OR 'carcinoma, ovarian epithelial':ab,ti OR 'ovarian epithelial carcinomas':ab,ti OR 'cancers, epithelial ovarian':ab,ti OR 'epithelial carcinoma, ovarian':ab,ti OR 'epithelial cancer, ovarian':ab,ti OR 'ovarian cancers, epithelial':ab,ti OR 'ovary carcinoma':ab,ti OR 'cancers, ovarian epithelial':ab,ti OR 'epithelial ovarian cancer':ab,ti OR 'epithelial cancers, ovarian':ab,ti OR 'cancer, epithelial ovarian':ab,ti OR 'ovarian epithelial cancers':ab,ti OR 'ovarian cancer, epithelial':ab,ti OR 'cancer, ovarian epithelial':ab,ti OR 'carcinomas, epithelial ovarian':ab,ti OR 'ovarian carcinoma,	18,217

	epithelial':ab,ti OR 'ovarian carcinomas, epithelial':ab,ti OR 'ovarian epithelial carcinoma':ab,ti OR 'ovarian epithelialcarcinoma':ab,ti	
#4.	'serous ovarian cancer':ab,ti OR 'mucinous ovarian cancer':ab,ti OR 'endometrioid ovarian cancer':ab,ti OR 'ovarian germinoma':ab,ti OR teratoma:ab,ti OR teratomas:ab,ti OR dysembryomas:ab,ti OR 'teratoid tumors':ab,ti OR 'teratoid tumor':ab,ti OR dysembryoma:ab,ti OR 'tumor, teratoid':ab,ti OR 'teratoma, benign':ab,ti OR 'benign teratomas':ab,ti OR 'tumors, teratoid':ab,ti OR 'benign teratoma':ab,ti OR 'teratomas, benign':ab,ti OR 'teratoma, mature':ab,ti OR 'teratoma, malignant':ab,ti OR 'teratoma, cystic':ab,ti OR 'malignant teratomas':ab,ti OR 'teratomas, malignant':ab,ti OR 'malignant teratoma':ab,ti OR 'teratomas, immature':ab,ti OR 'immature teratomas':ab,ti OR 'immature teratoma':ab,ti OR 'teratoma, immature':ab,ti	24,877
#3.	'ovary carcinoma'/exp	38,456

#2.	'ovarian neoplasms':ab,ti OR 'neoplasm, ovarian':ab,ti OR 'ovarian neoplasm':ab,ti OR 'ovary neoplasms':ab,ti OR 'neoplasm, ovary':ab,ti OR 'neoplasms, ovary':ab,ti OR 'ovary neoplasm':ab,ti OR 'cancers, ovary':ab,ti OR 'cancer, ovary':ab,ti OR 'neoplasms, ovarian':ab,ti OR 'ovary cancer':ab,ti OR 'cancers, ovarian':ab,ti OR 'ovary cancers':ab,ti OR 'cancer, ovarian':ab,ti OR 'ovarian cancer':ab,ti OR 'ovarian cancers':ab,ti OR 'cancer of ovary':ab,ti OR 'cancer of the ovary':ab,ti	94,848
#1.	'ovary tumor'/exp	167,382

China National Knowledge Infrastructure (via CNKI)

SU= (‘ovarian carcinomas’+ ‘ovarian epithelial carcinomas’ + ‘epithelial ovarian carcinomas’ + ‘ovarian germinoma’ + ‘teratoma’ + ‘mucinous ovarian cancer’ + ‘serous ovarian cancer’ + ‘endometrioid ovarian cancer’ + ‘ovarian stromal cell tumor’ + ‘’) granulosa cell tumor of the ovary * ‘single nucleotide polymorphisms’

Wanfang databases (via med.wanfangdata)

Topic: (("ovarian carcinomas" or "ovarian epithelial carcinomas" or "epithelial

ovarian carcinomas" or "ovarian germinoma" or "teratoma" or "mucinous ovarian cancer" or "serous ovarian cancer" or "endometrioid ovarian cancer" or "ovarian stromal cell tumor" or "granulosa cell tumor of the ovary") and ("single nucleotide polymorphisms"))

China Science and Technology Journal Database (via cqvip)

M= (ovarian carcinomas OR ovarian epithelial carcinomas OR epithelial ovarian carcinomas OR ovarian germinoma OR teratoma OR mucinous ovarian cancer OR serous ovarian cancer OR endometrioid ovarian cancer OR ovarian stromal cell tumor OR granulosa cell tumor of the ovary) AND M= single nucleotide polymorphisms.

China Biology Medicine disc (via sinomed)

("ovarian carcinomas"[Title] OR "ovarian epithelial carcinomas"[Title] OR "epithelial ovarian carcinomas"[Title] OR "ovarian germinoma"[Title] OR "teratoma"[Title] OR " mucinous ovarian cancer"[Title] OR "serous ovarian cancer"[Title] OR "endometrioid ovarian cancer"[Title] OR "ovarian stromal cell tumor"[Title] OR "granulosa cell tumor of the ovary"[Title]) AND ("single nucleotide polymorphisms"[Title])
