# Supplementary material for: The association between single nucleotide polymorphisms and ovarian cancer risk: A systematic review and network meta‐analysis
Source: Cancer Med. 2022 May 30;12(1):541–56. doi: 10.1002/cam4.4891 (PMC9844622; doi:10.1002/cam4.4891)
Supplement: Supplementary file 2 — Supplement Information S2 [file CAM4-12-541-s002.pdf]

Supplement information 2. The data characteristics derived from the selected studies.

First author, year of publication	Studied SNPs	Country	Genotyping method	Sample size (case/control)	P_{HWE}
Abigail W. Bushley,2004	IL-18 rs187238, IL-10 rs1800871	America	ABI 7900 sequence detection system	182/219,181/219	0.99/<0.001
Adrianna Mostowska,2013	Bsm1 rs1544410, Fok1 rs2228570	Poland	PCR-RFLP	168/182,168/182	0.94/0.06
Adrianna Mostowska,2016	Bsm1 rs1544410, Fok1 rs2228570	Poland	PCR-RFLP	245/465,245/465	0.35/0.04
Agnieszka Honorata Ludwig,2009	PGR rs10895068	Poland	TaqMan	251/352	0.94
Alamtaj Samsami Dehaghani,2009	IL-18 rs187238	Iron	PCR	85/158	0.69
Amal M.H. Mackawy,2019	XRCC3 rs861539, XRCC3 rs1799794	Egypt	PCR/RFLP	50/20,50/19	0.47/0.8
Amanda B.Spurdle,2001	EPHX1 rs1051740, PGR rs1042839, PGR rs1042838	Australia	ABI Prism 7700	545/287,551/289, 551/289	0.31/0.17/ 0.17
Andrea Romano,2006	PGR rs1042839, PGR rs10895068	Netherland	PCR-RFLP	67/433,58/379	0.12/0.94
Andrew Berchuck,2004	PGR rs10895068	America<br>Australia	TaqMan	438/504<br>535/298	0.94<br>0.94
Anıl Çağla	MTHFR rs1801133	Turkry	PCR-RFLP	50/54	0.63

Özkılıç,2016					
Anna Jakubowska,2007	MTHFR rs1801133, MTHFR rs1801131, RAD51 rs1801320	Poland	PCR-RFLP	144/280,146/280, 127/127	0.03/0.1/ 0.17
Anna Jakubowska,2010	ERCC2 rs1799793, ERCC2 rs13181, COMT rs4680, PGR rs10895068	Poland	PCR-RFLP	144/280,145/280, 144/280,144/279	0.59/0.42/ 0.53/0.93
Annika Auranen,2005	BRCA1 rs1799950, BRCA1 rs799917, NBS1 rs1063045, NBS1 rs1805794, NBS1 rs709816, NBS1 rs1061302, RAD51 rs1801320, RAD51 rs1801321, RAD52 rs11226, XRCC2 rs3218536, XRCC3 rs1799794, XRCC3 rs1799796, XRCC3 rs861539	UK(SEARCH)<br>US(FROC)<br>Danish(MALOVA)<br>UK(RMH/YOV)	TaqMan<br>PCR<br>PCR<br>PCR	1349/2021,1640/2920, 1661/3528,1586/2792, 1409/3899,1561/3743, 1629/2805,1644/2602, 1649/39061588/3618, 1658/3900,1664/3966 1670/3849	0.18/0.29/ 0.63/0.46/ 0.62/0.40/ 0.82/0.38/ 0.80/0.04/ 0.09/0.41/ 0.75
BEATA SMOLARZ,2013	RAD51 rs1801320, RAD51 rs1801321	Poland	PCR-RFLP	210/210,210/210	0.54/0.51
Beata Smolarz,2019	XRCC2 rs718282, RAD51 rs1801320	Poland	PCR-RFLP	600/600,600/600	0.57/0.55
Catherine M. Phelan,2010	GALNT1 rs17647532	America，England，Australia，Polish，Danish	TaqMan	7794/9314	0.11
D. V. Khokhrin,2012	ERCC2 rs1799793, ERCC2 rs13181, XRCC1 rs25487, GSTP1 rs1695	Russia	PCR	104/298,104/298, 104/298,104/298,	0.66/0.12/ 0.98/0.39

Dan Tong,2001	PGR rs1042839, PGR rs1042838	Australia	PCR	226/194,226/194	0.14/0.14
Daniela B. Leite,2008	PGR rs1042838	Brazil	PCR	182/80	0.21
Delores J. Grant,2013	Apa1 rs7975232, Taq1 rs731236, Bsm1 rs1544410, VDR rs2239179, VDR rs3782905, LINC02354 rs7968585	America	TaqMan	706/765,585/609, 572/601580/607, 581/605,582/609	0.41/0.85/ 0.4/0.78/ 0.46/0.2
Dominique Bernard-Gallon,2008	ERCC2 rs1799793, ERCC2 rs13181	France	TaqMan	51/994,52/995	0.67/0.34
E. Cecchin, 2004	CYP1B1 rs1056836	Italy	PCR-RFLP	220/280	0.45
Ece Konac,2007	VEGF rs833061, VEGF rs3025039	Turkry	PCR	47/104,47/104	0.4/0.2
Elena Ioana Braicu,2006	IL-10 rs1800896, IL-10 rs1800871	Germany	PCR	147/129,197/129	0.46/0.77
Elizabeth I. O. Garner. 2002	COMT rs4680	America	PCR-RFLP	210/225	0.39
Ellen L. Goode,2011	EPHX1 rs1051740	America,Australia	Illumina GoldenGate assay	924/1036	0.01
Emina J. Malisic,2015	RAD51 rs1801320, XRCC1 rs25487	Serbia	PCR-RFLP	50/78,50/78	0.91/0.52
Faten Zahran Mohamed,2013	ERCC2 rs13181, XRCC2 rs3218536, CDKN1B rs2066827	Egypt	PCR	100/100,100/100, 100/100	0.28/0.46/ 0.45
Galina Lurie,2007	APAI rs7975232, BsmI rs1544410, TaqI rs731236, FOk1 rs2228570, Cdx-2 rs11568820	America	PCR-RFLP	163/307,566/601, 161/319,164/316, 162/316	<0.01/<0.01/ <0.01/0.66 /<0.01
Galina Lurie,2009	ESR2 rs3020450	America	TaqMan	313/569	<0.0001

Galina Lurie,2010	PTGS2 rs5275	America	TaqMan	1025/1687	0.04
Galina Lurie,2011	FOK1 rs2228570	Denmark，England，America	TaqMan	1820/3479	0.01
Haifeng Qiu,2017	HOTAIR rs920778	China	TaqMan	190/380,139/300	0.03
Haijing Wu,2016	LncRNA-HOTAIR rs4759314	China	RT-PCR	1000/1000	0.01
Hanna Romanowicz,2016	ERCC2 rs238406	America	PCR	400/100	0.01
Hanna Romanowicz,2017	ERCC2 rs238406	Poland	PCR	400/400	0.54
Harvey A. Risch,2006	PGR rs10895068	America	PCR	487/533	0.96
Hoenil Jo,2007	ERCC1 rs11615	Koera	PCR-RFLP	94/329	0.71
Honglin Song,2006	RB1 rs4151551, RB1 rs3092904, RB1 rs4151636	Denmark，England，America	TaqMan	1486/4310,1332/4191, 1481/4761	0.99/0.88/ 0.27
Honglin Song,2009	H19 rs2107425	Denmark,England,America，Australia	TaqMan	5366/8538	0.86
Houda Bouanene,2011	MUC16 rs2547065	Tunisia	PCR	117/76	0.41
Ian Harley,2008	MLH1 rs1800734	Canada	MassARRAY	310/576	<0.01
János Lukács,2019	miR-146a rs2910164，miR-196a-2 rs11614913	Hungary	PCR	75/75, 75/75	0.09/ 0.38
Jennifer A.	ESR1/SYNE1 rs2295190	America	TaqMan	486/657	0.87

Doherty,2010		America		294/671	0.19
		America		348/538	0.21
		Australia		876/1100	0.06
		Germany		203/427	0.37
		America		69/158	0.37
		Denmark		439/794	0.71
		America		274/366	0.30
		America		610/738	0.62
		America		605/901	0.31
		Poland		254/589	0.88
		England		937/1213	0.35
		America		266/181	1.00
		America		278/420	0.06
		England		468/563	0.05
Jin X,2008	CDKN1B rs2066827	China	PCR-RFLP	234/284	0.34
Johnathan M. Lancaster,1996	EPHX1 rs1051740	America	PCR-RFLP	73/75	0.37
Johnathan M. Lancaster,2003	PGR rs1042838	America	PCR-RFLP	309/397	0.04
Jonathan Beesley,2007	SRD5A2 rs523349, HSD17B4 rs17145454, HSD17B1 rs605059, CYP19A1 rs10046, BRCA2 rs144848, XRCC2 rs3218536, XRCC3 rs861539	Australia	MALDI-TOF	1442/1791, 1418/1774, 1354/1750 1438/1796,1422/1773, 1409/17871235/1674	0.69/ 0.08/ 0.44/ 0.47/0.26/ 0.92/0.62

José Augusto Rinck- Junior,2015	VEGF rs3025039	Brazil	PCR	131/137	0.87
Julie E. Goodman. 2000	COMT rs4680	Germany	PCR-RFLP	108/106	0.86
Karolina Tecza,2015	PGR rs1042838, TP53 rs1042522, GSTP1 rs1695	Poland	ASA-PCR，multiplex-PCR，RFLP-PCR	244/345,225/341, 224/334	0.34/0.93/ 0.30
Kathryn L. Terry,2005	PGR rs10895068， PGR rs1042839，PGR rs1042838	America	TaqMan	920/960,896/939, 896/939	0.95/0.19/ 0.19
Kathryn L. Terry,2010	MTHFR rs1801131, MTHFR rs1801133, GALNT2 rs2271077	America，England	TaqMan	1555/1989,1576/2019,2413/2946	0.302/0.145/ 0.110
Kristina A. Williams,2014	MUC16 rs2547065	America	TaqMan	699/732	0.37
L. YAN,2008	p16/CDKN2 rs11515, p16/CDKN2 rs3088440	China	PCR-RFLP	205/208,205/208	0.05/0.88
Laetitia Delort,2008	CYP1B1 rs1056836, CYP1B1 rs1800440, PGR rs10895068, COMT rs4680	France	TaqMan	51/1000,51/1000,51/1000, 51/1000	0.43/ 0.19/0.92/ 0.48
Leilei Niu,2015	hMLH1 rs1800734	China	MALDI-TOF	421/689	<0.001
Li Li,2015	MCP-1 rs1024611	China	PCR-RFLP	275/293	0.56
Li Yan,2013	Fas rs2234767	China	AS-PCR	342/344	0.68
Li Zhang,2012	MTHFR rs1801133	China	TaqMan	215/218	0.02
Lydia Quaye,2009	H19 rs2107425, XRCC3 rs861539, CDKN2A rs11515, CDKN2A rs3088440	Denmark，England America	TaqMan	1457/2463,1332/2024,1479/2492,1475/2490	<0.01/0.62/ 0.15/0.91

Lyudmila F. Gulyaeva,2008	CYP1A2 rs762551	Russia	PCR-RFLP	96/180	<0.01
M.S. Monteiro,2014	ERCC2 rs1799793, XRCC3 rs861539	Brazil	PCR-RFLP	70/70,70/70	0.02/0.37
Magdalena M. Michalska,2014	XRCC2 rs718282	Poland	PCR-RFLP	608/400	0.48
Magdalena M. Michalska,2016	XRCC2 rs3218536, XRCC3 rs861539	Poland	PCR-RFLP	700/700,700/700	0,54/0.46
Marc T. Goodman, 2001	CYP1B1 rs1056836, COMT rs4680	America	PCR-RFLP	128/144,125/144	0.41/0.21
Marc T. Goodman,2003	CYP1A2 rs762551	America	PCR-RFLP	164/194	0.29
Masatsugu Ueda,2009	MDM2 rs2279744	Tokyo	PCR-RFLP	85/108	0.04
Merete Bjørnslett,2012	MDM2 rs117039649, MDM2 rs2279744	Norway	PCR	1566/2465,2566/2465	0.96/0.67
Mingyao Zhang,2020	APEX1 rs1130409, APEX1 rs1760944	China	TaqMan，PCR	193/231,192/272	0.80/0.41
Narmella Saeedi,2020	LncRNA-HOTAIR rs4759314	Iron	PCR-RFLP	100/100	0.01
Ni, J, and Huang, Y,2016	miR-196a2 rs11614913	China	PCR-RFLP	75/75	0.46
NJ McKenna,1995	PGR rs1042838	Ireland	PCR-RFLP	67/184	0.12
Nora Alyahri,2019	BRCA1 rs799917	Saudi Arabia	TaqMan	28/92	0.98
Penelope M.	RAD51 rs1801320, XRCC2 rs3218536,	Australia	PCR-RFLP	997/2097,954/2068,	0.002/0.164/

Webb,2005	XRCC3 rs861539			991/2077	0.245
Piotr Pawlik,2011	MTHFR rs1801133, MTR rs1805087, MTRR rs1801394	Poland	PCR-RFLP	135/160,136/160, 134/160	0.12/0.17/ 0.97
PM Webb,2011	MTHFR rs1801131, MTR rs1805087, MTRR rs1801394	Australia	MassARRAY	1638/1278,1638/1278,163 8/1228,1638/1277	0.8/0.3/ 0.8/1.0
R. Attar,2017	MCP1 rs1024611	Turkry	PCR-RFLP	56/52	0.15
Rachel T. Palmieri,2008	IL18 rs1834481	Australia,Am erica,England	Illumina	6925/8589	0.32
Rafał Watrowski,2015	HER2 rs1136201	Austria	PCR	142/100	0.53
Richard A. DiCioccio,2004	STK15 rs2273535, STK15 rs1047972, STK15 rs732417, STK15 rs8173	England	TaqMan	752/843,750/843, 750/840,717/812	0.54/0.51/ 0.95/0.14
		America	PCR	308/398,323/427, 319/413,299/401	0.42/0.98/ 0.59/0.50
		Denmark	PCR	334/723,432/1112, 390/949,385/963	0.20/0.96/0.3 4/0.05
Romanowicz-Mak owska H,2012	RAD51 rs1801320	Poland	PCR-RFLP	120/120	0.07
S. Dholariya,2016	TP53 rs1042522	New Delhi	ASO-PCR	70/70	0.09
S.W. Baxter,2002	EPHX1 rs1051740	England	AS-PCR	291/257	0.30
Sandra Costa,2007	ERCC2 rs1799793, ERCC2 rs238406	Portugal	PCR-RFLP	123/199,188/187	0.74/0.49
Santhanam Shanmughapriya,2 013	HER2 rs1136201	India	PCR–RFLP	72/288	0.09

Sarah K. Holt, 2007	CYP1B1 rs1056836, CYP1B1rs1056827, CYP1B1 rs1800440, COMT rs4680	America	TaqMan	310/574,310/576, 310/577,310/576	0.01/0.19/ 0.38/0.08
Shan Kang,2004	EPHX1 rs1051740	China	PCR-RFLP	86/174	<0.001
Shan Kang,2008	MDM2 rs2279744	China	PCR	257/257	0.45
Shan-Yang He,2012	ERCC1 rs11615	China	TaqMan	155/312	0.77
Shelley S. Tworoger,2009	Bsm1 rs1544410, Fok1 rs2228570, Cdx2 rs11568820	America	TaqMan	1391/1885,1392/1894, 1405/1910	0.11/0.06/ 0.02
Simon A. Gayther,2007	CDKN2A rs3731249, CDKN2A rs11515, CDKN1B rs2066827	England	TaqMan	1478/2452,1053/1276, 1495/2484	0.39/0.38/ 0.004
Simone P. Pinheiro,2010	PPAR-γ/PPARG rs1801282, COX-2 rs20417, COX-2 rs5275	America	TaqMan	224/644,1075/1136, 224/645,1050/1111, 217/612,1046/1103	0.12/ 0.82/0.34
Song CX,2012	MTHFR rs1801131	China	PCR	200/200	0.29
Song Gao,2012	MTHFR rs1801133	China	PCR-RFLP	224/432	0.26
Stian Knappskog,2011	MDM2 rs117039649	Norway， Netherland	PCR	832/2518	0.96
Susanne Schüler,2014	ESR2 rs3020450	Poland， Germany	PCR	182/184	0.65
Tadeusz Dȩbniak,2006	CDKN2A rs3731249	Poland	PCR	340/3000	0.02
Tess V. Clendenen,2008	BSMI rs1544410, APAI rs7975232, TAQI rs731236, FOK1 rs2228570	America,Swe den	pyrosequencing	168/318,168/320, 169/320,168/321	0.2/0.37/ 0.07/0.94

Thomas A. Sellers, 2005	CYP1B1 rs1056836, CYP1B1 rs1056827, COMT rs4680	America	PCR	490/598,490/596,489/596	0.45/ 0.28/0.5
Thomas A. Sellers,2008	GALNT1 rs17647532, FUT3 rs2306969, GALNT6 rs907352, GALNT7 rs934358, MGAT5 rs1257187, ST3GAL3 rs3828139, ST3GAL3 rs37460	America	PCR	385/461, 444/478,829/939, 827/939,829/940, 829/940, 828/941	<0.001/ 0.02/0.02/ 0.01/0.02/ 0.04/ 0.05
TP Manolitsas,1997	PGR rs1042838	England	PCR-RFLP	231/220	0.14
UZAY GORMUS,2007	Fas rs2234767, FasL rs763110	Turkry	PCR	47/41,47/41	0.27/0.68
Vidudala V.T.S. Prasad,2011	MTHFR rs1801133	India	PCR-RFLP	80/125	0.96
Wendy M. Smith,2001	PPARG rs1801282	England	PCR	31/62	0.61
		Tokyo	PCR	28/215	1.00
		America	PCR	26/80	0.43
X.C. Sun,2016	miR-146a rs2910164, miR-196a2 rs11614913	China	PCR	134/277, 134/277	0.37/ 0.36
Xiaohong Zhang,2013	APE1 rs1760944, APE1 rs1130409	China	PCR	124/141,124/141	0.56/0.87
Xiaoyan Liu,2015	miR-146a rs2910164, miR-196a2 rs11614913	China	PCR-RFLP	75/100, 75/100	<0.01/ 0.44
Xin Wei,2015	MCP-1 rs1024611	China	PCR–RFLP	257/273	0.58
Yajing Feng,2019	ESR2 rs3020450	China	TaqMan	680/680	0.71

Yuan C,2015	Fok1 rs2228570	China	PCR	129/298	0.43
Yuxia Bao,2020	ERCC-1 rs11615, ERCC-1 rs3212986, ERCC-1 rs2298881	China	PCR	599/323, 599/323,599/323	0.23/ 0.2/0.24
Zahra Mojtahedi,2013	HER2 rs1136201	Iron	PCR–RFLP	107/130	0.14
Zhiguang Zhao,2018	ERCC1 rs3212986, ERCC1 rs11615, ERCC1 rs2298881	China	Real-time PCR	89/356, 89/356,89/256	0.840/ 0.895/0.9
ZHI-SHUANG SONG,2016	miR-196a2 rs11614913	China	PCR	479/431	0.44
Yali Hao,2012	FASL rs763110	China	PCR-RFLP	342/344	0.74
Xiuping He,2008	IL-10 rs1800896, IL-10 rs1800871	China	PCR	33/90,33/90	0.11/0.31
Jinghui Jia,2009	VEGF rs3025039	China	PCR-RFLP	256/329	0.86
Pengfei Liu,2007	XRCC2 rs718282	China	PCR-RFLP	220/244	0.91
Yang Ruan,2014	MCP-1 rs1024611	China	PCR-RFLP	92/38	0.62
Wengang Si,2019	hMLH1 rs1800734	China	PCR-RFLP	536/529	0.21
Yan Wang,2010	VEGF rs833061	China	PCR-RFLP	303/303	0.21
Yan Wu,2007	MTHFR rs1801133	China	PCR	81/86	0.02
Yanping Xing,2007	XPD rs1799793	China	PCR-RFLP	235/246	0.92
Anqi Zhang,2019	ERCC1 rs3212986, ERCC1 rs11615, ERCC1 rs2298881	China	TaqMan	192/272, 193/271,192/272	0.167/ 0.111/0.840
