# Supplementary material for: The association between single nucleotide polymorphisms and ovarian cancer risk: A systematic review and network meta‐analysis
Source: Cancer Med. 2022 May 30;12(1):541–56. doi: 10.1002/cam4.4891 (PMC9844622; doi:10.1002/cam4.4891)
Supplement: Supplementary file 3 — Supplement Information S3 [file CAM4-12-541-s005.pdf]

Supplement information 3. The methodological quality evaluation results

	①	②	③	④	⑤	⑥	⑦	⑧	⑨	Total points
Abigail W. Bushley, 2004	1	0	0	1	0	1	1	0	1	5
Adrianna Mostowska, 2013	1	0	0	1	0	1	1	0	1	5
Adrianna Mostowska, 2016	1	0	0	1	0	1	1	0	1	5
Agnieszka Honorata Ludwig, 2009	1	0	0	1	0	1	1	0	1	5
Alamtaj Samsami Dehaghani, 2009	1	0	0	1	0	1	1	0	0	4
Amal M.H. Mackawy, 2019	1	1	0	1	0	1	1	0	0	5
Amanda B.Spurdle, 2001	1	1	0	1	0	1	1	0	1	6
Andrea Romano, 2006	1	0	0	1	0	1	1	0	0	4
Andrew Berchuck, 2004	1	0	0	1	0	1	1	0	1	5
Anıl Çağla Özkılıç, 2016	1	0	0	1	0	1	1	0	0	4
Anna Jakubowska, 2007	1	0	0	1	0	1	1	0	1	5
Anna Jakubowska, 2010	1	0	0	1	0	1	1	0	1	5
Annika Auranen, 2005	1	0	0	1	0	1	1	0	1	5
BEATA SMOLARZ, 2013	1	0	0	1	0	1	1	0	1	5
Beata Smolarz, 2019	1	0	0	1	0	1	1	0	1	5
Catherine M. Phelan, 2010	1	0	0	1	0	1	1	0	1	5
D. V. Khokhrin, 2012	1	0	0	1	0	1	1	0	1	5
Dan Tong, 2001	1	0	0	1	0	1	1	0	1	5
Daniela B. Leite, 2008	1	0	0	1	0	1	1	0	1	5

Delores J. Grant, 2013	1	0	0	1	0	1	1	0	1	5
Dominique Bernard-Gallon, 2008	1	0	0	1	0	1	1	0	1	5
E. Cecchin, 2004	1	1	0	1	0	1	1	0	1	6
Ece Konac, 2007	1	0	0	1	0	1	1	0	0	4
Elena Ioana Braicu, 2006	1	0	0	1	0	1	1	0	1	5
Elizabeth I. O. Garner, 2002	1	0	0	1	0	1	1	0	1	5
Ellen L. Goode, 2011	1	0	0	1	0	1	1	0	1	5
Emina J. Malisic, 2015	1	1	1	1	0	1	1	0	0	6
Faten Zahran Mohamed, 2013	1	1	0	1	0	1	1	0	0	5
Galina Lurie, 2007	1	0	0	1	0	1	1	0	1	5
Galina Lurie, 2009	1	0	0	1	0	1	1	0	0	4
Galina Lurie, 2010	1	0	1	1	0	1	1	0	1	6
Galina Lurie, 2011	1	1	0	1	0	1	1	0	1	6
Haifeng Qiu, 2017	1	0	0	1	0	1	1	0	1	5
Haijing Wu, 2016	1	0	0	1	0	1	1	0	1	5
Hanna Romanowicz, 2016	1	0	0	1	0	1	1	0	1	5
Hanna Romanowicz, 2017	1	0	0	1	0	1	1	0	1	5
Harvey A. Risch, 2006	1	1	0	1	0	1	1	0	0	5
Hoenil Jo, 2007	1	0	0	1	0	1	1	0	1	5
Honglin Song, 2006	1	0	0	1	0	1	1	0	1	5
Honglin Song, 2009	1	0	0	1	0	1	1	0	1	5
Houda Bouanene, 2011	1	0	0	1	0	1	1	0	0	4

Ian Harley, 2008	1	0	0	1	0	1	1	0	1	5
János Lukács, 2019	1	0	0	1	0	1	1	0	1	5
Jennifer A. Doherty, 2010	1	0	0	1	0	1	1	0	1	5
Jin X, 2008	1	0	0	1	0	1	1	0	1	5
Johnathan M. Lancaster, 1996	1	0	0	1	0	1	1	0	0	4
Johnathan M. Lancaster, 2003	1	0	0	1	0	1	1	0	1	5
Jonathan Beesley, 2007	1	0	0	1	0	1	1	0	1	5
José Augusto Rinck- Junior, 2015	1	0	0	1	0	1	1	0	0	4
Julie E. Goodman, 2000	1	0	0	1	0	1	1	0	1	5
Karolina Tecza, 2015	1	1	0	1	0	1	1	0	1	6
Kathryn L. Terry, 2005	1	0	0	1	0	1	1	0	1	5
Kathryn L. Terry, 2010	1	0	0	1	0	1	1	0	1	5
Kristina A. Williams, 2014	1	0	0	1	0	1	1	0	1	5
L. YAN, 2008	1	0	0	1	0	1	1	0	1	5
Laetitia Delort, 2008	1	0	0	1	0	1	1	0	1	5
Leilei Niu, 2015	1	0	0	1	0	1	1	0	1	5
Li Li, 2015	1	0	0	1	0	1	1	0	1	5
Li Yan, 2013	1	0	0	1	0	1	1	0	1	5
Li Zhang, 2012	1	1	0	1	0	1	1	0	1	6
Lydia Quaye, 2009	1	0	0	1	0	1	1	0	1	5
Lyudmila F. Gulyaeva, 2008	1	0	0	1	0	1	1	0	0	4
M.S. Monteiro, 2014	1	0	0	1	0	1	1	0	0	4

Magdalena M. Michalska, 2014	1	0	0	1	0	1	1	0	1	5
Magdalena M. Michalska, 2016	1	0	0	1	0	1	1	0	1	5
Marc T. Goodman, 2001	1	0	0	1	0	1	1	0	1	5
Marc T. Goodman, 2003	1	0	0	1	0	1	1	0	1	5
Masatsugu Ueda, 2009	1	0	0	1	0	1	1	0	1	5
Merete Bjørnslett, 2012	1	0	0	1	0	1	1	0	1	5
Mingyao Zhang, 2021	1	0	0	1	0	1	1	0	1	5
Narmella Saeedi, 2020	1	0	0	1	0	1	1	0	0	4
Ni, J, and Huang, Y, 2016	1	0	0	1	1	1	1	0	1	6
NJ McKenna, 1995	1	0	0	1	0	1	1	0	0	4
Nora Alyahri, 2019	1	0	0	1	0	1	1	0	0	4
Penelope M. Webb, 2005	1	0	0	1	0	1	1	0	1	5
Piotr Pawlik, 2011	1	0	0	1	0	1	1	0	1	5
PM Webb, 2011	1	0	0	1	0	1	1	0	1	5
R. Attar, 2017	1	0	0	1	0	1	1	0	0	4
Rachel T. Palmieri, 2008	1	0	0	1	0	1	1	0	1	5
Rafał Watrowski, 2015	1	0	0	1	0	1	1	0	0	4
Richard A. DiCioccio, 2004	1	0	0	1	0	1	1	0	1	5
Romanowicz-Makowska H, 2012	1	0	0	1	0	1	1	0	0	4
S. Dholariya, 2016	1	0	0	1	0	1	1	0	0	4
S.W. Baxter, 2002	1	0	0	1	0	1	1	0	1	5
Sandra Costa, 2007	1	0	0	1	0	1	1	0	1	5

Santhanam Shanmughapriya, 2013	1	0	0	1	0	1	1	0	0	4
Sarah K. Holt, 2007	1	0	0	1	0	1	1	0	1	5
Shan Kang, 2004	1	0	0	1	0	1	1	0	0	4
Shan Kang, 2008	1	0	0	1	0	1	1	0	1	5
Shan-Yang He, 2012	1	0	0	1	0	1	1	0	1	5
Shelley S. Tworoger, 2009	1	1	0	1	0	1	1	0	1	6
Simon A. Gayther, 2007	1	0	0	1	0	1	1	0	1	5
Simone P. Pinheiro, 2010	1	0	0	1	0	1	1	0	1	5
Song CX, 2012	1	0	0	1	0	1	1	0	1	5
Song Gao, 2012	1	0	0	1	0	1	1	0	1	5
Stian Knappskog, 2011	1	0	0	1	0	1	1	0	1	5
Susanne Schüler, 2014	1	0	0	1	0	1	1	0	1	5
Tadeusz Dębniak, 2006	1	0	0	1	0	1	1	0	1	5
Tess V. Clendenen, 2008	1	0	0	1	0	1	1	0	0	4
Thomas A. Sellers, 2005	1	0	1	1	0	1	1	0	1	6
Thomas A. Sellers, 2008	1	0	0	1	0	1	1	0	1	5
TP Manolitsas, 1997	1	0	0	1	0	1	1	0	1	5
UZAY GORMUS, 2007	1	0	0	1	0	1	1	0	0	4
Vidudala V.T.S. Prasad, 2011	1	0	0	1	0	1	1	0	0	4
Wendy M. Smith, 2001	1	0	0	1	0	1	1	0	1	5
X.C. Sun, 2016	1	0	0	1	0	1	1	0	1	5
Xiaohong Zhang, 2013	1	0	1	1	0	1	1	0	1	6

Xiaoyan Liu, 2015	1	0	0	1	0	1	1	0	0	4
Xin Wei, 2015	1	0	0	1	0	1	1	0	1	5
Yajing Feng, 2019	1	0	0	1	0	1	1	0	1	5
Yuan C, 2015	1	0	0	1	0	1	1	0	1	5
Yuxia Bao, 2020	1	1	0	1	0	1	1	0	1	6
Zahra Mojtahedi, 2013	1	0	0	1	0	1	1	0	0	4
Zhiguang Zhao, 2018	1	0	0	1	0	1	1	0	1	5
ZHI-SHUANG SONG, 2016	1	0	0	1	0	1	1	0	1	5
Zhiya Hao, 2012	1	1	0	1	0	1	1	0	1	6
Xiuping He, 2008	1	0	0	1	0	1	1	0	0	4
Jinghui Jia, 2009	1	1	0	1	0	1	1	0	1	6
Pengfei Liu, 2007	1	1	0	1	0	1	1	0	1	6
Yang Ruan, 2014	1	0	0	1	0	1	1	0	0	4
Wengang Si, 2019	1	1	0	1	0	1	1	0	1	6
Yan Wang, 2010	1	1	0	1	0	1	1	0	1	6
Yan Wu, 2007	1	0	0	1	0	1	1	0	0	4
Yanping Xing, 2007	1	1	0	1	0	1	1	0	1	6
Anqi Zhang, 2019	1	1	0	1	0	1	1	0	1	6

Note: ①whether genotyping methods were mentioned, ②whether to stratify the targeted population, ③whether to provide methods to infer genotypes, ④if the control group satisfied Hardy-Weinberg equilibrium (HWE), ⑤if the study could be

repeated, ⑥if targeted population, inclusion, and exclusion criteria were described, ⑦whether to describe statistical methods and software, ⑧whether to provide methodologies of multiple comparisons, handling false-positive findings or correcting relatedness, ⑨ whether the provided information was sufficient.
