# Supplementary material for: The association between single nucleotide polymorphisms and ovarian cancer risk: A systematic review and network meta‐analysis
Source: Cancer Med. 2022 May 30;12(1):541–56. doi: 10.1002/cam4.4891 (PMC9844622; doi:10.1002/cam4.4891)
Supplement: Supplementary file 4 — Supplement Information S4 [file CAM4-12-541-s004.pdf]

**Supplement information 4. Risk of bias (ROBINS-1) assessment for included studies.**

| | ① | ② | ③ | ④ | ⑤ | ⑥ | ⑦ | Overall |
|---|---|---|---|---|---|---|---|---|
| Abigail W. Bushley, 2004 | + | + | + | + | × | + | × | + |
| Adrianna Mostowska, 2013 | + | + | + | + | + | + | + | + |
| Adrianna Mostowska, 2016 | + | + | + | + | + | + | + | + |
| Agnieszka Honorata Ludwig, 2009 | × | + | + | + | + | + | × | + |
| Alamtaj Samsami Dehaghani, 2009 | × | + | + | + | + | + | + | + |
| Amal M.H. Mackawy, 2019 | × | + | + | + | + | + | × | + |
| Amanda B.Spurdle, 2001 | + | + | + | + | + | + | × | + |
| Andrea Romano, 2006 | + | + | + | + | + | + | × | + |
| Andrew Berchuck, 2004 | + | + | + | + | + | + | + | + |
| Anıl Çağla Özkılıç, 2016 | × | + | + | + | + | + | + | + |
| Anna Jakubowska, 2007 | + | + | + | + | + | + | × | + |
| Anna Jakubowska, 2010 | × | + | + | + | + | + | + | + |
| Annika Auranen, 2005 | + | + | + | + | × | + | × | + |
| BEATA SMOLARZ, 2013 | + | + | + | + | + | + | + | + |
| Beata Smolarz, 2019 | × | + | + | + | + | + | + | + |
| Catherine M. Phelan, 2010 | + | + | + | + | × | + | × | + |
| D. V. Khokhrin, 2012 | × | + | + | + | × | + | × | × |
| Dan Tong, 2001 | + | + | + | + | + | + | + | + |
| Daniela B. Leite, 2008 | × | + | + | + | + | + | × | + |
| Delores J. Grant, 2013 | + | + | + | + | + | + | + | + |

| | | | | | | | | |
|---|---|---|---|---|---|---|---|---|
| Dominique Bernard-Gallon, 2008 | × | + | + | + | × | + | + | + |
| E. Cecchin, 2004 | × | + | + | + | + | + | + | + |
| Ece Konac, 2007 | + | + | + | + | + | + | + | + |
| Elena Ioana Braicu, 2006 | × | + | + | + | + | + | + | + |
| Elizabeth I. O. Garner, 2002 | + | + | + | + | + | + | × | + |
| Ellen L. Goode, 2011 | + | + | + | + | + | + | + | + |
| Emina J. Malisic, 2015 | + | + | + | + | + | + | + | + |
| Faten Zahran Mohamed, 2013 | + | + | + | + | + | + | + | + |
| Galina Lurie, 2007 | + | + | + | + | + | + | + | + |
| Galina Lurie, 2009 | + | + | + | + | × | + | × | + |
| Galina Lurie, 2010 | + | + | + | + | + | + | × | + |
| Galina Lurie, 2011 | × | + | + | + | + | + | × | + |
| Haifeng Qiu, 2017 | + | + | + | + | + | + | + | + |
| Haijing Wu, 2016 | + | + | + | + | + | + | + | + |
| Hanna Romanowicz, 2016 | + | + | + | + | + | + | + | + |
| Hanna Romanowicz, 2017 | + | + | + | + | + | + | × | + |
| Harvey A. Risch, 2006 | + | + | + | + | + | + | + | + |
| Hoenil Jo, 2007 | × | + | + | + | + | + | + | + |
| Honglin Song, 2006 | × | + | + | + | × | + | × | × |
| Honglin Song, 2009 | + | + | + | + | + | + | + | + |
| Houda Bouanene, 2011 | × | + | + | + | + | + | + | + |
| Ian Harley, 2008 | × | + | + | + | × | + | × | × |

| | | | | | | | | |
|---|---|---|---|---|---|---|---|---|
| János Lukács, 2019 | × | + | + | + | + | + | + | + |
| Jennifer A. Doherty, 2010 | × | + | + | + | × | + | × | × |
| Jin X, 2008 | × | + | + | + | + | + | + | + |
| Johnathan M. Lancaster, 1996 | + | + | + | + | + | + | + | + |
| Johnathan M. Lancaster, 2003 | + | + | + | + | + | + | × | + |
| Jonathan Beesley, 2007 | + | + | + | + | + | + | + | + |
| José Augusto Rinck-Junior, 2015 | × | + | + | + | + | + | + | + |
| Julie E. Goodman, 2000 | × | + | + | + | + | + | + | + |
| Karolina Tecza, 2015 | + | + | + | + | + | + | + | + |
| Kathryn L. Terry, 2005 | + | + | + | + | + | + | × | + |
| Kathryn L. Terry, 2010 | + | + | + | + | + | + | + | + |
| Kristina A. Williams, 2014 | + | + | + | + | + | + | + | + |
| L. YAN, 2008 | × | + | + | + | + | + | + | + |
| Laetitia Delort, 2008 | × | + | + | + | + | + | + | + |
| Leilei Niu, 2015 | + | + | + | + | + | + | + | + |
| Li Li, 2015 | + | + | + | + | + | + | + | + |
| Li Yan, 2013 | × | + | + | + | + | + | + | + |
| Li Zhang, 2012 | + | + | + | + | + | + | + | + |
| Lydia Quaye, 2009 | + | + | + | + | + | + | × | + |
| Lyudmila F. Gulyaeva, 2008 | × | + | + | + | + | + | + | + |
| M.S. Monteiro, 2014 | + | + | + | + | + | + | + | + |
| Magdalena M. Michalska, 2014 | + | + | + | + | + | + | × | + |

| | | | | | | | | |
|---|---|---|---|---|---|---|---|---|
| Magdalena M. Michalska, 2016 | + | + | + | + | + | + | + | + |
| Marc T. Goodman, 2001 | + | + | + | + | + | + | × | + |
| Marc T. Goodman, 2003 | + | + | + | + | + | + | × | + |
| Masatsugu Ueda, 2009 | × | + | + | + | + | + | × | + |
| Merete Bjørnslett, 2012 | + | + | + | + | + | + | + | + |
| Mingyao Zhang, 2021 | + | + | + | + | × | + | × | + |
| Narmella Saeedi, 2020 | + | + | + | + | + | + | + | + |
| Ni, J, and Huang, Y, 2016 | + | + | + | + | + | + | + | + |
| NJ McKenna, 1995 | × | + | + | + | + | + | × | + |
| Nora Alyahri, 2019 | × | + | + | + | + | + | + | + |
| Penelope M. Webb, 2005 | + | + | + | + | + | + | × | + |
| Piotr Pawlik, 2011 | + | + | + | + | + | + | + | + |
| PM Webb, 2011 | + | + | + | + | + | + | + | + |
| R. Attar, 2017 | + | + | + | + | + | + | + | + |
| Rachel T. Palmieri, 2008 | + | + | + | + | + | + | × | + |
| Rafał Watrowski, 2015 | + | + | + | + | + | + | + | + |
| Richard A. DiCioccio, 2004 | + | + | + | + | + | + | × | + |
| Romanowicz-Makowska H, 2012 | + | + | + | + | + | + | + | + |
| S. Dholariya, 2016 | + | + | + | + | + | + | + | + |
| S.W. Baxter, 2002 | × | + | + | + | + | + | × | + |
| Sandra Costa, 2007 | × | + | + | + | + | + | × | + |
| Santhanam Shanmughapriya, 2013 | + | + | + | + | + | + | × | + |

| | | | | | | | | |
|---|---|---|---|---|---|---|---|---|
| Sarah K. Holt, 2007 | + | + | + | + | + | + | × | + |
| Shan Kang, 2004 | + | + | + | + | + | + | + | + |
| Shan Kang, 2008 | + | + | + | + | + | + | × | + |
| Shan-Yang He, 2012 | + | + | + | + | + | + | × | + |
| Shelley S. Tworoger, 2009 | + | + | + | + | × | + | + | + |
| Simon A. Gayther, 2007 | + | + | + | + | × | + | × | + |
| Simone P. Pinheiro, 2010 | + | + | + | + | + | + | + | + |
| Song CX, 2012 | + | + | + | + | + | + | + | + |
| Song Gao, 2012 | + | + | + | + | + | + | + | + |
| Stian Knappskog, 2011 | × | + | + | + | + | + | × | + |
| Susanne Schüler, 2014 | + | + | + | + | × | + | × | + |
| Tadeusz Debniak, 2006 | + | + | + | + | + | + | × | + |
| Tess V. Clendenen, 2008 | + | + | + | + | × | + | + | + |
| Thomas A. Sellers, 2005 | + | + | + | + | × | + | × | + |
| Thomas A. Sellers, 2008 | + | + | + | + | × | + | + | + |
| TP Manolitsas, 1997 | × | + | + | + | × | + | × | × |
| UZAY GORMUS, 2007 | × | + | + | + | + | + | + | + |
| Vidudala V.T.S. Prasad, 2011 | × | + | + | + | + | + | × | + |
| Wendy M. Smith, 2001 | × | + | + | + | + | + | + | + |
| X.C. Sun, 2016 | × | + | + | + | + | + | + | + |
| Xiaohong Zhang, 2013 | × | + | + | + | + | + | × | + |
| Xiaoyan Liu, 2015 | × | + | + | + | + | + | + | + |

| | | | | | | | | |
|---|---|---|---|---|---|---|---|---|
| Xin Wei, 2015 | + | + | + | + | + | + | + | + |
| Yajing Feng, 2019 | + | + | + | + | + | + | + | + |
| Yuan C, 2015 | + | + | + | + | + | + | + | + |
| Yuxia Bao, 2020 | + | + | + | + | + | + | + | + |
| Zahra Mojtahedi, 2013 | + | + | + | + | + | + | + | + |
| Zhiguang Zhao, 2018 | + | + | + | + | × | + | + | + |
| ZHI-SHUANG SONG, 2016 | + | + | + | + | + | + | + | + |
| Zhiya Hao, 2012 | + | + | + | + | + | + | + | + |
| Xiuping He, 2008 | × | + | + | + | + | + | + | + |
| Jinghui Jia, 2009 | × | + | + | + | + | + | + | + |
| Pengfei Liu, 2007 | + | + | + | + | + | + | + | + |
| Yang Ruan, 2014 | × | + | + | + | + | + | + | + |
| Wengang Si, 2019 | + | + | + | + | + | + | + | + |
| Yan Wang, 2010 | + | + | + | + | + | + | + | + |
| Yan Wu, 2007 | + | + | + | + | + | + | + | + |
| Yanping Xing, 2007 | + | + | + | + | + | + | + | + |
| Anqi Zhang, 2019 | + | + | + | + | + | + | + | + |

**Judgement:** + Low × Moderate

**Note:** ①Bias due to confounding; ②Bias due to selection of participants; ③Bias in classification of interventions; ④Bias due to deviations from intended interventions; ⑤Bias due to missing data; ⑥Bias in measurement of outcomes; ⑦Bias in selection of the reported result.
