# Supplementary material for: The association between single nucleotide polymorphisms and ovarian cancer risk: A systematic review and network meta‐analysis
Source: Cancer Med. 2022 May 30;12(1):541–56. doi: 10.1002/cam4.4891 (PMC9844622; doi:10.1002/cam4.4891)
Supplement: Supplementary file 5 — Supplement Information S5 [file CAM4-12-541-s001.pdf]

Supplement Information 5. Pairwise meta-analysis of selected SNPs in association with risk for ovarian cancer

Genetic model	Sample size		Heterogeneity	Model	OR (95%CI)	P
	Case	Control	I^2			
APE1 rs1130409	317	372				
G vs T			59.6%	Random	0.590(0.418-0.834)	0.003
GG vs TT			24.2%	Fixed	0.364(0.235-0.565)	<0.001
TG vs TT			0.0%	Fixed	0.619(0.433-0.885)	0.009
GG+TG vs TT			26.7%	Fixed	0.525(0.375-0.735)	<0.001
GG vs TG+TT			0.0%	Fixed	0.494(0.340-0.718)	<0.001
TT+GG vs TG			0.0%	Fixed	1.043(0.772-1.409)	0.784
APE1 rs1760944	316	413				
G vs T			49.9%	Fixed	1.034(0.838-1.276)	0.756
GG vs TT			35.7%	Fixed	1.071(0.707-1.622)	0.746
TG vs TT			31.2%	Fixed	1.001(0.721-1.390)	0.995
GG+TG vs TT			49.4%	Fixed	1.022(0.753-1.388)	0.887
GG vs TG+TT			0.0%	Fixed	1.074(0.740-1.560)	0.707
TT+GG vs TG			0.0%	Fixed	1.024(0.763-1.375)	0.874
XRCC1 rs25487	154	376				
A vs G			87.6%	Random	0.620(0.249-1.542)	0.304
AA vs GG			81.9%	Random	0.443(0.094-2.089)	0.304

GA vs GG			0.0%	Fixed	0.914(0.606-1.377)	0.666
AA+GA vs GG			66.0%	Random	0.695(0.338-1.430)	0.323
AA vs GA+GG			82.0%	Random	0.468(0.106-2.064)	0.316
GG+AA vs GA			0.0%	Fixed	0.961(0.651-1.418)	0.840
XRCC2 rs718282	1428	1244				
T vs C			90.3%	Random	1.770(1.156-2.709)	0.009
TT vs CC			65.9%	Random	2.599(1.600-4.221)	<0.001
CT vs CC			38.8%	Fixed	0.759(0.618-0.933)	0.009
TT+CT vs CC			25.9%	Fixed	1.372(1.143-1.646)	0.002
TT vs CT+CC			85.8%	Random	3.211(1.707-6.037)	<0.001
CC+TT vs CT			92.7%	Random	1.959(0.999-3.842)	0.050
ERCC1 rs3212986	840	951				
A vs C			8.5%	Fixed	1.168(1.006-1.356)	0.042
AA vs CC			0.0%	Fixed	1.565(1.148-2.135)	0.005
CA vs CC			0.0%	Fixed	0.916(0.738-1.138)	0.428
AA+CA vs CC			0.0%	Fixed	1.047(0.857-1.280)	0.652
AA vs			0.0%	Fixed	1.636(1.220-2.1	0.001

CA+CC					94)	
CC+AA vs CA			0.0%	Fixed	1.215(0.991-1.489)	0.061
RAD51 rs1801320	3733	6019				
C vs G			95.5%	Random	1.581(0.969-2.580)	0.067
CC vs GG			77.3%	Random	2.545(1.265-5.118)	0.009
GC vs GG			85.9%	Random	0.838(0.592-1.187)	0.321
CC+GC vs GG			73.3%	Random	1.251(0.987-1.587)	0.064
CC vs GC+GG			88.8%	Random	2.775(1.184-6.503)	0.019
GG+CC vs GC			96.8%	Random	1.676(0.849-3.309)	0.137
LncRNA-HOTAIR rs4759314	1100	1100				
G vs A			89.7%	Random	3.789(0.386-37.212)	0.253
GG vs AA			58.2%	Random	3.684(0.460-29.518)	0.219
AG vs AA			75.8%	Random	2.179(0.462-10.286)	0.325
GG+AG vs AA			86.2%	Random	3.121(0.414-23.549)	0.270
GG vs AG+AA			55.4%	Random	3.428(0.470-25.025)	0.224
AA+GG vs AG			74.0%	Random	0.487(0.110-2.157)	0.343
TP53 rs1042522	295	411				
C vs G			92.0%	Random		0.597

					1.317(0.474-3.655)	
CC vs GG			83.0%	Random	1.822(0.365-9.101)	0.465
GC vs GG			89.0%	Random	1.209(0.358-4.082)	0.760
CC+GC vs GG			91.7%	Random	1.332(0.355-5.003)	0.671
CC vs GC+GG			63.6%	Random	1.580(0.558-4.469)	0.389
GG+CC vs GC			81.6%	Random	0.968(0.399-2.346)	0.942
ERCC1 rs11615	808	964				
T vs C			0.0%	Fixed	1.158(0.986-1.360)	0.073
TT vs CC			0.0%	Fixed	1.639(1.140-2.357)	0.008
CT vs CC			0.0%	Fixed	0.954(0.767-1.186)	0.672
TT+CT vs CC			0.0%	Fixed	1.064(0.869-1.304)	0.548
TT vs CT+CC			0.0%	Fixed	1.663(1.170-2.365)	0.005
CC+TT vs CT			0.0%	Fixed	1.126(0.912-1.390)	0.270
ERCC1 rs11615	282	627				
A vs G			77.5%	Random	0.702(0.409-1.205)	0.199
AA vs GG			46.8%	Fixed	0.819(0.454-1.476)	0.506
GA vs GG			69.1%	Random	0.571(0.315-1.0	0.065

					35)	
AA+GA vs GG			75.2%	Random	0.595(0.318-1.111)	0.103
AA vs GA+GG			16.3%	Fixed	1.012(0.566-1.811)	0.967
GG+AA vs GA			58.8%	Random	1.696(1.024-2.810)	0.04
ERCC1 rs2298881	840	951				
A vs C			0.0%	Fixed	1.079(0.933-1.247)	0.305
AA vs CC			0.0%	Fixed	1.211(0.893-1.641)	0.219
CA vs CC			0.0%	Fixed	1.031(0.829-1.283)	0.782
AA+CA vs CC			0.0%	Fixed	1.069(0.870-1.313)	0.528
AA vs CA+CC			0.0%	Fixed	1.174(0.887-1.553)	0.262
CC+AA vs CA			0.0%	Fixed	1.020(0.835-1.245)	0.848
BRCA1 rs799917	1656	3012				
C vs T			0.0%	Fixed	1.038(0.946-1.139)	0.432
CC vs TT			0.0%	Fixed	1.004(0.814-1.239)	0.969
TC vs TT			2.2%	Fixed	1.108(0.972-1.264)	0.124
CC+TC vs TT			0.0%	Fixed	1.087(0.959-1.232)	0.190
CC vs TC+TT			0.0%	Fixed	0.957(0.783-1.1	0.664

					68)	
TT+CC vs TC			0.0%	Fixed	0.904(0.798-1.023)	0.11
ERCC2 rs238406	918	687				
C vs A			94.5%	Random	0.808(0.391-1.670)	0.566
CC vs AA			91.8%	Random	0.793(0.263-2.395)	0.681
AC vs AA			88.0%	Random	0.700(0.320-1.533)	0.373
CC+AC vs AA			92.0%	Random	0.729(0.303-1.755)	0.48
CC vs AC+AA			86.1%	Random	0.950(0.461-1.960)	0.890
AA+CC vs AC			46.1%	Fixed	1.438(1.161-1.781)	0.001
ERCC2 rs13181	400	1673				
C vs A			75.4%	Random	1.162(0.810-1.665)	0.415
CC vs AA			73.1%	Random	1.099(0.475-2.544)	0.825
AC vs AA			62.8%	Random	1.495(0.965-2.318)	0.072
CC+AC vs AA			71.8%	Random	1.416(0.876-2.289)	0.156
CC vs AC+AA			62.9%	Random	0.938(0.490-1.798)	0.848
AA+CC vs AC			50.5%	Random	0.696(0.491-0.987)	0.042
MCP-1	680	656				

rs1024611						
G vs A			0.0%	Fixed	1.419(1.210-1.664)	<0.001
GG vs AA			0.0%	Fixed	1.868(1.332-2.618)	<0.001
AG vs AA			16.4%	Fixed	1.408(1.033-1.918)	0.03
GG+AG vs AA			0.0%	Fixed	1.620(1.210-2.170)	0.001
GG vs AG+AA			0.0%	Fixed	1.541(1.222-1.945)	<0.001
AA+GG vs AG			53.4%	Random	1.051(0.731-1.512)	0.787
miR-146a rs2910164	284	402				
C vs G			61.4%	Random	0.465(0.312-0.691)	<0.001
CC vs GG			5.8%	Fixed	0.193(0.114-0.325)	<0.001
GC vs GG			81.6%	Random	0.329(0.126-0.860)	0.023
CC+GC vs GG			80.4%	Random	0.293(0.121-0.709)	0.006
CC vs GC+GG			0.0%	Fixed	0.486(0.335-0.705)	<0.001
GG+CC vs GC			75.6%	Random	1.492(0.764-2.914)	0.241
miR-196a2 rs11614913	918	1175				
T vs C			51.5%	Random	0.857(0.704-1.043)	0.124
TT vs CC			59.3%	Random	0.757(0.480-1.194)	0.231
CT vs CC			56.9%	Random	0.961(0.654-1.413)	0.840

TT+CT vs CC			61.7%	Random	0.881(0.598-1.298)	0.523
TT vs CT+CC			0.0%	Fixed	0.730(0.598-0.890)	0.002
CC+TT vs CT			0.0%	Fixed	0.887(0.744-1.057)	0.180
Bsm1 rs1544410	581	965				
A vs G			0.0%	Fixed	1.243(1.067-1.447)	0.005
AA vs GG			0.0%	Fixed	1.387(0.985-1.952)	0.061
GA vs GG			0.0%	Fixed	1.440(1.151-1.801)	0.001
AA+GA vs GG			0.0%	Fixed	1.431(1.155-1.771)	0.001
AA vs GA+GG			0.0%	Fixed	1.148(0.835-1.578)	0.396
GG+AA vs GA			0.0%	Fixed	0.750(0.609-0.924)	0.007
Bsm1 rs1544410	1556	2200				
A vs C			0.0%	Fixed	1.005(0.912-1.108)	0.916
AA vs CC			0.0%	Fixed	0.971(0.794-1.187)	0.773
CA vs CC			0.0%	Fixed	1.073(0.926-1.244)	0.349
AA+CA vs CC			0.0%	Fixed	1.047(0.911-1.203)	0.515
AA vs			0.0%	Fixed		0.511

CA+CC					0.941(0.783-1.129)	
CC+AA vs CA			0.0%	Fixed	0.926(0.809-1.060)	0.264
Bsm1 rs1544410	571	601				
T vs C			20.0%	Fixed	1.014(0.857-1.198)	0.874
TT vs CC			0.0%	Fixed	1.026(0.713-1.477)	0.889
CT vs CC			75.0%	Random	0.908(0.449-1.835)	0.788
TT+CT vs CC			67.9%	Random	0.921(0.507-1.672)	0.787
TT vs CT+CC			0.0%	Fixed	0.921(0.662-1.280)	0.624
CC+TT vs CT			76.8%	Random	1.113(0.545-2.270)	0.769
Fok1 rs2228570	4086	6955				
T vs C			0.0%	Fixed	1.115(1.053-1.180)	<0.001
TT vs CC			0.0%	Fixed	1.224(1.087-1.378)	0.001
CT vs CC			36.5%	Fixed	1.138(1.044-1.240)	0.003
TT+CT vs CC			22.2%	Fixed	1.158(1.068-1.256)	<0.001
TT vs CT+CC			0.0%	Fixed	1.143(1.026-1.272)	0.015
CC+TT vs CT			41.8%	Fixed	0.935(0.864-1.011)	0.091
GSTP1 rs1695	328	632				
G vs A			0.0%	Fixed		0.793

					1.028(0.838-1.260)	
GG vs AA			0.0%	Fixed	1.443(0.880-2.367)	0.146
AG vs AA			40.3%	Fixed	0.945(0.686-1.301)	0.729
GG+AG vs AA			0.0%	Fixed	1.007(0.740-1.368)	0.967
GG vs AG+AA			79.5%	Random	1.192(0.516-2.750)	0.681
AA+GG vs AG			74.5%	Random	0.981(0.563-1.711)	0.947
VEGF rs3025039	434	572				
T vs C			0.0%	Fixed	0.960(0.749-1.232)	0.751
TT vs CC			0.0%	Fixed	1.093(0.444-2.692)	0.846
CT vs CC			65.8	Random	0.710(0.327-1.538)	0.385
TT+CT vs CC			47.7%	Fixed	0.901(0.666-1.218)	0.497
TT vs CT+CC			0.0%	Fixed	1.184(0.660-2.125)	0.570
CC+TT vs CT			66.6%	Random	1.306(0.732-2.328)	0.366
MLH1 rs1800734	1733	2063				
A vs G			96.6%	Random	1.050(0.613-1.798)	0.860
AA vs GG			94.3%	Random	1.180(0.470-2.960)	0.725
GA vs GG			89.1%	Random	0.963(0.572-1.6	0.866

					20)	
AA+GA vs GG			94.2%	Random	1.055(0.539-2.063)	0.876
AA vs GA+GG			92.7%	Random	1.138(0.600-2.160)	0.692
GG+AA vs GA			60.4%	Random	1.285(1.039-1.589)	0.021
MUC16 rs2547065	816	808				
G vs C			0.0%	Fixed	0.771(0.670-0.887)	<0.001
GG vs CC			0.0%	Fixed	0.575(0.430-0.770)	<0.001
CG vs CC			0.0%	Fixed	0.641(0.491-0.836)	0.001
GG+CG vs CC			0.0%	Fixed	0.612(0.477-0.787)	<0.001
GG vs CG+CC			0.0%	Fixed	0.792(0.641-0.978)	0.031
CC+GG vs CG			0.0%	Fixed	1.110(0.913-1.349)	0.296
MTHFR rs1801133	4143	4646				
T vs C			64.4%	Random	1.091(0.958-1.242)	0.189
TT vs CC			66.6%	Random	1.259(0.921-1.719)	0.148
CT vs CC			39.0%	Fixed	1.051(0.959-1.151)	0.286
TT+CT vs CC			52.3%	Random	1.083(0.932-1.258)	0.300
TT vs CT+CC			60.9%	Random	1.200(0.913-1.578)	0.192

CC+TT vs CT			13.9%	Fixed	0.974(0.893-1.062)	0.546
MTHFR rs1801131	3539	3747				
C vs A			21.6%	Fixed	1.099(1.023-1.181)	0.01
CC vs AA			10.4%	Fixed	1.200(1.018-1.416)	0.03
AC vs AA			0.3%	Fixed	1.104(0.998-1.220)	0.054
CC+AC vs AA			16.1%	Fixed	1.122(1.020-1.234)	0.018
CC vs AC+AA			0.0%	Fixed	1.148(0.980-1.344)	0.087
AA+CC vs AC			0.0%	Fixed	0.937(0.851-1.031)	0.180
MTR rs1805087	1774	1388				
G vs A			0.0%	Fixed	1.002(0.883-1.137)	0.975
GG vs AA			38.3%	Fixed	0.990(0.690-1.421)	0.958
AG vs AA			60.4%	Random	1.113(0.755-1.642)	0.589
GG+AG vs AA			21.4%	Fixed	1.005(0.866-1.166)	0.947
GG vs AG+AA			55.1%	Random	0.782(0.302-2.024)	0.612
AA+GG vs AG			69.1%	Random	0.872(0.559-1.361)	0.547
GALNT2 rs2271077	2413	2946				
T vs C			67.8%	Random		0.904

					1.020(0.737-1.413)	
TT vs CC			39.5%	Fixed	3.398(1.236-9.342)	0.018
CT vs CC			42.9%	Fixed	1.017(0.856-1.208)	0.850
TT+CT vs CC			58.2%	Random	1.000(0.742-1.347)	0.998
TT vs CT+CC			38.1%	Random	3.403(1.232-9.399)	0.018
CC+TT vs CT			39.8%	Fixed	0.989(0.832-1.175)	0.899
GALNT1 rs17647532	16417	19569				
C vs T			0.0%	Fixed	0.973(0.928-1.021)	0.269
CC vs TT			31.7%	Fixed	0.879(0.730-1.057)	0.171
TC vs TT			0.0%	Fixed	0.986(0.935-1.040)	0.609
CC+TC vs TT			0.0%	Fixed	0.978(0.929-1.031)	0.412
CC vs TC+TT			33.1%	Fixed	0.881(0.732-1.059)	0.177
TT+CC vs TC			0.0%	Fixed	1.012(0.960-1.068)	0.657
ESR1/SYNE1 rs2295190	6407	9316				
T vs G			0.0%	Fixed	1.112(1.044-1.185)	0.001
TT vs GG			19.8%	Fixed	1.162(0.933-1.447)	0.180

GT vs GG			0.0%	Fixed	1.129(1.049-1.215)	<0.001
TT+GT vs GG			0.0%	Fixed	1.131(1.053-1.215)	0.001
TT vs GT+GG			19.0%	Fixed	1.121(0.901-1.395)	0.305
GG+TT vs GT			0.0%	Fixed	0.890(0.827-0.957)	0.002
FUT3 rs2306969	827	939				
G vs A			0.0%	Fixed	0.913(0.781-1.068)	0.255
GG vs AA			7.2%	Fixed	0.913(0.594-1.404)	0.680
AG vs AA			0.0%	Fixed	0.878(0.721-1.070)	0.198
GG+AG vs AA			0.0%	Fixed	0.882(0.729-1.066)	0.194
GG vs AG+AA			6.5%	Fixed	0.960(0.629-1.465)	0.849
AA+GG vs AG			0.0%	Fixed	1.132(0.931-1.375)	0.214
GALNT6 rs907352	829	940				
C vs G			0.0%	Fixed	1.084(0.889-1.323)	0.426
CC vs GG			16.3%	Fixed	2.073(1.020-4.215)	0.044
GC vs GG			0.0%	Fixed	0.962(0.765-1.209)	0.737
CC+GC vs GG			0.0%	Fixed	1.023(0.820-1.277)	0.838
CC vs			14.8%	Fixed		0.041

GC+GG					2.092(1.031-4.247)	
GG+CC vs GC			0.0%	Fixed	1.059(0.843-1.331)	0.622
GALNT7 rs934358	829	940				
G vs C			0.0%	Fixed	0.819(0.697-0.961)	0.015
GG vs CC			0.0%	Fixed	0.567(0.350-0.920)	0.021
CG vs CC			0.0%	Fixed	0.855(0.701-1.043)	0.122
GG+CG vs CC			0.0%	Fixed	0.819(0.676-0.992)	0.041
GG vs CG+CC			8.3%	Fixed	0.601(0.373-0.969)	0.037
CC+GG vs CG			4.4%	Fixed	1.126(0.926-1.370)	0.235
MGAT5 rs1257187						
G vs A			0.0%	Fixed	1.230(1.026-1.475)	0.025
GG vs AA			0.0%	Fixed	2.293(1.190-4.416)	0.013
AG vs AA			0.0%	Fixed	1.135(0.919-1.403)	0.240
GG+AG vs AA			0.0%	Fixed	1.199(0.976-1.472)	0.084
GG vs AG+AA			0.0%	Fixed	2.214(1.153-4.255)	0.017
AA+GG vs AG			0.0%	Fixed	0.904 (0.732-1.116)	0.346
ST3GAL3 rs3828139	826	937				

C vs T			0.0%	Fixed	0.831(0.728-0.949)	0.006
CC vs TT			0.0%	Fixed	0.681(0.520-0.892)	0.005
TC vs TT			19.7%	Fixed	0.881 0.706- 1.100)	0.263
CC+TC vs TT			16.7%	Fixed	0.815(0.661-1.005)	0.056
CC vs TC+TT			0.0%	Fixed	0.740(0.589-0.929)	0.009
TT+CC vs TC			0.0%	Fixed	0.957(0.794-1.155)	0.649
ST3GAL3 rs37460	827	937				
C vs G			0.0%	Fixed	0.853(0.747-0.974)	0.019
CC vs GG			0.0%	Fixed	0.719(0.550-0.940)	0.016
GC vs GG			23.4%	Fixed	0.938(0.751-1.172)	0.575
CC+GC vs GG			17.8%	Fixed	0.864(0.701-1.066)	0.174
CC vs GC+GG			0.0%	Fixed	0.748(0.596-0.938)	0.012
GG+CC vs GC			0.0%	Fixed	0.918(0.761-1.107)	0.371
IL18 rs1834481	6925	8589				
C vs G			61.9%	Random	1.044(0.955-1.140)	0.343
CC vs GG			19.4%	Fixed	1.062(0.928-1.215)	0.380

GC vs GG			78.4%	Random	1.085(0.933-1.263)	0.289
CC+GC vs GG			76.5%	Random	1.076(0.937-1.237)	0.300
CC vs GC+GG			33.8%	Fixed	1.023(0.897-1.167)	0.730
GG+CC vs GC			79.6%	Random	0.922(0.791-1.073)	0.294
APAI rs7975232	869	1072				
A vs C			51.9%	Random	0.822(0.662-1.020)	0.075
AA vs CC			63.8%	Random	0.605(0.342-1.071)	0.085
CA vs CC			55.2%	Random	0.768(0.482-1.222)	0.265
AA+CA vs CC			63.7%	Random	0.698(0.425-1.146)	0.155
AA vs CA+CC			0.0%	Fixed	0.846(0.695-1.030)	0.096
CC+AA vs CA			0.0%	Fixed	0.935(0.781-1.120)	0.467
APAI rs7975232	168	320				
G vs T			0.0%	Fixed	0.794(0.608-1.036)	0.089
GG vs TT			0.0%	Fixed	0.623(0.366-1.060)	0.081
TG vs TT			0.0%	Fixed	0.829(0.537-1.279)	0.396
GG+TG vs TT			0.0%	Fixed	0.761(0.506-1.1	0.187

					42)	
GG vs TG+TT			34.9%	Fixed	0.718(0.455-1.134)	0.155
TT+GG vs TG			0.0%	Fixed	0.998(0.687-1.451)	0.992
TaqI rs731236	915	1248				
C vs T			0.0%	Fixed	1.048(0.920-1.193)	0.485
CC vs TT			0.0%	Fixed	1.086(0.825-1.430)	0.557
TC vs TT			0.0%	Fixed	1.097(0.904-1.331)	0.349
CC+TC vs TT			0.0%	Fixed	1.091(0.909-1.309)	0.350
CC vs TC+TT			0.0%	Fixed	1.008(0.784-1.296)	0.953
TT+CC vs TC			26.7%	Fixed	0.924(0.773-1.104)	0.384
SRD5A2 rs523349	1442	1791				
C vs G			0.0%	Fixed	1.307(1.175-1.454)	<0.001
CC vs GG			0.0%	Fixed	1.756 (1.393-2.213)	<0.001
GC vs GG			0.0%	Fixed	1.196(1.028-1.391)	0.021
CC+GC vs GG			0.0%	Fixed	1.305(1.132-1.504)	<0.001
CC vs GC+GG			0.0%	Fixed	1.625(1.305-2.024)	<0.001
GG+CC vs GC			13.5%	Fixed	0.939(0.813-1.084)	0.390
HSD17B4	1418	1774				

rs17145454						
C vs T			48.1%	Fixed	0.925(0.768-1.116)	0.417
CC vs TT			0.0%	Fixed	0.454(0.174-1.184)	0.106
TC vs TT			30.4%	Fixed	0.984(0.803-1.204)	0.872
CC+TC vs TT			42.3%	Fixed	0.952(0.781-1.161)	0.628
CC vs TC+TT			0.0%	Fixed	0.456(0.174-1.190)	0.109
TT+CC vs TC			27.5%	Fixed	1.011(0.826-1.237)	0.917
HSD17B1 rs605059	1354	1750				
G vs A			0.0%	Fixed	1.023(0.922-1.134)	0.670
GG vs AA			0.0%	Fixed	1.045(0.848-1.289)	0.678
AG vs AA			37.8%	Fixed	1.049(0.887-1.240)	0.578
GG+AG vs AA			0.0%	Fixed	1.046(0.893-1.226)	0.574
GG vs AG+AA			64.7%	Random	0.995(0.730-1.356)	0.976
AA+GG vs AG			73.4%	Random	0.951(0.714-1.265)	0.728
CYP19A1 rs10046	1438	1796				
T vs C			48.4%	Fixed	0.926(0.838-1.023)	0.130
TT vs CC			53.8%	Random	0.855(0.636-1.150)	0.301

CT vs CC			32.6%	Fixed	1.019(0.854-1.216)	0.835
TT+CT vs CC			50.8%	Random	0.954(0.752-1.210)	0.697
TT vs CT+CC			0.0%	Fixed	0.854 (0.728-1.002)	0.053
CC+TT vs CT			0.0%	Fixed	0.907(0.788-1.045)	0.178
BRCA2 rs144848	1422	1773				
C vs A			0.0%	Fixed	1.129(1.009-1.263)	0.035
CC vs AA			0.0%	Fixed	1.118(0.849-1.471)	0.427
AC vs AA			58.6%	Random	1.213(0.960-1.533)	0.106
CC+AC vs AA			19.6%	Fixed	1.207(1.046-1.391)	0.01
CC vs AC+AA			31.1%	Fixed	1.023(0.783-1.336)	0.869
AA+CC vs AC			68.8%	Random	0.839(0.645-1.091)	0.190
RB1 rs4151551	1486	4350				
T vs G			95.6%	Random	0.606(0.283-1.296)	0.196
TT vs GG			0.0%	Fixed	0.775(0.400-1.500)	0.449
GT vs GG			96.4%	Random	0.515(0.197-1.343)	0.175
TT+GT vs GG			96.4%	Random	0.518(0.204-1.320)	0.168
TT vs GT+GG			0.0%	Fixed		0.578

					0.832(0.434-1.593)	
GG+TT vs GT			96.4%	Random	1.917(0.743-4.948)	0.179
RB1 rs3092904	1332	4191				
A vs T			0.0%	Fixed	0.992(0.896-1.097)	0.871
AA vs TT			0.0%	Fixed	1.011(0.783-1.305)	0.935
TA vs TT			0.0%	Fixed	0.980(0.858-1.118)	0.760
AA+TA vs TT			0.0%	Fixed	0.983(0.867-1.116)	0.794
AA vs TA+TT			0.0%	Fixed	1.015(0.791-1.301)	0.908
TT+AA vs TA			8.6%	Fixed	1.022(0.898-1.163)	0.744
RB1 rs4151636	1481	4761				
G vs C			62.5%	Random	0.879(0.616-1.256)	0.479
GG vs CC			0.0%	Fixed	1.058(0.306-3.652)	0.929
CG vs CC			61.3%	Random	0.875(0.607-1.263)	0.476
GG+CG vs CC			62.7%	Random	0.874(0.604-1.264)	0.474
GG vs CG+CC			0.0%	Fixed	1.072(0.311-3.697)	0.913
CC+GG vs CG			61.0%	Random	1.142(0.792-1.6	0.477

					45)	
BRCA1 rs1799950	1349	2021				
G vs A			0.0%	Fixed	0.982(0.799-1.208)	0.866
GG vs AA			52.6%	Random	1.457(0.108-19.726)	0.777
AG vs AA			0.0%	Fixed	0.993(0.800-1.234)	0.953
GG+AG vs AA			0.0%	Fixed	0.988(0.796-1.225)	0.910
GG vs AG+AA			52.7%	Random	1.458(0.107-19.815)	0.777
AA+GG vs AG			0.0%	Fixed	1.006(0.810-1.250)	0.959
NBS1 rs1063045	1661	3582				
A vs G			0.0%	Fixed	0.980(0.897-1.070)	0.648
AA vs GG			0.0%	Fixed	0.933(0.765-1.138)	0.494
GA vs GG			14.6%	Fixed	1.006(0.889-1.139)	0.923
AA+GA vs GG			12.1%	Fixed	0.991(0.881-1.116)	0.887
AA vs GA+GG			0.0%	Fixed	0.931(0.771-1.124)	0.456
GG+AA vs GA			0.0%	Fixed	0.980(0.872-1.103)	0.742
NBS1 rs1805794	1586	2792				
G vs C			0.0%	Fixed	0.945(0.860-1.0	0.245

					39)	
GG vs CC			0.0%	Fixed	0.849(0.686-1.051)	0.133
CG vs CC			0.0%	Fixed	0.997(0.873-1.139)	0.964
GG+CG vs CC			0.0%	Fixed	0.965 (0.851-1.095)	0.584
GG vs CG+CC			0.0%	Fixed	0.848(0.693-1.039)	0.112
CC+GG vs CG			0.0%	Fixed	0.971(0.855-1.102)	0.644
NBS1 rs709816	1609	3899				
C vs T			0.0%	Fixed	0.970(0.886-1.061)	0.501
CC vs TT			0.0%	Fixed	0.929(0.768-1.124)	0.449
TC vs TT			0.0%	Fixed	0.987(0.864-1.128)	0.848
CC+TC vs TT			0.0%	Fixed	0.974(0.859-1.104)	0.675
CC vs TC+TT			0.0%	Fixed	0.937(0.785-1.117)	0.467
TT+CC vs TC			0.0%	Fixed	0.994(0.878-1.124)	0.920
NBS1 rs1061302	1561	3743				
G vs A			0.0%	Fixed	0.963(0.877-1.058)	0.435
GG vs AA			0.0%	Fixed	0.886(0.717-1.095)	0.264
AG vs AA			0.0%	Fixed	1.008(0.883-1.150)	0.912
GG+AG vs			0.0%	Fixed		0.782

AA					0.982(0.867-1.114)	
GG vs AG+AA			0.0%	Fixed	0.883(0.722-1.080)	0.225
AA+GG vs AG			0.0%	Fixed	0.969(0.855-1.098)	0.622
RAD51 rs1801321	1854	2812				
T vs G			0.0%	Fixed	1.029(0.945-1.120)	0.512
TT vs GG			0.0%	Fixed	1.069(0.899-1.271)	0.449
GT vs GG			0.0%	Fixed	1.010(0.883-1.156)	0.880
TT+GT vs GG			0.0%	Fixed	1.025(0.903-1.164)	0.699
TT vs GT+GG			0.0%	Fixed	1.057(0.907-1.231)	0.477
GG+TT vs GT			0.0%	Fixed	1.012(0.898-1.140)	0.849
RAD52 rs11226	1649	3906				
T vs C			56.1%	Random	1.016(0.890-1.160)	0.811
TT vs CC			55.7%	Random	1.017(0.779-1.327)	0.907
CT vs CC			35.7%	Fixed	1.123(0.976-1.293)	0.105
TT+CT vs CC			52.6%	Random	1.091(0.896-1.329)	0.384
TT vs CT+CC			28.6%	Fixed	0.922(0.795-1.070)	0.287
CC+TT vs CT			0.0%	Fixed	0.888(0.787-1.0	0.055

					02)	
XRCC2 rs3218536	4751	8273				
A vs G			96.3%	Random	1.058(0.662-1.691)	0.812
AA vs GG			83.4%	Random	1.126(0.468-2.709)	0.790
GA vs GG			81.0%	Random	0.808(0.622-1.051)	0.111
AA+GA vs GG			71.5%	Random	0.968(0.789-1.187)	0.753
AA vs GA+GG			92.0%	Random	1.079(0.366-3.177)	0.890
GG+AA vs GA			95.9%	Random	1.444(0.852-2.449)	0.172
XRCC3 rs1799794	1708	3919				
G vs A			0.0%	Fixed	1.006(0.905-1.120)	0.906
GG vs AA			0.0%	Fixed	0.780(0.560-1.088)	0.143
AG vs AA			0.0%	Fixed	1.088(0.955-1.238)	0.204
GG+AG vs AA			0.0%	Fixed	1.051(0.927-1.192)	0.433
GG vs AG+AA			0.0%	Fixed	0.768(0.554-1.063)	0.111
AA+GG vs AG			0.0%	Fixed	0.909(0.799-1.033)	0.143
XRCC3 rs1799796	1664	3966				
G vs A			0.0%	Fixed	1.003(0.916-1.098)	0.954
GG vs AA			0.0%	Fixed	1.118(0.918-1.3	0.267

					60)	
AG vs AA			0.0%	Fixed	0.894(0.786-1.016)	0.087
GG+AG vs AA			0.0%	Fixed	0.938(0.831-1.058)	0.296
GG vs AG+AA			0.0%	Fixed	1.183(0.982-1.426)	0.077
AA+GG vs AG			0.0%	Fixed	1.145(1.014-1.293)	0.030
XRCC3 rs861539	6048	10459				
T vs C			0.0%	Fixed	0.942(0.898-0.988)	0.014
TT vs CC			0.0%	Fixed	0.925(0.837-1.023)	0.131
CT vs CC			0.0%	Fixed	0.884(0.823-0.949)	0.001
TT+CT vs CC			0.0%	Fixed	0.894(0.836-0.956)	0.001
TT vs CT+CC			0.0%	Fixed	0.987(0.900-1.082)	0.779
CC+TT vs CT			0.0%	Fixed	1.104(1.034-1.178)	0.003
STK15 rs2273535	1394	1964				
A vs T			30.7%	Fixed	1.124(0.999-1.264)	0.052
AA vs TT			25.7%	Fixed	1.157(0.838-1.598)	0.375
TA vs TT			0.0%	Fixed	1.166(1.006-1.352)	0.042
AA+TA vs TT			0.0%	Fixed	1.166(1.011-1.3	0.034

					43)	
AA vs TA+TT			22.0%	Fixed	1.086(0.790-1.492)	0.611
TT+AA vs TA			0.0%	Fixed	0.865(0.748-1.001)	0.052
STK15 rs1047972	1505	2382				
A vs G			0.0%	Fixed	0.973(0.859-1.101)	0.663
AA vs GG			0.0%	Fixed	0.877(0.590-1.302)	0.514
GA vs GG			0.0%	Fixed	0.992(0.856-1.149)	0.915
AA+GA vs GG			0.0%	Fixed	0.981(0.851-1.131)	0.792
AA vs GA+GG			0.0%	Fixed	0.878(0.593-1.301)	0.518
GG+AA vs GA			0.0%	Fixed	1.002(0.866-1.160)	0.978
STK15 rs732417	1459	2202				
G vs C			43.9%	Fixed	1.126(0.944-1.343)	0.186
GG vs CC			32.8%	Fixed	1.271(0.542-2.983)	0.581
CG vs CC			0.0%	Fixed	1.125(0.930-1.361)	0.225
GG+CG vs CC			27.5%	Fixed	1.131(0.938-1.364)	0.196
GG vs CG+CC			30.3%	Fixed	1.253(0.533-2.941	0.605
CC+GG vs			0.0%	Fixed	0.890	0.230

CG					(0.736-1.077)	
STK15 rs8173	1401	2176				
C vs G			0.0%	Fixed	1.058(0.946-1.184)	0.322
CC vs GG			0.0%	Fixed	1.242(0.912-1.691)	0.168
GC vs GG			0.0%	Fixed	1.019(0.884-1.176)	0.793
CC+GC vs GG			0.0%	Fixed	1.044(0.910-1.198)	0.540
CC vs GC+GG			0.0%	Fixed	1.230(0.908-1.666)	0.181
GG+CC vs GC			0.0%	Fixed	0.999(0.868-1.149)	0.990
PGR rs10895068	2848	4305				
A vs G			37.4%	Fixed	1.036(0.883-1.216)	0.664
AA vs GG			0.0%	Fixed	1.674 (0.696-4.025)	0.250
GA vs GG			45.5%	Fixed	1.019(0.861-1.206)	0.829
AA+GA vs GG			42.9%	Fixed	1.028(0.870 -1.215)	0.744
AA vs GA+GG			0.0%	Fixed	1.666(0.694-4.000)	0.253
GG+AA vs GA			45.8%	Fixed	0.983(0.830-1.163)	0.840
HOTAIR rs920778	329	680				
T vs C			0.0%	Fixed	2.239(1.706-2.939)	<0.001
TT vs CC			0.0%	Fixed		0.001

					2.818(1.556-5.104)	
CT vs CC			36.0%	Fixed	2.177(1.523-3.111)	<0.001
TT+CT vs CC			0.0%	Fixed	2.316 (1.682-3.188)	<0.001
TT vs CT+CC			0.0%	Fixed	2.465(1.367-4.444)	0.003
CC+TT vs CT			40.3%	Fixed	0.490(0.344-0.698)	<0.001
HER2 rs1136201	321	518				
G vs A			86.9%	Random	1.456(0.671-3.160)	0.342
GG vs AA			77.5%	Random	3.122(0.459-21.248)	0.245
AG vs AA			41.9%	Fixed	0.994(0.698-1.415)	0.973
GG+AG vs AA			76.4%	Random	1.273(0.649-2.496)	0.483
GG vs AG+AA			75.5%	Random	3.183(0.512-19.802)	0.214
AA+GG vs AG			28.4%	Fixed	1.076(0.758-1.526)	0.682
ESR2 rs3020450	1175	1433				
A vs G			0.0%	Fixed	0.797(0.702-0.904)	<0.001
AA vs GG			29.5%	Fixed	0.777(0.570-1.058)	0.109
GA vs GG			0.0%	Fixed	0.711(0.602-0.840)	<0.001
AA+GA vs GG			0.0%	Fixed	0.723(0.617-0.847)	<0.001
AA vs GA+GG			36.5%	Fixed	0.898(0.665-1.2	0.483

					12)	
GG+AA vs GA			0.0%	Fixed	1.364(1.159-1.605)	<0.001
MTRR rs1801394	1772	1387				
G vs A			0.0%	Fixed	1.020(0.923-1.128)	0.696
GG vs AA			0.0%	Fixed	1.046(0.855-1.279)	0.662
AG vs AA			44.2%	Fixed	0.966(0.822-1.136)	0.679
GG+AG vs AA			0.0%	Fixed	0.990(0.851-1.153)	0.901
GG vs AG+AA			29.0%	Fixed	1.076(0.903-1.283)	0.410
AA+GG vs AG			66.7%	Random	0.942(0.629-1.411)	0.771
Cdx-2 rs11568820	1567	2226				
A vs G			21.0%	Fixed	1.092(0.976-1.222)	0.124
AA vs GG			0.0%	Fixed	1.001(0.743-1.348)	0.997
GA vs GG			64.6%	Random	1.019(0.751-1.383)	0.904
AA+GA vs GG			59.3%	Random	1.040(0.794-1.363)	0.775
AA vs GA+GG			0.0%	Fixed	1.008(0.754-1.348)	0.955
GG+AA vs GA			61.5%	Random	0.974(0.734-1.292)	0.853
VDR	580	607				

rs2239179						
C vs T			0.0%	Fixed	1.078(0.915-1.270)	0.368
CC vs TT			0.0%	Fixed	1.110(0.782-1.574)	0.559
TC vs TT			0.0%	Fixed	1.229(0.949-1.591)	0.117
CC+TC vs TT			0.0%	Fixed	1.200(0.938-1.534)	0.147
CC vs TC+TT			0.0%	Fixed	0.981(0.721-1.334)	0.901
TT+CC vs TC			0.0%	Fixed	0.846(0.673-1.062)	0.150
VDR rs3782905	581	605				
C vs G			9.6%	Fixed	1.089(0.915-1.296)	0.337
CC vs GG			0.0%	Fixed	1.157(0.774-1.731)	0.477
GC vs GG			0.0%	Fixed	1.126 (0.884-1.434)	0.336
CC+GC vs GG			0.0%	Fixed	1.127(0.896-1.419)	0.307
CC vs GC+GG			0.0%	Fixed	1.085(0.739-1.593)	0.677
GG+CC vs GC			0.0%	Fixed	0.913(0.725-1.150)	0.439
LINC02354 rs7968585	582	609				
G vs A			0.0%	Fixed	0.991(0.844-1.1	0.917

					65)	
GG vs AA			49.9%	Fixed	1.006(0.727-1.392)	0.971
AG vs AA			43.4%	Fixed	1.051(0.802-1.377)	0.718
GG+AG vs AA			0.0%	Fixed	1.030(0.798-1.329)	0.821
GG vs AG+AA			79.1%	Random	1.356(0.461-3.988)	0.580
AA+GG vs AG			78.9%	Random	1.173(0.561-2.455)	0.671
MDM2 rs2279744	1908	2830				
G vs T			84.3%	Random	0.930(0.666-1.300)	0.673
GG vs TT			79.5%	Random	0.846(0.464-1.542)	0.586
TG vs TT			78.5%	Random	0.906(0.552-1.486)	0.695
GG+TG vs TT			83.6%	Random	0.872(0.511-1.489)	0.616
GG vs TG+TT			56.1%	Random	0.929(0.666-1.296)	0.664
TT+GG vs TG			53.4%	Random	0.949(0.727-1.238)	0.699
IL-18 rs187238	267	377				
C vs G			0.0%	Fixed	0.798(0.608-1.048)	0.104
CC vs GG			0.0%	Fixed	0.605(0.295-1.240)	0.170
GC vs GG			0.0%	Fixed	0.845(0.599-1.1	0.338

					92)	
CC+GC vs GG			0.0%	Fixed	0.803(0.579-1.115)	0.191
CC vs GC+GG			10.9%	Fixed	0.619(0.307-1.249)	0.180
GG+CC vs GC			40.3%	Fixed	1.121(0.799-1.571)	0.509
VEGF rs833061	350	409				
C vs T			0.0%	Fixed	0.851(0.666-1.088)	0.198
CC vs TT			0.0%	Fixed	0.668(0.353-1.267)	0.217
TC vs TT			13.9%	Fixed	0.870(0.636-1.192)	0.387
CC+TC vs TT			0.0%	Fixed	0.843(0.624-1.138)	0.263
CC vs TC+TT			0.0%	Fixed	0.742(0.398-1.384)	0.348
TT+CC vs TC			0.0%	Fixed	1.111(0.817-1.511)	0.503
IL-10 rs1800896	180	219				
G vs A			0.0%	Fixed	0.958(0.699-1.313)	0.789
GG vs AA			0.0%	Fixed	0.913(0.469-1.779)	0.790
AG vs AA			0.0%	Fixed	0.970(0.579-1.625)	0.909
GG+AG vs AA			0.0%	Fixed	0.960 (0.593-1.553)	0.867
GG vs AG+AA			0.0%	Fixed	0.918 (0.513-1.642)	0.772
AA+GG vs			0.0%	Fixed		0.947

AG					0.985(0.629-1.543)	
IL-10 rs1800871	411	438				
C vs T			72.6%	Random	0.645(0.404-1.030)	0.066
CC vs TT			57.5%	Random	0.310(0.126-0.766)	0.011
TC vs TT			84.1%	Random	0.882(0.298-2.612)	0.820
CC+TC vs TT			84.0%	Random	0.643(0.236-1.751)	0.388
CC vs TC+TT			0.0%	Fixed	0.439(0.301-0.638)	<0.001
TT+CC vs TC			60.7%	Random	0.707(0.421-1.187)	0.189
Fas rs2234767	389	385				
A vs G			3.8%	Fixed	0.995(0.800-1.237)	0.963
AA vs GG			——	——	——	——
GA vs GG			12.1%	Fixed	1.040(0.773-1.399)	0.797
AA+GA vs GG			17.9%	Fixed	1.021(0.768-1.356)	0.887
AA vs GA+GG			——	——	——	——
GG+AA vs GA			6.6%	Fixed	0.951(0.715-1.265)	0.729
FasL rs763110	389	385				
C vs T			0.0%	Fixed	1.422(1.132-1.787)	0.002
CC vs TT			0.0%	Fixed		0.015

					2.080(1.155-3.745)	
TC vs TT			0.0%	Fixed	1.180(0.673-2.071)	0.564
CC+TC vs TT			0.0%	Fixed	1.534(0.898-2.618)	0.117
CC vs TC+TT			39.5	Fixed	1.555(1.163-2.079)	0.003
TT+CC vs TC			0.0%	Fixed	1.372(1.026 -1.835)	0.033
ERCC2(XPD) rs1799793	657	2017				
A vs G			29.3%	Fixed	1.120(0.957 -1.310)	0.157
AA vs GG			57.1%	Random	1.191(0.660-2.149)	0.562
GA vs GG			0.0%	Fixed	1.125(0.904-1.402)	0.291
AA+GA vs GG			0.0%	Fixed	1.149(0.932-1.417)	0.193
AA vs GA+GG			56.5%	Random	1.175(0.682-2.024)	0.561
GG+AA vs GA			22.8%	Fixed	0.926(0.753-1.139)	0.468
H19 rs2107425	556	629				
T vs C			81.1%	Random	0.917(0.804-1.045)	0.195
TT vs CC			13.7%	Fixed	0.984(0.886-1.092)	0.755
CT vs CC			88.8%	Random	0.823(0.652-1.038)	0.100
TT+CT vs CC			88.2%	Random	0.847(0.685-1.0	0.125

					47)	
TT vs CT+CC			0.0%	Fixed	1.047(0.948-1.157)	0.365
CC+TT vs CT			87.3%	Random	1.204(0.977-1.484)	0.082
EPHX1 rs1051740	1919	1829				
C vs T			49.4%	Fixed	1.021(0.925-1.128)	0.675
CC vs TT			0.0%	Fixed	1.083(0.878-1.337)	0.456
TC vs TT			55.2%	Random	0.943(0.733-1.213)	0.650
CC+TC vs TT			54.1%	Random	0.950(0.756-1.193)	0.658
CC vs TC+TT			0.0%	Fixed	1.079(0.884-1.316)	0.455
TT+CC vs TC			51.8%	Random	1.045(0.832-1.312)	0.706
CDKN2A rs3731249	556	629				
T vs A			0.0%	Fixed	0.812(0.631-1.046)	0.107
TTvs AA			——	——	——	——
AT vs AA			0.0%	Fixed	0.789(0.606-1.026)	0.077
TT+AT vs AA			0.0%	Fixed	0.798(0.616-1.035)	0.089
TT vs AT+AA			——	——	——	——
AA+TT vs AT			0.0%	Fixed	1.268(0.975-1.650)	0.077
p16/CDKN2 rs11515	2737	4036				
G vs C			0.0%	Fixed		0.671

					1.022(0.925-1.130)	
GG vs CC			0.0%	Fixed	1.604(1.131-2.275)	0.008
CG vs CC			0.0%	Fixed	0.938(0.835-1.053)	0.278
GG+CG vs CC			0.0%	Fixed	0.977(0.873-1.094)	0.689
GG vs CG+CC			0.0%	Fixed	1.627(1.148-2.304)	0.006
CC+GG vs CG			0.0%	Fixed	1.081(0.962-1.213)	0.190
p16/CDKN2 rs3088440	556	629				
T vs C			29.5%	Fixed	1.058(0.916-1.222)	0.443
TT vs CC			47.7%	Fixed	1.249(0.646-2.416)	0.509
CT vs CC			0.0%	Fixed	1.047(0.894-1.226)	0.568
TT+CT vs CC			0.0%	Fixed	1.056(0.904-1.232)	0.495
TT vs CT+CC			46.2%	Fixed	1.239(0.641-2.397)	0.524
CC+TT vs CT			0.0%	Fixed	0.957(0.817-1.120)	0.582
CDKN1B rs2066827	1829	2868				
G vs T			83.8%	Random	0.969(0.585-1.608)	0.904
GG vs TT			79.0%	Random	1.245(0.462-3.354)	0.665

TG vs TT			56.0%	Random	0.798(0.508-1.254)	0.327
GG+TG vs TT			68.7%	Random	0.882(0.533-1.457)	0.623
GG vs TG+TT			80.8%	Random	1.214(0.518-2.847)	0.655
TT+GG vs TG			52.4%	Random	1.345(0.927-1.952)	0.119
MDM2 rs117039649						
C vs G			0.0%	Fixed	0.767(0.632-0.931)	0.007
CC vs GG			——	——	——	——
GC vs GG			0.0%	Fixed	0.769(0.629-0.941)	0.011
CC+GC vs GG			0.0%	Fixed	0.764(0.626-0.933)	0.008
CC vs GC+GG			——	——	——	——
GG+CC vs GC			0.0%	Fixed	1.299(1.062-1.589)	0.011
PPAR-γ/PPARG rs1801282	1384	2137				
G vs C			0.0%	Fixed	1.014(0.870-1.183)	0.857
GG vs CC			0.0%	Fixed	0.984(0.536-1.809)	0.960
CG vs CC			0.0%	Fixed	1.029(0.866-1.223)	0.747
GG+CG vs CC			0.0%	Fixed	1.023(0.864-1.211)	0.792
GG vs CG+CC			0.0%	Fixed	0.979(0.534-1.797)	0.946

CC+GG vs CG			0.0%	Fixed	0.971(0.817-1.154)	0.737
COX-2 rs20417	556	629				
C vs G			0.0%	Fixed	0.881(0.765-1.014)	0.078
CC vs GG			55.2%	Random	0.921(0.432-1.961)	0.831
GC vs GG			0.0%	Fixed	0.878(0.742-1.040)	0.131
CC+GC vs GG			0.0%	Fixed	0.870 (0.740-1.023)	0.091
CC vs GC+GG			54.3%	Random	0.951(0.452-2.000)	0.894
GG+CC vs GC			0.0%	Fixed	1.127(0.953-1.333)	0.162
COX-2/PTGS2 rs5275	2288	3402				
C vs T			0.0%	Fixed	0.973(0.897-1.056)	0.517
CC vs TT			0.0%	Fixed	0.914(0.763-1.095)	0.329
TC vs TT			0.0%	Fixed	1.010(0.902-1.132)	0.858
CC+TC vs TT			0.0%	Fixed	0.990(0.889-1.102)	0.850
CC vs TC+TT			0.0%	Fixed	0.911(0.768-1.082)	0.290
TT+CC vs TC			0.0%	Fixed	0.973(0.873-1.085)	0.628
COMT rs4680	1439	2927				
G vs A			0.8%	Fixed	0.996(0.905-1.096)	0.932

GG vs AA			1.0%	Fixed	1.001(0.827-1.212)	0.991
AG vs AA			0.0%	Fixed	0.944(0.797-1.117)	0.5
GG+AG vs AA			0.0%	Fixed	0.963(0.822-1.128)	0.642
GG vs AG+AA			0.0%	Fixed	1.025(0.879-1.194)	0.755
AA+GG vs AG			0.0%	Fixed	1.048(0.915-1.199)	0.501
PGR rs1042839	1740	1874				
T vs G			75.5%	Random	1.017(0.750-1.379)	0.915
TT vs GG			33.2%	Fixed	1.078(0.693-1.677)	0.739
GT vs GG			80.1%	Random	0.959(0.647-1.423)	0.836
TT+GT vs GG			78.9%	Random	0.987(0.680-1.433)	0.946
TT vs GT+GG			31.7%	Fixed	1.156(0.745-1.793)	0.517
GG+TT vs GT			80.1%	Random	1.052(0.710-1.557)	0.801
PGR rs1042838	2786	2657				
T vs G			69.0%	Random	0.940(0.767-1.151)	0.550
TT vs GG			52.8%	Random	0.925(0.509-1.6	0.799

					83)	
GT vs GG			66.8%	Random	1.013(0.800-1.284)	0.912
TT+GT vs GG			62.0%	Random	0.965(0.781-1.191)	0.740
TT vs GT+GG			54.8%	Random	0.928(0.505-1.705)	0.809
GG+TT vs GT			69.4%	Random	0.972(0.761-1.242)	0.823
CYP1A2 rs762551	556	629				
C vs A			94.7%	Random	1.615(0.524-4.975)	0.404
CC vs AA			91.7%	Random	3.200(0.151-68.038)	0.456
AC vs AA			91.4%	Random	1.836(0.565-5.972)	0.312
CC+AC vs AA			93.9%	Random	1.894(0.488-7.346)	0.356
CC vs AC+AA			88.1%	Random	2.358(0.193-28.762)	0.502
AA+CC vs AC			86.8%	Random	0.597(0.239-1.493)	0.270
CYP1B1 rs1056836	1199	2596				
G vs C			57.7%	Random	0.959(0.796-1.156)	0.660
GG vs CC			41.8%	Fixed	0.837(0.672-1.043)	0.112
CG vs CC			32.0%	Fixed	1.032(0.870-1.225)	0.715
GG+CG vs CC			46.9%	Fixed	0.988(0.843-1.159)	0.886

GG vs CG+CC			19.8%	Fixed	0.823(0.678-0.998)	0.048
CC+GG vs CG			0.0%	Fixed	0.895(0.770-1.041)	0.152
CYP1B1 rs1056827	799	1172				
T vs G			0.0%	Fixed	1.064(0.926-1.224)	0.381
TT vs GG			0.0%	Fixed	1.102(0.798-1.522)	0.556
GT vs GG			0.0%	Fixed	1.083(0.892-1.315)	0.418
TT+GT vs GG			0.0%	Fixed	1.088(0.906-1.307)	0.367
TT vs GT+GG			0.0%	Fixed	1.068(0.784-1.454)	0.677
GG+TT vs GT			0.0%	Fixed	0.939(0.779-1.131)	0.505
CYP1B1 rs1800440	361	1577				
G vs A			0.0%	Fixed	1.147(0.909-1.448)	0.247
GG vs AA			0.0%	Fixed	1.061(0.511-2.205)	0.873
AG vs AA			0.0%	Fixed	1.230(0.931-1.625)	0.144
GG+AG vs AA			0.0%	Fixed	1.209(0.923-1.584)	0.168
GG vs AG+AA			0.0%	Fixed	0.999(0.484-2.064)	0.998
AA+GG vs AG			0.0%	Fixed	0.814(0.617-1.074)	0.145
