# Supplementary material for: The association between single nucleotide polymorphisms and ovarian cancer risk: A systematic review and network meta‐analysis
Source: Cancer Med. 2022 May 30;12(1):541–56. doi: 10.1002/cam4.4891 (PMC9844622; doi:10.1002/cam4.4891)
Supplement: Supplementary file 6 — Supplement Information S6 [file CAM4-12-541-s006.pdf]

Supplement information 6. Several networks of intergene associations.

Table 1. A network of intergene associations.

A1	1.2 (0.35, 4.14)	2.00(0.59, 6.75)	3.41 (1, 11.77)	1.17 (0.09, 15.82)	3.17 (0.24, 42.97)	2.28 (0.17, 30.99)	2.17 (0.17, 29.48)	2.07 (0.15, 27.25)	4.16 (0.31, 56.56)	1.98 (0.04, 99.39)	2.2 (0.04, 114.02)	2.29 (0.05, 113.23)	0.07 (0, 4.07)	0.76 (0.01, 40.15)	1.6 (0.33, 7.8)	1.98 (0.04, 97.01)	2.33 (0.05, 115.42)	0.25 (0.01, 12.23)	3.39 (0.29, 39.09)	6.10 (0.51, 70.97)	1.58 (0.14, 19.08)	13.36 (1.13, 166.34)	0.35 (0.03, 4.18)	1.46 (0.12, 17.83)	1.37 (0.12, 16.5)	0.87 (0.14, 5.34)	0.13 (0.02, 0.82)
0.83 (0.24, 2.83)	A3	1.65 (0.49, 5.62)	2.83 (0.85, 9.71)	0.97 (0.07, 12.88)	2.61 (0.2, 35.84)	1.9 (0.14, 25.97)	1.8 (0.14, 24.07)	1.71 (0.13, 23.69)	3.44 (0.26, 45.8)	1.65 (0.03, 83.43)	1.83 (0.04, 95.51)	1.91 (0.04, 95.69)	0.06 (0, 3.25)	0.64 (0.01, 34.25)	1.33 (0.29, 6.47)	1.65 (0.04, 79.56)	1.94 (0.04, 98.25)	0.21 (0, 10.06)	2.81 (0.24, 33.11)	5.02 (0.43, 58.61)	1.31 (0.11, 15.76)	10.99 (0.94, 134.62)	0.29 (0.03, 3.48)	1.21 (0.11, 14.52)	1.13 (0.1, 13.41)	0.72 (0.12, 4.35)	0.1 (0.02, 0.68)
0.50 (0.15, 1.71)	0.61 (0.18, 2.02)	A4	1.72 (0.5, 5.8)	0.59 (0.04, 7.6)	1.58 (0.12, 21.38)	1.15 (0.09, 15.45)	1.09 (0.08, 14.25)	1.04 (0.08, 14.06)	2.09 (0.16, 27.74)	1.00 (0.02, 51.09)	1.11 (0.02, 57.06)	1.16 (0.02, 57.13)	0.04 (0, 2.01)	0.39 (0.01, 20.94)	0.81 (0.17, 3.86)	1.00 (0.02, 48.35)	1.17 (0.03, 58.15)	0.13 (0, 6.13)	1.69 (0.14, 20.06)	3.04 (0.26, 36.42)	0.79 (0.07, 9.61)	6.73 (0.54, 81.09)	0.18 (0.02, 2.09)	0.73 (0.06, 8.71)	0.69 (0.06, 8.38)	0.44 (0.07, 2.67)	0.06 (0.01, 0.41)
0.29 (0.08, 1)	0.35 (0.1, 1.18)	0.58 (0.17, 1.98)	A6	0.34 (0.03, 4.53)	0.93 (0.07, 12.45)	0.67 (0.05, 9)	0.64 (0.05, 8.59)	0.6 (0.04, 8.2)	1.21 (0.09, 16.24)	0.58 (0.01, 29.76)	0.65 (0.01, 32.49)	0.67 (0.01, 33.94)	0.02 (0, 1.19)	0.22 (0, 12.03)	0.47 (0.1, 2.25)	0.58 (0.01, 28.15)	0.69 (0.02, 33.85)	0.07 (0, 3.63)	0.99 (0.09, 11.56)	1.76 (0.15, 20.83)	0.46 (0.04, 5.5)	3.91 (0.32, 47.71)	0.1 (0.01, 1.19)	0.43 (0.04, 5.23)	0.4 (0.03, 4.83)	0.25 (0.04, 1.55)	0.04 (0.01, 0.24)
0.85 (0.06, 11.29)	1.03 (0.08, 13.55)	1.7 (0.13, 22.33)	2.92 (0.22, 39.11)	E1	2.71 (0.24, 29.83)	1.93 (0.18, 21.54)	1.86 (0.17, 20.62)	1.77 (0.15, 18.97)	3.53 (0.31, 39.49)	1.70(0.02, 169.26)	1.88 (0.02, 182.25)	1.95 (0.02, 180.52)	0.06 (0, 6.22)	0.66 (0.01, 65.72)	1.37 (0.08, 22.65)	1.72 (0.02, 156.19)	2.03 (0.02, 183.36)	0.22 (0, 19.89)	2.87 (0.1, 87.17)	5.10 (0.17, 158.98)	1.33 (0.04, 41.75)	11.5 (0.38, 335.57)	0.3 (0.01, 9.1)	1.25 (0.04, 38.61)	1.19 (0.04, 35.34)	0.74 (0.04, 14.62)	0.11 (0.01, 2.15)
0.32 (0.02, 4.2)	0.38 (0.03, 4.95)	0.63 (0.05, 8.24)	1.08 (0.08, 14.48)	0.37 (0.03, 4.1)	E2	0.72 (0.06, 7.95)	0.69 (0.06, 7.8)	0.66 (0.06, 7.18)	1.31 (0.12, 14.52)	0.63 (0.01, 63.42)	0.7 (0.01, 69.65)	0.73 (0.01, 70.91)	0.02 (0, 2.4)	0.24 (0, 25.48)	0.5 (0.03, 8.83)	0.63 (0.01, 59.84)	0.74 (0.01, 72.54)	0.08 (0, 7.72)	1.07 (0.03, 31.42)	1.9 (0.06, 59.23)	0.49 (0.02, 15)	4.27 (0.14, 126.67)	0.11 (0, 3.26)	0.46 (0.02, 13.93)	0.43 (0.01, 12.88)	0.27 (0.01, 5.35)	0.04 (0, 0.82)

0.44 (0.01, 20.76)	0.45 (0.01, 22.78)	0.51 (0.01, 24.85)	0.24 (0.02, 3.2)	0.48 (0.04, 6.47)	0.46 (0.03, 6.03)	0.44 (0.03, 5.92)
0.52 (0.01, 24.55)	0.55 (0.01, 26.88)	0.61 (0.01, 30)	0.29 (0.02, 3.89)	0.59 (0.04, 7.61)	0.56 (0.04, 7.13)	0.53 (0.04, 7.12)
0.86 (0.02, 41.29)	0.90 (0.02, 43.43)	1.00 (0.02, 48.84)	0.48 (0.04, 6.43)	0.96 (0.07, 13.17)	0.91 (0.07, 12.06)	0.87 (0.06, 11.55)
1.50 (0.03, 70.75)	1.54 (0.03, 74.73)	1.72 (0.03, 83.86)	0.83 (0.06, 11.29)	1.66 (0.12, 22.46)	1.57 (0.12, 21.02)	1.49 (0.11, 20.21)
0.51 (0.01, 47.36)	0.53 (0.01, 50.72)	0.59 (0.01, 56.18)	0.28 (0.03, 3.2)	0.57 (0.05, 6.48)	0.54 (0.05, 6)	0.52 (0.05, 5.71)
1.38 (0.01, 123.9)	1.43 (0.01, 137.62)	1.59 (0.02, 146.44)	0.76 (0.07, 8.6)	1.53 (0.14, 17.11)	1.45 (0.13, 16.43)	1.38 (0.13, 15.64)
0.99 (0.01, 92.73)	1.03 (0.01, 97.14)	1.14 (0.01, 108.7)	0.55 (0.05, 6.37)	1.1 (0.1, 12.56)	1.06 (0.09, 11.85)	E3
0.93 (0.01, 85.63)	0.99 (0.01, 92.88)	1.1 (0.01, 101.78)	0.52 (0.05, 6.11)	1.05 (0.1, 11.93)	E4	0.95 (0.08, 10.89)
0.9 (0.01, 81.38)	0.93 (0.01, 87.36)	1.04 (0.01, 98.11)	0.5 (0.04, 5.47)	E5	0.95 (0.08, 10.33)	0.91 (0.08, 10.2)
1.79 (0.02, 171.3)	1.87 (0.02, 176.33)	2.09 (0.02, 200.67)	E6	1.99 (0.18, 22.73)	1.92 (0.16, 21.45)	1.82 (0.16, 21.21)
0.87 (0.08, 9.69)	0.9 (0.08, 10.18)	B1	0.48 (0, 48.07)	0.96 (0.01, 95.56)	0.91 (0.01, 90.79)	0.88 (0.01, 90.68)
0.96 (0.08, 10.52)	B2	1.11 (0.1, 12.34)	0.53 (0.01, 52.54)	1.08 (0.01, 103.55)	1.01 (0.01, 100.44)	0.98 (0.01, 94.71)
B3	1.05 (0.1, 11.77)	1.16 (0.1, 12.69)	0.56 (0.01, 54.22)	1.11 (0.01, 108.79)	1.07 (0.01, 103.01)	1.01 (0.01, 98.4)
0.03 (0, 0.4)	0.03 (0, 0.43)	0.04 (0, 0.46)	0.02 (0, 1.88)	0.04 (0, 3.8)	0.03 (0, 3.46)	0.03 (0, 3.35)
0.33 (0.03, 4.06)	0.35 (0.03, 4.17)	0.38 (0.03, 4.68)	0.19 (0, 19.06)	0.37 (0, 37.83)	0.35 (0, 35.72)	0.34 (0, 34.32)
0.7 (0.02, 24.83)	0.72 (0.02, 26.66)	0.81 (0.02, 29.38)	0.39 (0.02, 6.95)	0.78 (0.05, 13.21)	0.73 (0.04, 12.82)	0.7 (0.04, 12.34)
0.86 (0.01, 133.14)	0.91 (0.01, 140.59)	0.99 (0.01, 160.27)	0.48 (0.01, 46.83)	0.98 (0.01, 86.77)	0.92 (0.01, 85.46)	0.88 (0.01, 83.88)
1.01 (0.01, 160.83)	1.06 (0.01, 171.37)	1.18 (0.01, 185.74)	0.57 (0.01, 53.99)	1.15 (0.01, 105.17)	1.07 (0.01, 103.23)	1.02 (0.01, 97.8)
0.11 (0, 17.29)	0.12 (0, 17.73)	0.13 (0, 20.24)	0.06 (0, 6)	0.12 (0, 11.51)	0.12 (0, 11.67)	0.11 (0, 10.46)
1.45 (0.02, 129.9)	1.5 (0.02, 135.75)	1.69 (0.02, 149.15)	0.82 (0.03, 25.11)	1.64 (0.05, 50.11)	1.57 (0.05, 47.43)	1.48 (0.05, 45.6)
2.62 (0.03, 229.27)	2.71 (0.03, 251.31)	3.02 (0.03, 273.76)	1.47 (0.05, 45.89)	2.87 (0.1, 90.66)	2.77 (0.09, 83.97)	2.64 (0.09, 78.88)
0.68 (0.01, 59.79)	0.70 (0.01, 67.31)	0.80 (0.01, 71)	0.38 (0.01, 12)	0.76 (0.03, 23.71)	0.72 (0.02, 22.04)	0.68 (0.02, 22.05)
5.93 (0.06, 514.56)	6.06 (0.07, 542.12)	6.78 (0.07, 603.96)	3.23 (0.1, 98.53)	6.46 (0.21, 200.14)	6.18 (0.2, 191.79)	5.85 (0.19, 183.78)
0.15 (0, 13.48)	0.16 (0, 14.35)	0.18 (0, 15.67)	0.09 (0, 2.54)	0.17 (0.01, 5.29)	0.16 (0.01, 4.69)	0.15 (0, 4.84)
0.64 (0.01, 57.37)	0.65 (0.01, 60.01)	0.74 (0.01, 64.78)	0.35 (0.01, 10.94)	0.71 (0.02, 21.88)	0.67 (0.02, 20.39)	0.64 (0.02, 20)
0.6 (0.01, 51.94)	0.62 (0.01, 55.09)	0.69 (0.01, 61.7)	0.33 (0.01, 10.35)	0.67 (0.02, 20.18)	0.63 (0.02, 18.91)	0.6 (0.02, 18.32)
0.37 (0.01, 21.7)	0.39 (0.01, 23.89)	0.44 (0.01, 26.01)	0.21 (0.01, 4.19)	0.42 (0.02, 8.48)	0.4 (0.02, 7.87)	0.38 (0.02, 7.56)
0.06 (0, 3.38)	0.06 (0, 3.45)	0.06 (0, 3.88)	0.03 (0, 0.62)	0.06 (0, 1.26)	0.06 (0, 1.16)	0.06 (0, 1.12)

0.30 (0.03, 3.45)	3.92 (0.08, 187.55)	0.43 (0.01, 19.79)	0.51 (0.01, 23.78)	0.62 (0.13, 2.99)	1.31 (0.02, 67.25)	13.46 (0.25, 708.29)
0.36 (0.03, 4.13)	4.76 (0.1, 225.96)	0.52 (0.01, 24.07)	0.61 (0.01, 27.6)	0.75 (0.15, 3.49)	1.57 (0.03, 80.08)	16.29 (0.31, 845.72)
0.59 (0.05, 6.92)	7.81 (0.16, 375.33)	0.86 (0.02, 39.15)	1.00(0.02, 46.14)	1.24 (0.26, 5.88)	2.59 (0.05, 130.64)	26.77 (0.5, 1410.25)
1.01 (0.09, 11.68)	13.46 (0.28, 632.42)	1.45 (0.03, 66.32)	1.72 (0.04, 77.58)	2.13 (0.44, 10.26)	4.47 (0.08, 226.88)	45.87 (0.84, 2389.67)
0.35 (0.01, 10.38)	4.58 (0.05, 415.98)	0.49 (0.01, 44.39)	0.58 (0.01, 52.53)	0.73 (0.04, 12.42)	1.52 (0.02, 146.16)	15.79 (0.16, 1565.71)
0.94 (0.03, 28.64)	12.56 (0.13, 1125.44)	1.34 (0.01, 122.58)	1.59 (0.02, 141.39)	2.00 (0.11, 33.79)	4.12 (0.04, 404.3)	42.97 (0.42, 4187.54)
0.68 (0.02, 20.4)	9.03 (0.1, 800.18)	0.98 (0.01, 86.96)	1.14 (0.01, 104)	1.43 (0.08, 25.03)	2.96 (0.03, 296.54)	31 (0.3, 3074)
0.64 (0.02, 19.68)	8.58 (0.09, 776.76)	0.93 (0.01, 84.27)	1.09 (0.01, 99.15)	1.36 (0.08, 23.3)	2.83 (0.03, 268.49)	29.15 (0.29, 2962.39)
0.61 (0.02, 18.79)	8.12 (0.09, 709.03)	0.87 (0.01, 79.96)	1.02 (0.01, 93.68)	1.29 (0.08, 21.92)	2.73 (0.03, 261.22)	27.85 (0.26, 2726.09)
1.21 (0.04, 38.6)	16.53 (0.17, 1456.82)	1.77 (0.02, 162.34)	2.07 (0.02, 187.31)	2.58 (0.14, 44.43)	5.37 (0.05, 527.08)	55.64 (0.53, 5441.81)
0.59 (0.01, 56.59)	7.87 (0.05, 1243.61)	0.85 (0.01, 133.93)	1.01 (0.01, 150.83)	1.24 (0.03, 44.21)	2.6 (0.21, 31.14)	26.8 (2.16, 338.57)
0.67 (0.01, 58.2)	8.67 (0.06, 1359.96)	0.94 (0.01, 151.45)	1.1 (0.01, 172.01)	1.39 (0.04, 49.99)	2.87 (0.24, 35.82)	29.85 (2.34, 372.12)
0.69 (0.01, 61.01)	9.13 (0.06, 1433.58)	0.99 (0.01, 151.95)	1.16 (0.01, 176.8)	1.44 (0.04, 51.91)	3 (0.25, 36.06)	30.84 (2.48, 391.21)
0.02 (0, 2)	0.29 (0, 46.82)	0.03 (0, 5.17)	0.04 (0, 6.02)	0.05 (0, 1.73)	0.1 (0.01, 1.34)	B4
0.23 (0, 21.55)	2.95 (0.02, 480.88)	0.32 (0, 52.55)	0.38 (0, 61.76)	0.48 (0.01, 18.32)	B5	10.29 (0.74, 141.6)
0.47 (0.03, 7.22)	6.26 (0.18, 221.6)	0.68 (0.02, 23.35)	0.79 (0.02, 27.73)	D2	2.10 (0.05, 78.42)	21.75 (0.58, 825.47)
0.59 (0.01, 51.11)	7.81 (1.41, 44.4)	0.85 (0.15, 4.72)	F1	1.26 (0.04, 42.86)	2.64 (0.02, 420.98)	26.86 (0.17, 4456.65)
0.69 (0.01, 61.45)	9.25 (1.65, 53.08)	F2	1.18 (0.21, 6.52)	1.47 (0.04, 51.29)	3.12 (0.02, 487.87)	31.85 (0.19, 5161.91)
0.08 (0, 6.75)	F4	0.11 (0.02, 0.6)	0.13 (0.02, 0.71)	0.16 (0, 5.42)	0.34 (0, 53.47)	3.47 (0.02, 553.88)
C1	13.24 (0.15, 1156.16)	1.44 (0.02, 123.81)	1.69 (0.02, 149.55)	2.11 (0.14, 32.23)	4.43 (0.05, 420.83)	45.49 (0.5, 4303.63)
1.78 (0.1, 34.62)	23.61 (0.26, 2084.11)	2.58 (0.03, 224.78)	3.04 (0.03, 258.32)	3.76 (0.24, 56.73)	7.82 (0.08, 748.55)	80.1 (0.83, 7636.11)
0.46 (0.02, 8.95)	6.18 (0.07, 533.53)	0.67 (0.01, 58.17)	0.79 (0.01, 68.92)	0.97 (0.06, 15.09)	2.07 (0.02, 196.41)	21.05 (0.22, 2025.39)
3.96 (0.2, 76.93)	52.06 (0.58, 4627.53)	5.7 (0.06, 496.9)	6.73 (0.08, 605.96)	8.32 (0.54, 129.16)	17.78 (0.18, 1639.21)	[illegible] (1.88, [illegible])
0.1 (0.01, 2.02)	1.4 (0.02, 116.72)	0.15 (0, 12.55)	0.18 (0, 14.54)	0.22 (0.01, 3.25)	0.46 (0, 41.94)	4.68 (0.05, 439.36)
0.43 (0.02, 8.42)	5.73 (0.06, 508.11)	0.62 (0.01, 54.1)	0.74 (0.01, 63.04)	0.91 (0.06, 13.99)	1.89 (0.02, 183.22)	19.87 (0.21, 1889.72)
0.4 (0.02, 7.96)	5.38 (0.06, 476.02)	0.59 (0.01, 50.8)	0.7 (0.01, 58.97)	0.85 (0.06, 13.1)	1.79 (0.02, 167.68)	18.39 (0.19, 1737.34)
0.26 (0.01, 4.38)	3.42 (0.06, 192.33)	0.37 (0.01, 20.61)	0.43 (0.01, 24.53)	0.54 (0.08, 3.78)	1.14 (0.02, 70.31)	11.72 (0.18, 753.62)
0.04 (0, 0.66)	0.49 (0.01, 28.73)	0.05 (0, 3.1)	0.06 (0, 3.53)	0.08 (0.01, 0.58)	0.16 (0, 10.47)	1.69 (0.02, 110.98)

1.15 (0.19, 7.24)	0.73 (0.06, 8.5)	0.68 (0.06, 8.17)	2.86 (0.24, 33.79)	0.07 (0.01, 0.89)	0.63 (0.05, 7.33)	0.16 (0.01, 1.94)
1.39 (0.23, 8.36)	0.88 (0.07, 10.11)	0.83 (0.07, 9.47)	3.44 (0.29, 39.52)	0.09 (0.01, 1.07)	0.76 (0.06, 8.98)	0.2 (0.02, 2.32)
2.29 (0.37, 14)	1.45 (0.12, 17.44)	1.37 (0.11, 15.72)	5.68 (0.48, 66.08)	0.15 (0.01, 1.85)	1.27 (0.1, 15.01)	0.33 (0.03, 3.84)
3.93 (0.64, 24.25)	2.51 (0.21, 28.96)	2.34 (0.19, 27.21)	9.69 (0.84, 112.28)	0.26 (0.02, 3.09)	2.17 (0.18, 25.41)	0.57 (0.05, 6.49)
1.36 (0.07, 26.2)	0.84 (0.03, 26.72)	0.80 (0.03, 24.03)	3.34 (0.11, 101.88)	0.09 (0, 2.66)	0.75 (0.02, 22.77)	0.20 (0.01, 5.78)
3.65 (0.19, 71.23)	2.30 (0.08, 73.16)	2.16 (0.07, 64.26)	8.96 (0.31, 277.01)	0.23 (0.01, 7.13)	2.02 (0.07, 63.16)	0.53 (0.02, 16.12)
2.62 (0.13, 52.57)	1.65 (0.05, 51.71)	1.56 (0.05, 47.51)	6.58 (0.21, 200.01)	0.17 (0.01, 5.21)	1.47 (0.05, 43.72)	0.38 (0.01, 11.31)
2.49 (0.13, 48.58)	1.60 (0.05, 48.19)	1.49 (0.05, 44.77)	6.18 (0.21, 189.58)	0.16 (0.01, 4.97)	1.39 (0.05, 41.96)	0.36 (0.01, 10.55)
2.38 (0.12, 47.36)	1.50 (0.05, 46.83)	1.41 (0.05, 42.1)	5.93 (0.19, 182.36)	0.15 (0, 4.79)	1.32 (0.04, 39.75)	0.35 (0.01, 10.01)
4.79 (0.24, 96.6)	3.02 (0.1, 95.55)	2.83 (0.09, 87.32)	11.76 (0.39, 353.03)	0.31 (0.01, 9.65)	2.63 (0.08, 81.12)	0.68 (0.02, 20.68)
2.28 (0.04, 139.04)	1.45 (0.02, 132.31)	1.35 (0.02, 125.44)	5.7 (0.06, 536.05)	0.15 (0, 14.21)	1.26 (0.01, 118.47)	0.33 (0, 30.11)
2.56 (0.04, 149.92)	1.62 (0.02, 148.64)	1.53 (0.02, 134.8)	6.26 (0.07, 572.75)	0.16 (0, 15.23)	1.43 (0.01, 127.28)	0.37 (0, 33.99)
2.67 (0.05, 154.12)	1.67 (0.02, 149.65)	1.56 (0.02, 142.02)	6.57 (0.07, 580.67)	0.17 (0, 15.89)	1.46 (0.02, 136.05)	0.38 (0, 35.03)
0.09 (0, 5.47)	0.05 (0, 5.37)	0.05 (0, 4.8)	0.21 (0, 20.01)	0.01 (0, 0.53)	0.05 (0, 4.61)	0.01 (0, 1.21)
0.88 (0.01, 54.67)	0.56 (0.01, 51.17)	0.53 (0.01, 50.5)	2.18 (0.02, 214.46)	0.06 (0, 5.51)	0.48 (0.01, 47.37)	0.13 (0, 12.44)
1.85 (0.26, 13.02)	1.18 (0.08, 17.8)	1.10 (0.07, 16.86)	4.57 (0.31, 70.28)	0.12 (0.01, 1.86)	1.03 (0.07, 15.53)	0.27 (0.02, 4.1)
2.31 (0.04, 132.49)	1.43 (0.02, 131.5)	1.35 (0.02, 115.94)	5.69 (0.07, 501.17)	0.15 (0, 13.16)	1.27 (0.01, 113.44)	0.33 (0, 29.29)
2.72 (0.05, 156.72)	1.70 (0.02, 157.81)	1.62 (0.02, 138.95)	6.67 (0.08, 593.69)	0.18 (0, 15.74)	1.49 (0.02, 136.42)	0.39 (0, 33.45)
0.29 (0.01, 16.9)	0.19 (0, 16.49)	0.17 (0, 15.42)	0.71 (0.01, 63.15)	0.02 (0, 1.73)	0.16 (0, 14.55)	0.04 (0, 3.79)
3.91 (0.23, 68.14)	2.48 (0.13, 48.64)	2.31 (0.12, 46.24)	9.6 (0.5, 183.98)	0.25 (0.01, 4.97)	2.18 (0.11, 41.54)	0.56 (0.03, 10.52)
6.98 (0.4, 121.88)	4.4 (0.23, 83.43)	4.15 (0.21, 78.76)	17.22 (0.92, 333.62)	0.45 (0.02, 9.12)	3.88 (0.19, 74.49)	C2
1.8 (0.1, 32.28)	1.14 (0.06, 22.34)	1.07 (0.05, 21.23)	4.46 (0.23, 84.96)	0.12 (0.01, 2.36)	C3	0.26 (0.01, 5.16)
15.36 (0.87, 272.6)	9.77 (0.5, 190.33)	9.14 (0.45, 176.2)	38.52 (1.89, 732.66)	C4	8.69 (0.42, 165.91)	2.21 (0.11, 43.4)
0.41 (0.02, 7.24)	0.26 (0.01, 4.94)	0.24 (0.01, 4.67)	C5	0.03 (0, 0.53)	0.22 (0.01, 4.42)	0.06 (0, 1.09)
1.67 (0.1, 30.1)	1.07 (0.05, 20.5)	G1	4.19 (0.21, 78.32)	0.11 (0.01, 2.21)	0.94 (0.05, 18.19)	0.24 (0.01, 4.71)
1.58 (0.09, 29.15)	G4	0.93 (0.05, 18.3)	3.92 (0.2, 73.17)	0.1 (0.01, 2)	0.88 (0.04, 16.94)	0.23 (0.01, 4.3)
L5	0.63 (0.03, 11.02)	0.6 (0.03, 10.5)	2.47 (0.14, 43.76)	0.07 (0, 1.15)	0.56 (0.03, 9.63)	0.14 (0.01, 2.52)
0.15 (0.02, 1.25)	0.09 (0, 1.67)	0.09 (0, 1.54)	0.36 (0.02, 6.44)	0.01 (0, 0.17)	0.08 (0, 1.5)	0.02 (0, 0.37)

7.9 (1.22, 52.09)
9.56 (1.48, 61.48)
15.8 (2.43, 102.14)
27.05 (4.13, 181.13)
9.37 (0.46, 194.06)
25.12 (1.22, 520.32)
18.14 (0.89, 381.68)
17.4 (0.86, 352.75)
16.42 (0.8, 337.97)
32.61 (1.62, 683.31)
15.84 (0.26, 985.13)
17.61 (0.29, 1159.01)
18.18 (0.3, 1146.1)
0.59 (0.01, 40.09)
6.11 (0.1, 389.86)
12.61 (1.73, 97.93)
15.93 (0.28, 990.26)
18.54 (0.32, 1165)
2.04 (0.03, 123.68)
26.81 (1.51, 501.13)
48.04 (2.68, 886.22)
12.43 (0.67, 236.4)
[illegible] (5.81, [illegible])
2.77 (0.16, 50.57)
11.53 (0.65, 222.08)
10.87 (0.6, 204.61)
6.86 (0.8, 58.65)
L6

Note:

1. The letters in the table represent different genes, and the Numbers after the letters represent different genetic models.

2. (A) XRCC3 rs861539; (B)XRCC2 rs718282; (C)SRD5A2 rs523349; (D) RAD51 rs1801320; (E)p16/CDKN2 rs11515; (F)MTHFR rs1801131; (G)BRCA2 rs144848; (L)XRCC3 rs1799796.

3. No.1: allele gene model; No.2: Homozygous genes; No.3: Heterozygous gene model; No.4: Recessive gene model; No.5: Dominant gene model; No.6: Additive gene model.

Table 2. A network of intergene associations.

H1	2.34 (1.88, 2.91)	2.9 (2.33, 3.6)	1.5 (1.21, 1.85)	1.31 (1.11, 1.52)	0.9 (0.75, 1.07)	2.63 (2.22, 3.11)	3.53 (2.98, 4.17)	0.38 (0.32, 0.45)
0.43 (0.34, 0.53)	H2	1.25 (0.97, 1.6)	0.64 (0.5, 0.82)	0.56 (0.46, 0.69)	0.39 (0.31, 0.48)	1.12 (0.91, 1.39)	1.51 (1.23, 1.87)	0.16 (0.13, 0.2)
0.34 (0.28, 0.43)	0.8 (0.62, 1.03)	H3	0.51 (0.41, 0.66)	0.45 (0.37, 0.55)	0.31 (0.25, 0.39)	0.9 (0.74, 1.12)	1.21 (0.99, 1.5)	0.13 (0.11, 0.16)
0.67 (0.54, 0.83)	1.56 (1.22, 2)	1.94 (1.51, 2.46)	H4	0.87 (0.71, 1.05)	0.6 (0.49, 0.74)	1.75 (1.43, 2.14)	2.36 (1.92, 2.86)	0.25 (0.2, 0.31)
0.77 (0.66, 0.9)	1.79 (1.46, 2.19)	2.23 (1.81, 2.71)	1.15 (0.95, 1.4)	K1	0.69 (0.62, 0.78)	2.01 (1.82, 2.23)	2.7 (2.46, 3)	0.29 (0.26, 0.32)
1.11 (0.93, 1.33)	2.59 (2.07, 3.24)	3.22 (2.59, 3.97)	1.66 (1.34, 2.05)	1.45 (1.28, 1.62)	K2	2.91 (2.55, 3.3)	3.91 (3.44, 4.45)	0.42 (0.36, 0.48)
0.38 (0.32, 0.45)	0.89 (0.72, 1.1)	1.11 (0.89, 1.36)	0.57 (0.47, 0.7)	0.5 (0.45, 0.55)	0.34 (0.3, 0.39)	K3	1.34 (1.2, 1.5)	0.14 (0.13, 0.16)
0.28 (0.24, 0.34)	0.66 (0.54, 0.82)	0.83 (0.67, 1.01)	0.42 (0.35, 0.52)	0.37 (0.33, 0.41)	0.26 (0.22, 0.29)	0.74 (0.67, 0.83)	K4	0.11 (0.09, 0.12)
2.64 (2.23, 3.16)	6.16 (4.96, 7.67)	7.68 (6.2, 9.47)	3.95 (3.24, 4.88)	3.45 (3.08, 3.84)	2.38 (2.08, 2.74)	6.93 (6.14, 7.82)	9.31 (8.25, 10.58)	K5

Note:

1. The letters in the table represent different genes, and the Numbers after the letters represent different genetic models.

2. (H)Bsm1 rs1544410; (K)Fok1 rs2228570.

3. No.1: allele gene model; No.2: Homozygous genes; No.3: Heterozygous gene model; No.4: Recessive gene model; No.5: Dominant gene model; No.6: Additive gene model.

Table 3. A network of intergene associations.

S1	3.59 (0.18, 69.1)	4.51 (0.24, 85.7)	2.7 (0.14, 51.1)	4.54 (0.25, 84.44)	11.93 (0.64, 221.6)	22.29 (1.2, 397.63)	2.72 (0.14, 51.79)	2.52 (0.13, 47.38)	5.25 (0.26, 101.97)	2.38 (0.12, 43.93)	1.42 (0.07, 27.05)	23.7 (1.24, 434.3)	13.91 (0.75, 261.7)	13.05 (0.72, 238.83)
0.28 (0.01, 5.42)	S5	1.24 (0.07, 22.75)	0.75 (0.04, 13.87)	1.26 (0.07, 23.25)	3.35 (0.18, 62.21)	6.13 (0.34, 112.72)	0.74 (0.04, 14.25)	0.7 (0.04, 12.98)	1.46 (0.08, 27.4)	0.66 (0.04, 12.38)	0.39 (0.02, 7.29)	6.52 (0.35, 123.68)	3.87 (0.21, 71.4)	3.6 (0.19, 68.9)
0.22 (0.01, 4.1)	0.8 (0.04, 14.53)	T1	0.6 (0.03, 11.02)	1 (0.06, 18.23)	2.65 (0.14, 48.51)	4.92 (0.27, 88.61)	0.6 (0.03, 11.23)	0.56 (0.03, 10.25)	1.16 (0.06, 22.17)	0.52 (0.03, 9.87)	0.31 (0.02, 6.04)	5.23 (0.29, 96.84)	3.07 (0.17, 56.9)	2.87 (0.16, 53.27)
0.37 (0.02, 7.22)	1.34 (0.07, 25.7)	1.67 (0.09, 32.22)	T2	1.69 (0.09, 32.22)	4.45 (0.23, 85.34)	8.21 (0.44, 152.43)	1.01 (0.05, 19.25)	0.94 (0.05, 17.67)	1.94 (0.1, 37.04)	0.88 (0.05, 16.77)	0.52 (0.03, 10.05)	8.79 (0.45, 170.56)	5.18 (0.29, 98.09)	4.89 (0.26, 89.73)
0.22 (0.01, 4.06)	0.79 (0.04, 14.55)	1 (0.05, 18.17)	0.59 (0.03, 11.25)	T4	2.62 (0.14, 48.99)	4.83 (0.27, 92.61)	0.59 (0.03, 11.1)	0.56 (0.03, 10.47)	1.15 (0.06, 21.79)	0.52 (0.03, 9.79)	0.31 (0.02, 5.77)	5.14 (0.28, 96.77)	3.05 (0.17, 57.98)	2.85 (0.16, 54.85)
0.08 (0, 1.57)	0.3 (0.02, 5.68)	0.38 (0.02, 7.01)	0.22 (0.01, 4.31)	0.38 (0.02, 7.08)	T5	1.83 (0.1, 34.55)	0.23 (0.01, 4.16)	0.21 (0.01, 3.77)	0.44 (0.02, 8.33)	0.2 (0.01, 3.76)	0.12 (0.01, 2.18)	1.96 (0.11, 36.42)	1.17 (0.06, 21.69)	1.08 (0.06, 21.49)
0.04 (0, 0.84)	0.16 (0.01, 2.94)	0.2 (0.01, 3.77)	0.12 (0.01, 2.29)	0.21 (0.01, 3.72)	0.55 (0.03, 10.16)	U1	0.12 (0.01, 2.34)	0.11 (0.01, 2.1)	0.24 (0.01, 4.47)	0.11 (0.01, 2)	0.06 (0, 1.21)	1.06 (0.06, 20.42)	0.62 (0.04, 12.04)	0.59 (0.03, 10.98)
0.37 (0.02, 7.18)	1.34 (0.07, 25.68)	1.68 (0.09, 31.37)	0.99 (0.05, 19.33)	1.69 (0.09, 31.52)	4.44 (0.24, 82.49)	8.21 (0.43, 150.67)	U2	0.93 (0.05, 17.31)	1.95 (0.11, 36.04)	0.87 (0.05, 16.48)	0.51 (0.03, 9.83)	8.76 (0.47, 161.5)	5.14 (0.28, 96.76)	4.83 (0.26, 92.78)

0.4 (0.02, 7.51)	1.42 (0.08, 26.66)	1.78 (0.1, 34.17)	1.06 (0.06, 20.39)	1.8 (0.1, 33.99)	4.73 (0.27, 86.55)	8.76 (0.48, 161.33)	1.08 (0.06, 19.63)	U5	2.07 (0.11, 38.57)	0.93 (0.05, 17.18)	0.56 (0.03, 10.33)	9.35 (0.52, 174.66)	5.48 (0.31, 103.47)	5.15 (0.29, 96.14)
0.19 (0.01, 3.78)	0.69 (0.04, 13.23)	0.86 (0.05, 16.35)	0.52 (0.03, 9.95)	0.87 (0.05, 16.73)	2.29 (0.12, 41.98)	4.25 (0.22, 79.78)	0.51 (0.03, 9.48)	0.48 (0.03, 8.93)	V1	0.45 (0.02, 9.05)	0.27 (0.01, 5.23)	4.52 (0.25, 82.62)	2.67 (0.15, 48.49)	2.48 (0.14, 47.11)
0.42 (0.02, 8.19)	1.52 (0.08, 28.07)	1.91 (0.1, 34.94)	1.13 (0.06, 21.67)	1.94 (0.1, 35.47)	5.13 (0.27, 94.06)	9.35 (0.5, 171.93)	1.15 (0.06, 21.37)	1.07 (0.06, 20.56)	2.22 (0.11, 41.81)	V2	0.59 (0.03, 11.24)	10 (0.57, 189.97)	5.89 (0.32, 110.22)	5.5 (0.3, 104.39)
0.71 (0.04, 13.72)	2.57 (0.14, 47.97)	3.23 (0.17, 59.56)	1.94 (0.1, 35.85)	3.21 (0.17, 60.45)	8.54 (0.46, 162.91)	15.74 (0.83, 290.32)	1.94 (0.1, 36.64)	1.79 (0.1, 33.94)	3.73 (0.19, 70.6)	1.69 (0.09, 32.12)	V5	16.79 (0.9, 317.04)	9.95 (0.54, 192.77)	9.22 (0.53, 174.15)
0.04 (0, 0.8)	0.15 (0.01, 2.85)	0.19 (0.01, 3.5)	0.11 (0.01, 2.2)	0.19 (0.01, 3.58)	0.51 (0.03, 9.28)	0.95 (0.05, 17.14)	0.11 (0.01, 2.11)	0.11 (0.01, 1.92)	0.22 (0.01, 4.06)	0.1 (0.01, 1.74)	0.06 (0, 1.11)	W1	0.59 (0.03, 11.09)	0.55 (0.03, 10.11)
0.07 (0, 1.34)	0.26 (0.01, 4.72)	0.33 (0.02, 5.77)	0.19 (0.01, 3.44)	0.33 (0.02, 5.95)	0.86 (0.05, 15.46)	1.6 (0.08, 28.37)	0.19 (0.01, 3.57)	0.18 (0.01, 3.25)	0.38 (0.02, 6.67)	0.17 (0.01, 3.09)	0.1 (0.01, 1.86)	1.68 (0.09, 29.92)	W2	0.93 (0.05, 17.22)
0.08 (0, 1.4)	0.28 (0.01, 5.19)	0.35 (0.02, 6.35)	0.2 (0.01, 3.79)	0.35 (0.02, 6.27)	0.93 (0.05, 16.53)	1.69 (0.09, 31.29)	0.21 (0.01, 3.83)	0.19 (0.01, 3.47)	0.4 (0.02, 7.27)	0.18 (0.01, 3.3)	0.11 (0.01, 1.89)	1.81 (0.1, 32.95)	1.07 (0.06, 19.56)	W5

Note:

1. The letters in the table represent different genes, and the Numbers after the letters represent different genetic models.

2. (S)GALNT6 rs907352;(T)GALNT7 rs934358;(U)MGAT5 rs1257187;(V)ST3GAL3 rs3828139;(W)ST3GAL3 rs37460.

3. No.1: allele gene model;No.2: Homozygous genes;No.3: Heterozygous gene model;No.4: Recessive gene model;No.5: Dominant gene model;No.6: Additive gene model.

Table 4. A network of intergene associations.

Q5	1.87 (0.65, 5.33)	1.13 (0.32, 3.1)	3.18 (1.08, 9.09)	4.49 (1.53, 12.62)	0.54 (0.16, 1.49)
0.54 (0.19, 1.54)	J1	0.61 (0.18, 1.62)	1.69 (0.6, 4.76)	2.4 (0.84, 6.76)	0.29 (0.09, 0.79)
0.88 (0.32, 3.11)	1.64 (0.62, 5.66)	J2	2.79 (1.02, 9.72)	3.97 (1.43, 13.57)	0.48 (0.15, 1.55)
0.31 (0.11, 0.93)	0.59 (0.21, 1.66)	0.36 (0.1, 0.98)	J3	1.42 (0.49, 4.04)	0.17 (0.05, 0.48)
0.22 (0.08, 0.65)	0.42 (0.15, 1.19)	0.25 (0.07, 0.7)	0.7 (0.25, 2.05)	J4	0.12 (0.04, 0.33)
1.85 (0.67, 6.33)	3.45 (1.27, 11.54)	2.1 (0.64, 6.61)	5.87 (2.1, 19.81)	8.27 (3.04, 28.14)	J5

Note:

1. The letters in the table represent different genes, and the Numbers after the letters represent different genetic models.

2.(J)miR-146a rs2910164; (Q)miR-196a2 rs11614913.

3. No.1: allele gene model; No.2: Homozygous genes; No.3: Heterozygous gene model; No.4: Recessive gene model; No.5: Dominant gene model; No.6: Additive gene model.

Table 5. A network of intergene associations.

M2	0.55 (0.4, 0.76)	1.54 (1.15, 2.04)	0.57 (0.38, 0.85)	0.35 (0.23, 0.52)	5.26 (3.54, 7.93)
1.81 (1.31, 2.53)	M5	2.79 (2.11, 3.69)	1.04 (0.7, 1.55)	0.64 (0.43, 0.95)	9.53 (6.49, 14.31)
0.65 (0.49, 0.87)	0.36 (0.27, 0.47)	M1	0.37 (0.26, 0.54)	0.23 (0.16, 0.33)	3.42 (2.42, 4.95)
1.74 (1.17, 2.61)	0.96 (0.64, 1.43)	2.68 (1.85, 3.87)	N2	0.61 (0.42, 0.89)	9.18 (5.67, 15.05)
2.84 (1.92, 4.3)	1.56 (1.06, 2.34)	4.37 (3.04, 6.35)	1.63 (1.12, 2.38)	N5	14.97 (9.29, 24.57)
0.19 (0.13, 0.28)	0.1 (0.07, 0.15)	0.29 (0.2, 0.41)	0.11 (0.07, 0.18)	0.07 (0.04, 0.11)	N6

Note:

1. The letters in the table represent different genes, and the Numbers after the letters represent different genetic models.

2. (M)ERCC1 rs3212986; (N)ERCC1 rs11615.

3. No.1: allele gene model; No.2: Homozygous genes; No.3: Heterozygous gene model; No.4: Recessive gene model; No.5: Dominant gene model; No.6: Additive gene model.
