# Supplementary material for: The association between single nucleotide polymorphisms and ovarian cancer risk: A systematic review and network meta‐analysis
Source: Cancer Med. 2022 May 30;12(1):541–56. doi: 10.1002/cam4.4891 (PMC9844622; doi:10.1002/cam4.4891)
Supplement: Supplementary file 7 — Supplement Information S7 [file CAM4-12-541-s003.pdf]

Supplement information 7. Venice criteria for assessing cumulative epidemiologic evidence in genetic associations.

Gene	Genetic model	Odd Ratios (95% CI)	Amount of study	Amount of evidence	Replication (I^2)	Protection from bias	Credibility of evidence
SRD5A2 rs523349	CC+GC vs GG	1.305 (1.132-1.504)	2	221(B)	0.0%(A)	B	moderate
Fok1 rs2228570	TT+CT vs CC	1.158 (1.068-1.256)	16	674(B)	22.2%(A)	B	moderate
ST3GAL3 rs37460	C vs G	0.853 (0.747-0.974)	2	164(B)	0.0%(A)	B	moderate
miR-146a rs2910164	CC+GC vs GG	0.293 (0.121-0.709)	3	57(C)	80.4%(A)	B	weak
ERCC1 rs11615	GG+AA vs GA	1.696 (1.024-2.810)	2	19(C)	58.7%(A)	B	weak
